# Chemical and Electrochemical Investigation of the Oxidation of a Highly Reduced Fe_6_C Iron Carbide Carbonyl Cluster: A Synthetic Route to Heteroleptic Fe_6_C and Fe_5_C Clusters

**DOI:** 10.1021/acs.inorgchem.5c01014

**Published:** 2025-05-06

**Authors:** Tiziana Funaioli, Cristiana Cesari, Beatrice Berti, Marco Bortoluzzi, Cristina Femoni, Francesca Forti, Maria Carmela Iapalucci, Giorgia Scorzoni, Stefano Zacchini

**Affiliations:** † Dipartimento di Chimica e Chimica Industriale, Università di Pisa, Via G. Moruzzi 13, 56124 Pisa, Italy; ‡ Dipartimento di Chimica Industriale “Toso Montanari”, Università di Bologna, Via P. Gobetti 85, 40129 Bologna, Italy; § Dipartimento di Scienze Molecolari e Nanosistemi, Ca’ Foscari University of Venice, Via Torino 155, 30175 Mestre (Ve), Italy

## Abstract

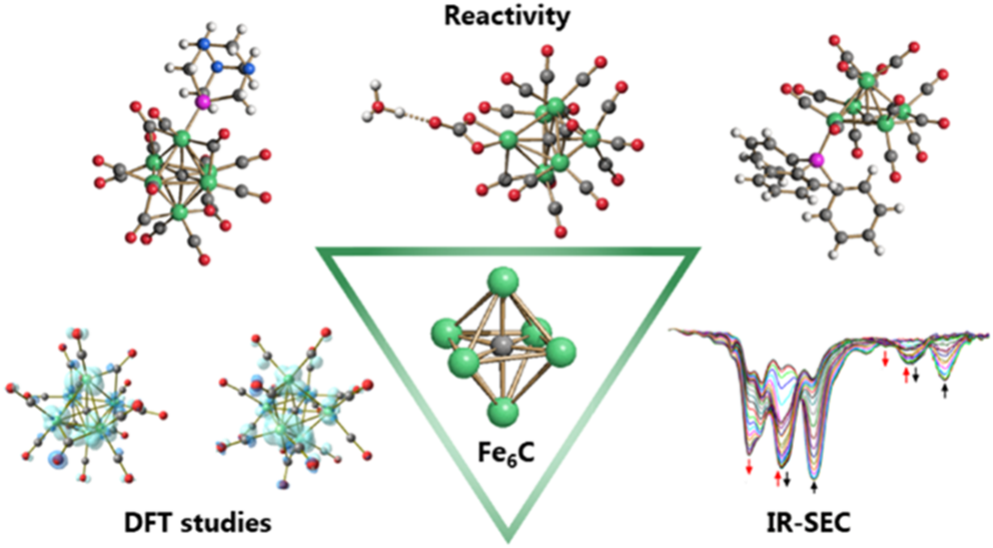

A chemical and electrochemical investigation of the redox chemistry of [Fe_6_C(CO)_15_]^4–^ is reported and supported by computational studies. Depending on the experimental conditions, the original Fe_6_C cage is retained or partially degraded to Fe_5_C. Chemical oxidation of [Fe_6_C(CO)_15_]^4–^ with [Cp_2_Fe][PF_6_], [C_7_H_7_][BF_4_], or Me_3_NO affords the previously reported [Fe_6_C(CO)_16_]^2–^, whereas oxidation in the presence of a base (Na_2_CO_3_ or NaOH) results in the new carbonate-carbide cluster [Fe_6_C(CO)_14_(CO_3_)]^4–^. Oxidation of [Fe_6_C(CO)_15_]^4–^ in the presence of a phosphine ligand produces the heteroleptic species [Fe_6_C(CO)_15_(PTA)]^2–^ and [Fe_5_C(CO)_13_(PPh_3_)]^2–^. Reaction of [Fe_6_C(CO)_15_]^4–^ with alkylating or acylating agents (MeI, CF_3_SO_3_Me, and MeCOCl) affords the acetyl-carbide cluster [Fe_5_C(CO)_13_(COMe)]^3–^, with partial oxidative degradation of the original Fe_6_C cage. The new clusters have been spectroscopically and structurally characterized. The redox chemistry of [Fe_6_C(CO)_15_]^4–^ was further investigated by electrochemical and spectroelectrochemical methods. According to computational outcomes, the spectroelectrochemical oxidation of [Fe_6_C(CO)_15_]^4–^ follows an EEC mechanism, leading to the formation of [Fe_6_C(CO)_16_]^2–^. The [Fe_6_C(CO)_15_]^3–^ intermediate can accumulate and be spectroscopically detected. These new chemical and electrochemical findings have been supported and corroborated by computational methods. DFT calculations suggest an EEC pathway also for the reverse electrochemical process, *i.e.*, reduction of [Fe_6_C(CO)_16_]^2–^ to [Fe_6_C(CO)_15_]^4–^.

## Abstract 2

Oxidation of a highly reduced Fe_6_C cluster has been investigated as a platform for the synthesis of functionalized hexa-iron-carbide clusters.

## 1 Introduction

Iron carbide carbonyl clusters played a crucial role in the development of the chemistry of molecular metal clusters encapsulating light p-block atoms.^1−3^ The unique C atom may be fully interstitial within an octahedral cage, as found in [Fe_6_C(CO)_16_]^2–^,^4^^4^ and [Fe_6_C(CO)_15_]^4–^,^5^ or semiexposed in a butterfly environment, e.g., Fe_4_C(CO)_13_,^6^ [Fe_4_C(CO)_12_]^2–^,^7^ and [HFe_4_C(CO)_12_]^−^,^8^ or in a square-pyramidal structure, e.g., Fe_5_C(CO)_15_^1^ and [Fe_5_C(CO)_14_]^2–^.^9^ The presence of additional Fe–C bonds makes iron carbide carbonyl clusters more robust than noncarbide species. Due to this enhanced stability, iron carbide carbonyl clusters display a rich chemistry.^10−13^

Earlier interest in iron carbide carbonyl clusters was mainly due to the possibility of exploiting semiexposed carbides for the formation of C–C and C–H bonds, mimicking the fundamental steps occurring at the surface of heterogeneous catalysts during industrial processes such as the Fischer–Tropsch synthesis.^14−20^ In this respect, iron carbide carbonyl clusters greatly contributed to the development of the cluster-surface analogy.^21,22^ More recently, renewed interest in iron carbide carbonyl clusters has been boosted by two major discoveries. First, Berben et al. demonstrated that tetranuclear iron carbide carbonyl clusters are very active electrocatalysts for the hydrogen evolution reaction (HER).^23,24^ Then, the discovery that the FeMoco cofactor of nitrogenase actually contained a Fe_6_C carbide core generated the so-called “carbide problem”, that is, the problem of chemically synthesizing FeS ensembles incorporating fully inorganic carbides, trying to mimic the biosynthesis of FeMoco.^25−30^ An analogous Fe_6_C carbide core was later discovered also in the structure of the iron-only Fe-nitrogenase.^31^ One possibility to tackle the carbide problem could be to start with a preformed iron carbide cluster and, in this sense, studies have been dedicated to the addition of sulfur to [Fe_6_C(CO)_16_]^2–^.^32−36^

Inorganic sulfur may be directly introduced into [Fe_6_C(CO)_16_]^2–^ using S_8_ and S_2_Cl_2_.^29^ Alternatively, one CO ligand can be replaced by SO_2_ and, then, the O atom chemically removed from [Fe_6_C(CO)_15_(SO_2_)]^2–^ eventually resulting in [Fe_6_C(CO)_14_(S)]^2–^.^36^ The introduction of organic sulfur is more difficult and, to date, the only report appeared in the literature described the reaction of [Fe_6_C(CO)_16_]^2–^ with *p*-Me-C_6_H_4_–SCl affording [Fe_5_C(CO)_13_(S–C_6_H_4_Me)]^2–^.^29^ Nonetheless, this approach resulted in partial degradation of the Fe_6_C cage to Fe_5_C. This is likely due to the electrophilic nature of the organosulfur reagent. For instance, it is known that the reaction of [Fe_6_C(CO)_16_]^2–^ with H^+^ ions results in the elimination of Fe^2+^ and the formation of [Fe_5_C(CO)_14_]^2–^.^3^ The overall reaction may be described as an oxidative degradation of the Fe_6_C cage.

The coordination of organosulfur ligands or other organic ligands to the Fe_6_C cage could be of interest not only in order to develop models of nitrogenase cofactor, but also for further applications of functionalized Fe-carbide-carbonyl clusters as for instance in electrocatalysis.^37,38^ In this regard, a better understanding of the redox and electrochemical properties of such clusters might be useful, particularly focusing on the conditions for the retention of the Fe_6_C cage, compared to its partial degradation to Fe_5_C.

The species [Fe_6_C(CO)_16_]^2–^ is highly inert toward ligand substitution and, thus, suitable protocols should be developed in order to introduce organic ligands without degradation of its Fe_6_C cage. Very recently, Rose et al. have developed a method to accomplish CO to PR_3_ substitution via oxidation of [Fe_6_C(CO)_16_]^2–^.^39^ Our group reported some years ago the chemical reduction of [Fe_6_C(CO)_16_]^2–^ affording the highly reduced [Fe_6_C(CO)_15_]^4–^ octahedral carbide cluster.^5^ More recently, we have reported the synthesis of the [Ru_6_C(CO)_15_]^4–^ analogue, together with a detailed investigation of its chemical and electrochemical oxidation.^40^ This highly reduced ruthenium carbide carbonyl cluster was revealed to be an interesting starting material for the synthesis of new clusters via redox reactions. Important information on the chemical reactivity of [Ru_6_C(CO)_15_]^4–^ was also obtained by joint electrochemical, IR spectroelectrochemical, and computational studies.

Up to now, only the reactivity of [Fe_6_C(CO)_15_]^4–^ toward electrophiles has been explored. Its reaction with [Au(PPh_3_)Cl] affords the [Fe_6_C(CO)_15_(AuPPh_3_)_2_]^2–^ adduct, whereas addition of strong acids, such as HBF_4_·Et_2_O, results in the labile hydride [HFe_6_C(CO)_15_]^3–^.^5^ The latter species is rather unstable and rapidly evolves into [Fe_6_C(CO)_16_]^2–^. Overall, [Fe_6_C(CO)_15_]^4–^ is oxidized by acids to [Fe_6_C(CO)_16_]^2–^. Further addition of acids affords [Fe_5_C(CO)_14_]^2–^, in keeping with the well-known reactivity of [Fe_6_C(CO)_16_]^2–^ with acids as summarized above.

The main aim of this paper is to investigate the selective oxidation of [Fe_6_C(CO)_15_]^4–^, particularly focusing on the conditions to avoid degradation of its Fe_6_C cage to Fe_5_C. For this purpose, chemical electrochemical and IR spectroelectrochemical (IR-SEC) methods have been employed and further supplemented by computational studies. Moreover, the synthetic potentiality of the oxidation of [Fe_6_C(CO)_15_]^4–^ has been explored in order to obtain new heteroleptic iron carbide carbonyl clusters. Also in this case, particular focus has been given to shed light on the conditions for retaining the intact Fe_6_C cage, compared to its degradation to Fe_5_C clusters.

The new compounds have been characterized by spectroscopic (IR, multinuclear NMR) and single-crystal X-ray diffraction (SC-XRD) methods. The redox chemistry of [Fe_6_C(CO)_15_]^4–^ was investigated by cyclic voltammetry (CV) and IR spectroelectrochemistry (IR-SEC). Since detailed IR-SEC studies of the parent [Fe_6_C(CO)_16_]^2–^ have not been previously reported, they have been herein included for comparative purposes, in view also of the fact that [Fe_6_C(CO)_16_]^2–^ is often produced along the chemical and electrochemical oxidation of [Fe_6_C(CO)_15_]^4–^. Computational methods by DFT calculations were employed to support the new findings.

## 2 Results and Discussion

### 2.1 Oxidation of [Fe_6_C(CO)_15_]^4–^ in the Absence and in the Presence of Bases: Synthesis and Molecular Structure of [Fe_6_C(CO)_14_(CO_3_)]^4–^

**Scheme 1:**
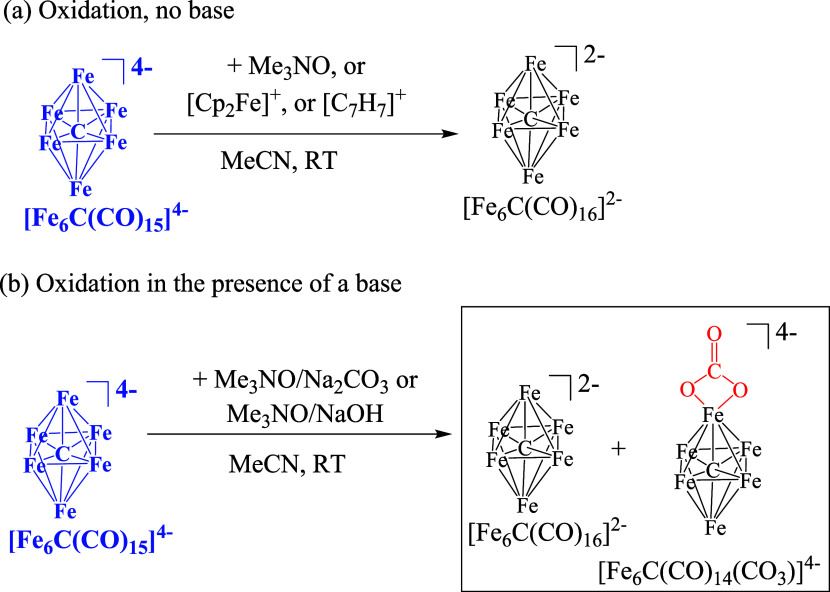
Oxidation Reactions of [Fe_6_C(CO)_15_]^4–^ in MeCN, (a) without and (b) with a Base All of the species have been structurally characterized by SC-XRD. CO ligands have been omitted for clarity. Both [NEt_4_]^+^ and [NMe_3_CH_2_Ph]^+^ can be used as counterions resulting in similar reactions and separation procedures. Oxidants employed: Me_3_NO, [Cp_2_Fe][PF_6_] and [C_7_H_7_][BF_4_].

It was previously reported that [Fe_6_C(CO)_15_]^4–^ was oxidized by strong acids, such as HBF_4_·Et_2_O, to [Fe_6_C(CO)_16_]^2–^ and [Fe_5_C(CO)_14_]^2–^, via the elusive monohydride [HFe_6_C(CO)_15_]^3–^.^5^ In an attempt to avoid partial degradation of the Fe_6_C cage, more innocent oxidants, that is, [Cp_2_Fe][PF_6_], [C_7_H_7_][BF_4_], and Me_3_NO, have been employed in the present study. [NEt_4_]^+^ and [NMe_3_CH_2_Ph]^+^ salts of [Fe_6_C(CO)_15_]^4–^ have been used, leading to very similar results. Indeed, in all cases, the major product of the reactions is the previously reported [Fe_6_C(CO)_16_]^2–^ (Scheme 1), which has been identified based on IR spectroscopy and SC-XRD.

The transformation of [Fe_6_C(CO)_15_]^4–^ into [Fe_6_C(CO)_16_]^2–^ is formally a two-electron oxidation that requires one mole of CO *per* mole of oxidized cluster. Indeed, removal of two electrons from the electron precise [Fe_6_C(CO)_15_]^4–^ (86 CVE, cluster valence electron) would afford an electron-deficient [Fe_6_C(CO)_15_]^2–^ (84 CVE) species, which is not directly observed. It is likely that this purported 84 CVE cluster rapidly decomposes, liberating 15 mol of CO, which can be employed for the formation of [Fe_6_C(CO)_16_]^2–^, avoiding further decomposition. Overall, it is sufficient to decompose 1 mol of this oxidized cluster in order to form 15 mol of [Fe_6_C(CO)_16_]^2–^.

In this regard, it is noteworthy that oxidation of [Ru_6_C(CO)_15_]^4–^ under similar experimental conditions afforded [Ru_6_C(CO)_15_(MeCN)]^2–^, which was sufficiently stable to avoid further decomposition.^40^ Conversely, a related [Fe_6_C(CO)_15_(MeCN)]^2–^ has not been observed in the present study. Such differences between the oxidation behaviors of [Fe_6_C(CO)_15_]^4–^ and [Ru_6_C(CO)_15_]^4–^ prompted further electrochemical and computational studies, which will be described in Sections 2.4 and 2.5, respectively.

[Cp_2_Fe][PF_6_] and [C_7_H_7_][BF_4_] are common oxidants in organometallic chemistry and, thus, it is not surprising their capacity of oxidizing [Fe_6_C(CO)_15_]^4–^ to [Fe_6_C(CO)_16_]^2–^. The fact that the same reaction may be accomplished by Me_3_NO deserves further comments. This is often employed as a decarbonylating agent, requiring nucleophilic attack to a coordinated CO ligand, followed by its oxidation to CO_2_. Such a process is favored in cationic metal carbonyls, more difficult in neutral ones, and very unlikely in anionic species. Thus, it is likely that the oxidation of [Fe_6_C(CO)_15_]^4–^ to [Fe_6_C(CO)_16_]^2–^ operated by Me_3_NO proceeds via a different route. Indeed, it is well-known that Me_3_NO in the presence of moisture (actually hydrated Me_3_NO·2H_2_O is employed along this study) can promote hydrolysis and formation of OH^–^ and [Me_3_NOH]^+^ ions.^41,42^ Protons of [Me_3_NOH]^+^ are probably the actual oxidizing agents through the H^+^/H_2_ redox couple.

Oxidation of [Fe_6_C(CO)_15_]^4–^ has been, then, investigated in the presence of bases, such as Na_2_CO_3_ and NaOH. As in the absence of bases, the outcome of the reaction does not depend on the oxidant employed ([Cp_2_Fe][PF_6_], [C_7_H_7_][BF_4_], and Me_3_NO). In all cases, a mixture of [Fe_6_C(CO)_16_]^2–^ and the new cluster [Fe_6_C(CO)_14_(CO_3_)]^4–^ is obtained.

Na_2_CO_3_ and NaOH are poorly soluble in MeCN, and it is likely that they serve as a base for traces of water to aid the Hieber base reaction. The formal oxidation state of Fe is the same in [Fe_6_C(CO)_16_]^2–^ and [Fe_6_C(CO)_14_(CO_3_)]^4–^, that is, −1/3, assuming the carbide as a neutral ligand (organometallic electron-counting rules). Thus, both clusters are generated by a two-electron oxidation of [Fe_6_C(CO)_15_]^4–^ (Fe, −2/3) which should afford an unstable [Fe_6_C(CO)_15_]^2–^ species, as described above. This can be stabilized upon coordination of CO or CO_3_^2–^ (generated by attack of OH^–^ to CO) leading to [Fe_6_C(CO)_16_]^2–^ and [Fe_6_C(CO)_14_(CO_3_)]^4–^, respectively. Alternatively, adopting coordination chemistry ionic electron-counting rules, the interstitial carbide might be viewed as a tetra-anion, leading to formal oxidation states Fe(0) for [Fe_6_C(CO)_15_]^4–^, and Fe(+1/3) for [Fe_6_C(CO)_14_(CO_3_)]^4–^. Also within this electron-counting scheme, the overall reaction is two-electron oxidation.

**Figure 1:**
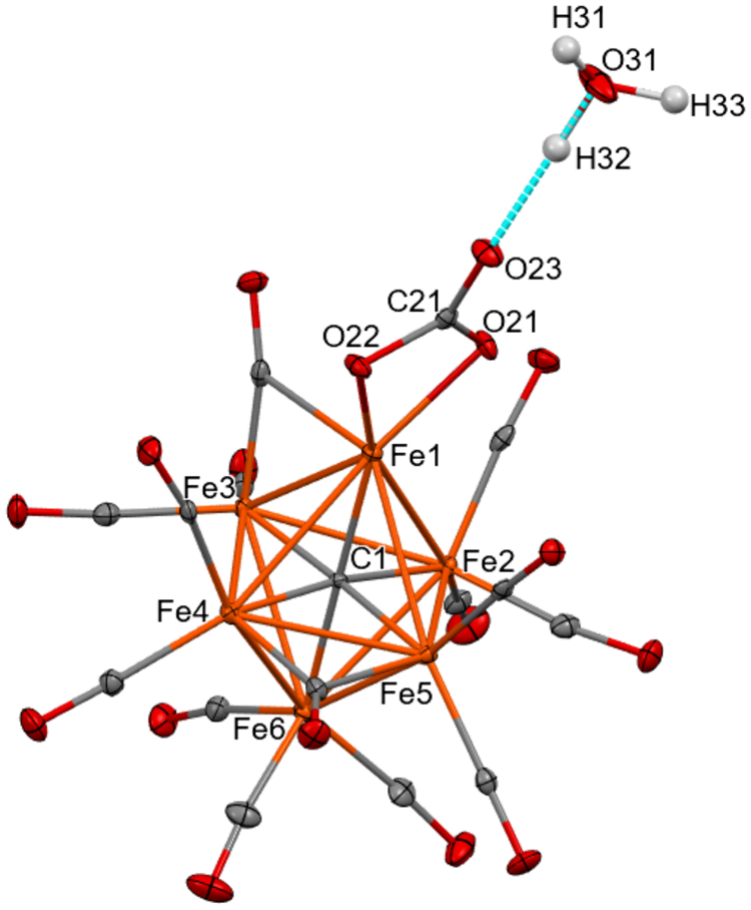
Molecular structure of [Fe_6_C(CO)_14_(CO_3_)]^4–^ as found in [NEt_4_]_3_[H_3_O][Fe_6_C(CO)_14_(CO_3_)]. The H-bond with the [H_3_O]^+^ cation is represented as a fragmented line (orange, Fe; red, O; gray, C; white H). Thermal ellipsoids are at the 30% probability level.

These two products can be separated as [NEt_4_]^+^ or [NMe_3_CH_2_Ph]^+^ salts, since [Fe_6_C(CO)_16_]^2–^ is extracted in THF, and [Fe_6_C(CO)_14_(CO_3_)]^4–^ in acetone. Crystals suitable for SC-XRD analyses of [NEt_4_]_3_[H_3_O][Fe_6_C(CO)_14_(CO_3_)] have been obtained by slow diffusion on *n*-hexane into the acetone solution of [Fe_6_C(CO)_14_(CO_3_)]^4–^, allowing its full structural characterization (Figure 1 and Tables S1 and S2 in the Supporting Information).

The structure of the cluster is characterized by an octahedral geometry, composed of a metallic core in which 6 Fe atoms are present with a carbide located at the center. There are 12 terminal and two edge bridging CO ligands. Moreover, there is a CO_3_^2–^ anion bonded through two O atoms to a single Fe atom. In the solid state, there is a hydrogen bond between the third oxygen of the carbonate group and the [H_3_O]^+^ cation present in the unit cell (Table S3 and Figure S32 in the Supporting Information). The [H_3_O]^+^ cation is H-bonded also to a μ-CO ligand of an adjacent [Fe_6_C(CO)_14_(CO_3_)]^4–^ cluster anion resulting in infinite chains of H-bonded [H_3_O]^+^ and [Fe_6_C(CO)_14_(CO_3_)]^4–^ ions (Figure S32 in the Supporting Information). It is noteworthy that, within the coordinated CO_3_^2–^, the C(21)–O(23) [1.245(2) and 1.244(4) Å for the two independent crystals, respectively] distance in the solid state structure is considerably shorter than C(21)–O(21) [1.305(2) and 1.296(4) Å] and C(21)–O(22) [1.304(2) and 1.307(4) Å] (Table S2 in the Supporting Information) suggesting some π-character and, thus, supporting the hypothesis that the additional proton is bonded to [H_3_O]^+^ within the crystal. Indeed, in the case of compounds containing a κ^2^-O_2_COH ligand bonded to a metal center, usually the C–O(H) bond is longer than the C–O(M) ones.^43−46^

The structure of the carbonate-complex [Fe_6_C(CO)_14_(CO_3_)]^4–^ was DFT-optimized together with that of the related hydrogen carbonate-complex [Fe_6_C(CO)_14_(HCO_3_)]^3–^. Both CO_3_^2–^ and HCO_3_^–^ behave as bidentate ligands (Figure S33 in the Supporting Information), but the computed C–O distances are markedly different between the two clusters. In particular, the average C–O bond lengths involving the coordinating oxygen atoms are 1.313 Å for [Fe_6_C(CO)_14_(CO_3_)]^4–^ and 1.264 Å for [Fe_6_C(CO)_14_(HCO_3_)]^3–^, while the remaining C–O bond is much shorter in [Fe_6_C(CO)_14_(CO_3_)]^4–^ (1.245 Å) with respect to [Fe_6_C(CO)_14_(HCO_3_)]^3–^ (1.341 Å). The X-ray data are more in line with the bond lengths computed for [Fe_6_C(CO)_14_(CO_3_)]^4–^. The Fe–Fe and Fe–C bonding distances of [Fe_6_C(CO)_14_(CO_3_)]^4–^ are only slightly elongated compared to those found in [Fe_6_C(CO)_15_]^4–^ and [Fe_6_C(CO)_16_]^2–^ (Table S1 in the Supporting Information).

Coordination of CO_3_^2–^ to a single metal atom via two oxygen atoms in a metal carbonyl cluster was not reported previously. A few examples have been described where CO_3_^2–^ uses two O atoms to bind three^47^ or four metal atoms,^48^ or where all of the three oxygens are employed to bind different metal atoms.^49−51^

The IR spectrum of [Fe_6_C(CO)_14_(CO_3_)]^4–^ recorded in an acetone solution displays the main ν_CO_ band at 1947 cm^–1^. This is an intermediate value between those of [Fe_6_C(CO)_16_]^2–^ (1966 cm^–1^) and [Fe_6_C(CO)_15_]^4–^ (1875 cm^–1^), suggesting that the cluster adopts a −3 charge in solution. This could be explained by assuming that protonation of the coordinated carbonate ligand occurs in solution, resulting in [Fe_6_C(CO)_14_(HCO_3_)]^3–^. This would correspond to the transfer of a proton from [H_3_O]^+^ to the cluster. In agreement with this hypothesis, the ATR-IR spectrum recorded in the solid state on crystals of [NEt_4_]_3_[H_3_O][Fe_6_C(CO)_14_(CO_3_)] displays ν_CO_ bands at 1925(sh), 1900(vs), 1738(m) cm^–1^, typical of a tetra-anion (Figure S5 in the Supporting Information). A shoulder is observed in the ATR-IR spectrum at 1575–1615 cm^–1^, that can be attributed to the coordinated carbonate ligand of [NEt_4_]_3_[H_3_O][Fe_6_C(CO)_14_(CO_3_)], as further corroborated by computational studies. A band at 1600 cm^–1^ was in fact simulated for [Fe_6_C(CO)_14_(CO_3_)]^4–^ (Figure S33 in the Supporting Information), related to the C–O stretching involving the noncoordinating oxygen atom of the carbonate ligand. The intensity of the simulated vibrations in the same region is negligible for [Fe_6_C(CO)_14_(HCO_3_)]^3–^.

### 2.2 Oxidation of [Fe_6_C(CO)_15_]^4–^ in the Presence of Phosphine Ligands: Synthesis and Molecular Structures of [Fe_6_C(CO)_15_(PTA)]^2–^ and [Fe_5_C(CO)_13_(PPh_3_)]^3–^

**Scheme 2:**
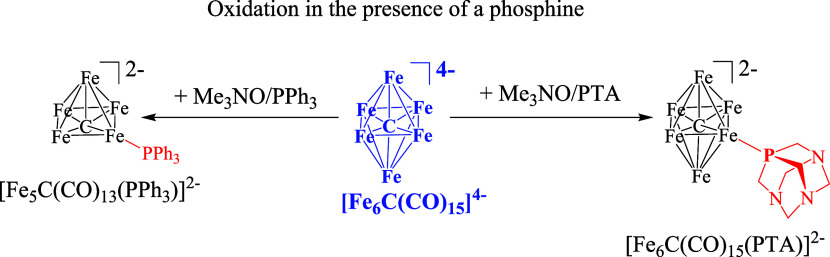
Oxidation Reactions of [Fe_6_C(CO)_15_]^4–^ in MeCN in the Presence of a Phosphine Ligand All of the species have been structurally characterized by SC-XRD. CO ligands have been omitted for clarity. [NEt_4_]^+^ has been used as counter ion.

In an attempt to trap the purported [Fe_6_C(CO)_15_]^2–^ unsaturated cluster, the oxidation of [Fe_6_C(CO)_15_]^4–^ has been investigated in the presence of phosphine ligands (Scheme 2).

The oxidation of [Fe_6_C(CO)_15_]^4–^ with Me_3_NO in the presence of PTA (PTA = 1,3,5-triaza-7-phosphaadamantane) resulted in the new cluster [Fe_6_C(CO)_15_(PTA)]^2–^. Conversely, when PPh_3_ was used, partial degradation of the Fe_6_C cage of the cluster was observed, resulting in pentanuclear species [Fe_5_C(CO)_13_(PPh_3_)]^2–^. Both reactions required a phosphine: [Fe_6_C(CO)_15_]^4–^ stoichiometric ratio of *ca.* 3:1, in view of the difficulties of adding phosphine ligands to anionic clusters.^12^ The different outcomes of the two reactions might be ascribed to a combination of the different steric, electronic, and σ/π bonding properties of PTA and PPh_3_.

Fe-carbide carbonyl clusters containing a phosphine ligand previously reported on the literature were neutral Fe_5_C(CO)_12_(PPhMe_2_)_3_,^52^ Fe_5_C(CO)_14_(PPh_3_),^39^ Fe_4_C(CO)_10_(κ_3_-Triphos) (Triphos = CH_3_C(CH_2_PPh_2_)_3_),^39^ Fe_5_C(CO)_14_(κ_1_-Triphos),^39^ and the monoanion [HFe_5_C(CO)_13_(PPh_3_)]^−^.^39^ Thus, [Fe_6_C(CO)_15_(PTA)]^2–^ represents the first hexanuclear iron carbide carbonyl cluster containing a phosphine ligand containing an intact Fe_6_C core.

Formation of [Fe_6_C(CO)_15_(PTA)]^2–^ may be viewed as a two-electron oxidation of [Fe_6_C(CO)_15_]^4–^ operated by Me_3_NO (see Section 2.1 for details on this oxidation process) with concomitant coordination of PTA to unsaturated [Fe_6_C(CO)_15_]^2–^. Oxidative degradation of the Fe_6_C cage to Fe_5_C occurs during the synthesis of [Fe_5_C(CO)_13_(PPh_3_)]^2–^.

The new clusters [Fe_6_C(CO)_15_(PTA)]^2–^ and [Fe_5_C(CO)_13_(PPh_3_)]^2–^ have been characterized by IR, ^1^H, ^13^C{^1^H} and ^31^P{^1^H} NMR spectroscopy (see Section 4 and Figures S7, S8, and S13–S18 in the Supporting Information), and their molecular structures determined by SC-XRD as [NEt_4_]_2_[Fe_6_C(CO)_15_(PTA)] and [NEt_4_]_2_[Fe_5_C(CO)_13_(PPh_3_)] salts.

The ^31^P{^1^H} NMR spectrum of [NEt_4_]_2_[Fe_6_C(CO)_15_(PTA)] recorded in CD_3_CN solution displays a singlet at δ_P_ = −23.7 ppm attributable to the unique PTA ligand; by comparison, the free PTA ligand resonates at δ_P_ = −102.0 ppm. The μ_6_-C carbide appears as a doublet at δ_C_ = 485.4 (*J*_C–P_ = 6.0 Hz) in the ^13^C{^1^H} NMR spectrum due to weak coupling to the PTA ligand. A single resonance is also observed in the ^31^P{^1^H} NMR spectrum of [NEt_4_]_2_[Fe_5_C(CO)_13_(PPh_3_)] at δ_P_ = 69.7 ppm, whereas the μ_5_-C carbide ligand resonates at δ_C_ = 478.4 ppm. In this case, the coupling to PPh_3_ is probably too small to be resolved. Indeed, ^2^J_PC_ coupling constants are expected to be greater when the bond angle is close to 180° and to be very small when the angle is close to 90°. The P–Fe–C_carbide_ angle is 108.18(10)° in the case of [NEt_4_]_2_[Fe_5_C(CO)_13_(PPh_3_)], and 131.1(7) and 134.8(7) for the two independent molecules of [NEt_4_]_2_[Fe_6_C(CO)_15_(PTA)], supporting a greater ^2^J_PC_ in the latter case.

**Figure 2:**
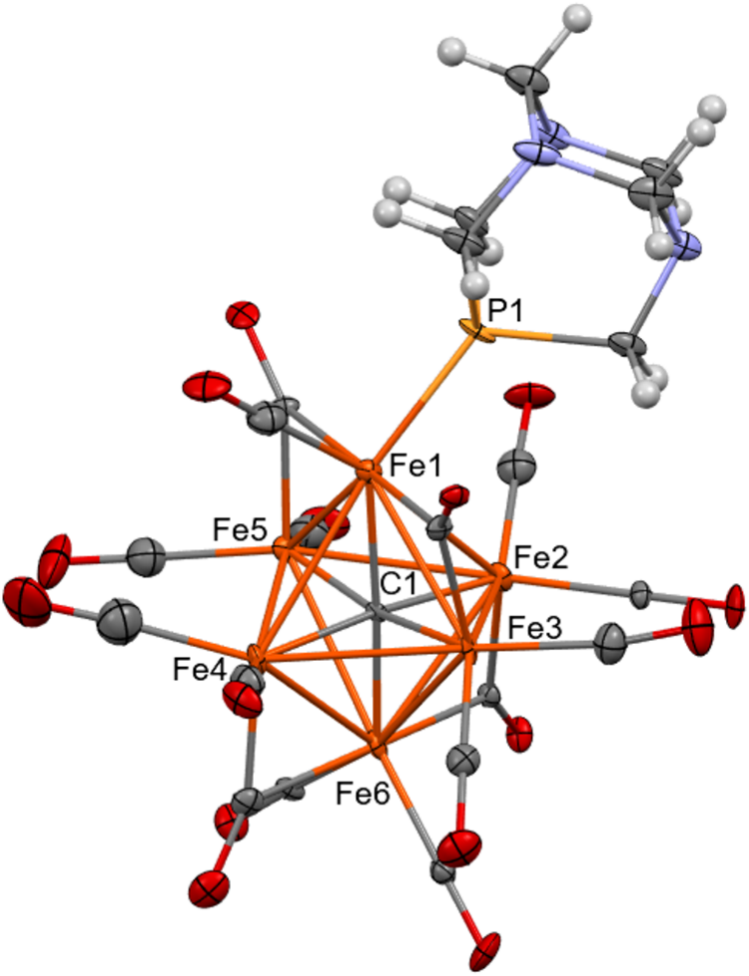
Molecular structure of [Fe_6_C(CO)_15_(PTA)]^2–^ as found in [NEt_4_]_2_[Fe_6_C(CO)_15_(PTA)] (orange, Fe; yellow, P; blue, N; red, O; gray, C; white, H). Thermal ellipsoids are at the 30% probability level.

The molecular structure of [Fe_6_C(CO)_15_(PTA)]^2–^ (Figure 2 and Table S1 in the Supporting Information) is composed of a Fe_6_C octahedral core bonded to 11 terminal and 4 edge bridging carbonyls and one terminal PTA ligand. The Fe–Fe bonding contacts of [Fe_6_C(CO)_15_(PTA)]^2–^ are more spread compared to [Fe_6_C(CO)_16_]^2–^, in view of the replacement of one CO with one PTA ligand. Indeed, the longest Fe–Fe bonding distances [2.854(5) and 2.914(5) Å for the two independent molecules within the unit cell] involve the Fe atom bonded to PTA.

**Figure 3:**
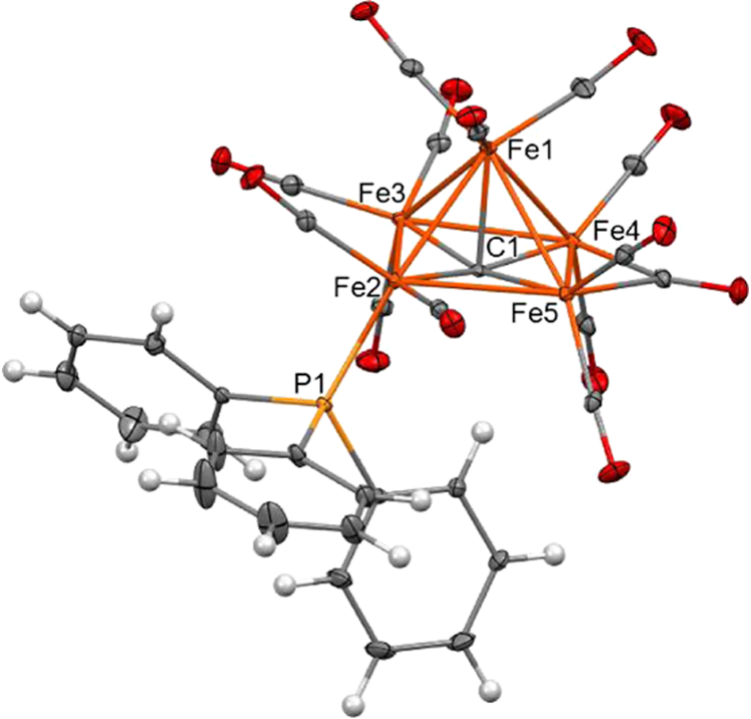
Molecular structure of [Fe_5_C(CO)_13_(PPh_3_)]^2–^ as found in [NEt_4_]_2_[Fe_5_C(CO)_13_(PPh_3_)] (orange, Fe; yellow, P; red, O; gray, C; white, H). Thermal ellipsoids are at the 30% probability level.

The molecular structure of [Fe_5_C(CO)_13_(PPh_3_)]^2–^ (Figure 3 and Table S4 in the Supporting Information) formally derives from that of [Fe_5_C(CO)_14_]^2–^ upon replacement of one terminal CO with PPh_3_ on the square base of the Fe_5_C pyramid, and is closely related to its conjugate hydride monoanion [HFe_5_C(CO)_13_(PPh_3_)]^−^, recently described.^39^ The cluster contains one edge bridging and 12 terminal carbonyls.

The different reactivities exhibited by the two phosphines prompted us to simulate the structure of the hypothetical [Fe_6_C(CO)_15_(PPh_3_)]^2–^ cluster. The comparison of its {Fe_6_C(CO)_15_P} fragment with that of the corresponding DFT-optimized PTA derivative showed scarce differences, as observable in Figure S34 in the Supporting Information. The average bond lengths between the iron center involved in the Fe–P bonds and the surrounding Fe atoms are 2.693 Å in [Fe_6_C(CO)_15_(PPh_3_)]^2–^ and 2.679 Å in [Fe_6_C(CO)_15_(PTA)]^2–^. The distance between P-bonded Fe and the central carbide is 1.891 Å in [Fe_6_C(CO)_15_(PPh_3_)]^2–^ and 1.886 Å in [Fe_6_C(CO)_15_(PTA)]^2–^. No evident structural instability is thus present in the hexanuclear cluster having the formula [Fe_6_C(CO)_15_(PPh_3_)]^2–^. To shed more light, the structure of the hypothetical pentanuclear cluster [Fe_5_C(CO)_13_(PTA)]^2–^ was also optimized and the Gibbs energy variations for the reactions [Fe_6_C(CO)_15_(PPh_3_)]^2–^ → [Fe_5_C(CO)_13_(PPh_3_)]^2–^ + {Fe(CO)_2_} (Δ*G*^PPh_3_^) and [Fe_6_C(CO)_15_(PTA)]^2–^ → [Fe_5_C(CO)_13_(PTA)]^2–^ + {Fe(CO)_2_} (Δ*G*^PTA^) were compared according to the equation: ΔΔ*G* = Δ*G*^PPh_3_^ – Δ*G*^PTA^. The negative value of ΔΔ*G*, equal to −3.9 kcal mol^–1^, indicates that the formation of the pentanuclear cluster is thermodynamically more favored in the case of PPh_3_ as an ancillary ligand.

### 2.3 Oxidation of [Fe_6_C(CO)_15_]^4–^ with Alkylating and Acylating Agents: Synthesis and Molecular Structure of [Fe_5_C(CO)_13_(COMe)]^3–^

**Scheme 3:**
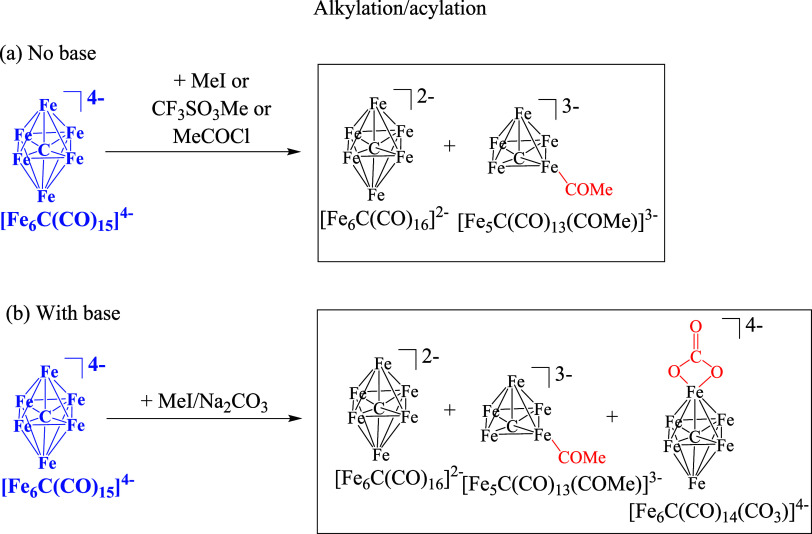
Reactions of [Fe_6_C(CO)_15_]^4–^ in MeCN with Alkylating or Acylating Reactants, (a) without and (b) with a Base All of the species have been structurally characterized by SC-XRD. CO ligands have been omitted for clarity. Both [NEt_4_]^+^ and [NMe_3_CH_2_Ph]^+^ can be used as counterions resulting in similar reactions and separation procedures.

CF_3_SO_3_Me and MeI were investigated as further oxidizing/alkylating agents toward [Fe_6_C(CO)_15_]^4–^, both in the absence and in the presence of a base, such as Na_2_CO_3_ or NaOH (Scheme 3).

The reaction of [Fe_6_C(CO)_15_]^4–^ with MeI (or CF_3_SO_3_Me) without any base results in mixtures of [Fe_6_C(CO)_16_]^2–^ and [Fe_5_C(CO)_13_(COMe)]^3–^, which can be separated since, as [NEt_4_]^+^ or [NMe_3_CH_2_Ph]^+^ salts, the former is soluble in THF and the latter in MeCN. Performing the same reaction in the presence of Na_2_CO_3_, a third compound besides [Fe_6_C(CO)_16_]^2–^ and [Fe_5_C(CO)_13_(COMe)]^3–^ is obtained, that is, [Fe_6_C(CO)_14_(CO_3_)]^4–^. Also in this case, the different products can be separated, as [NEt_4_]^+^ or [NMe_3_CH_2_Ph]^+^ salts, by extraction with organic solvents of increasing polarity, that is, [Fe_6_C(CO)_16_]^2–^ with THF, [Fe_6_C(CO)_14_(CO_3_)]^4–^ with acetone, and [Fe_5_C(CO)_13_(COMe)]^3–^ with MeCN. All of the clusters have been characterized by IR spectroscopy (Figures S1–S10 in the Supporting Information), and their structures determined by SC-XRD. It must be remarked that the same species [Fe_5_C(CO)_13_(COMe)]^3–^ can be also obtained by reaction of [Fe_6_C(CO)_15_]^4–^ with MeCOCl (in the absence of base). Also by using MeCOCl, [Fe_5_C(CO)_13_(COMe)]^3–^ is obtained in a mixture with [Fe_6_C(CO)_16_]^2–^, due to partial oxidation.

The new cluster [Fe_5_C(CO)_13_(COMe)]^3–^ displays ν_CO_ bands at 1921(vs) and 1793(w) cm^–1^ for terminal and bridging carbonyls, respectively. The ν_CO_ bands of [Fe_5_C(CO)_13_(COMe)]^3–^ appear at intermediate wavenumbers between those of [Fe_6_C(CO)_16_]^2–^ and [Fe_6_C(CO)_15_]^4–^, because of the intermediate anionic charge. Accordingly, the carbonyl bands computed for [Fe_5_C(CO)_13_(COMe)]^3–^ are red-shifted with respect to the related [Fe_5_C(CO)_14_]^2–^ cluster (see Figure S35 in the Supporting Information), an effect attributable to the more negative global charge and to the formal replacement of a competing π-acceptor with the acetyl ligand. The stretching band of the – COMe group is observed at 1575 cm^–1^ in the solid state FT-IR spectrum. DFT calculations predict the acetyl ν_CO_ stretching at 1595 cm^–1^, a value that is in line with the experimental outcome.

The presence of the −COMe group has been also demonstrated by ^1^H, ^13^C{^1^H}, and ^1^H–^13^C *gs*-HMBC NMR spectroscopy (Figures S19–S21 in the Supporting Information). The methyl group resonates at δ_H_ = 1.29 ppm and δ_C_ = 30.9 ppm in the ^1^H and ^13^C{^1^H} NMR spectra, respectively, whereas the acetyl–*C*OMe carbon resonates at δ_C_ = 272.7 ppm, and the μ_5_-C carbide at δ_C_ = 485.8 ppm.

**Figure 4:**
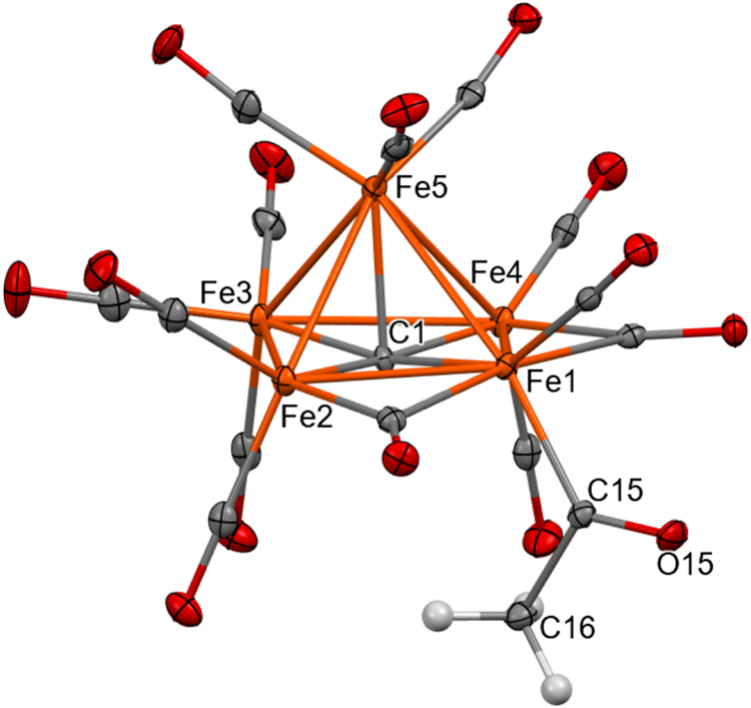
Molecular structure of [Fe_5_C(CO)_13_(COMe)]^3–^ as found in [NMe_3_CH_2_Ph]_3_[Fe_5_C(CO)_13_(COMe)] (orange, Fe; red, O; gray, C; white, H). Thermal ellipsoids are at the 30% probability level.

The structure of [Fe_5_C(CO)_13_(COMe)]^3–^ has been determined on three independent crystals by SC-XRD, that is, [NEt_4_]_3_[Fe_5_C(CO)_13_(COMe)] (two polymorphs, space group *P*1 and *C*2), and [NMe_3_CH_2_Ph]_3_[Fe_5_C(CO)_13_(COMe)]. The molecular structure of the new cluster anion (Figure 4 and Table S4 in the Supporting Information) is closely related to that of [Fe_5_C(CO)_14_]^2–^, being based on the same square-pyramidal Fe_5_C core. The carbide atom is in a semiexposed position, whereas the unique acetyl–COMe group is terminally bonded to one Fe atom of the square base of the cluster. The two edges of the square base adjacent to the Fe-COMe group are bridged by two μ-CO ligands, whereas the remaining 11 carbonyls are in terminal positions. Previous to this work, only three dimeric heteroleptic iron carbonyls were reported containing a terminally bound acetyl group.^53,54^ A few examples of Ru, Os, Co, Rh and Ir carbonyl clusters with terminal −COR ligands were also reported.^55−59^ The most relevant example to this work is probably [Ru_6_C(CO)_15_(COMe)]^−^, which was obtained from the reaction between [Ru_6_C(CO)_16_]^2–^ and MeI.^60^

### 2.4 Electrochemical and Spectroelectrochemical Studies of [Fe_6_C(CO)_15_]^4–^

The electrochemical and spectroelectrochemical behavior of [Fe_6_C(CO)_15_]^4–^ was investigated in MeCN/[N*^n^*Bu_4_][PF_6_], solvent that, in the case of the [Ru_6_C(CO)_15_]^4–^,^40^ proved itself necessary to obtain, by electrochemical or chemical oxidation under Ar atmosphere, the new species [Ru_6_C(CO)_15_(MeCN)]^2–^. The electrochemistry of [Fe_6_C(CO)_16_]^2–^ was also reinvestigated in the same coordinating solvent, and new IR-SEC measurements were carried out.

**Figure 5:**
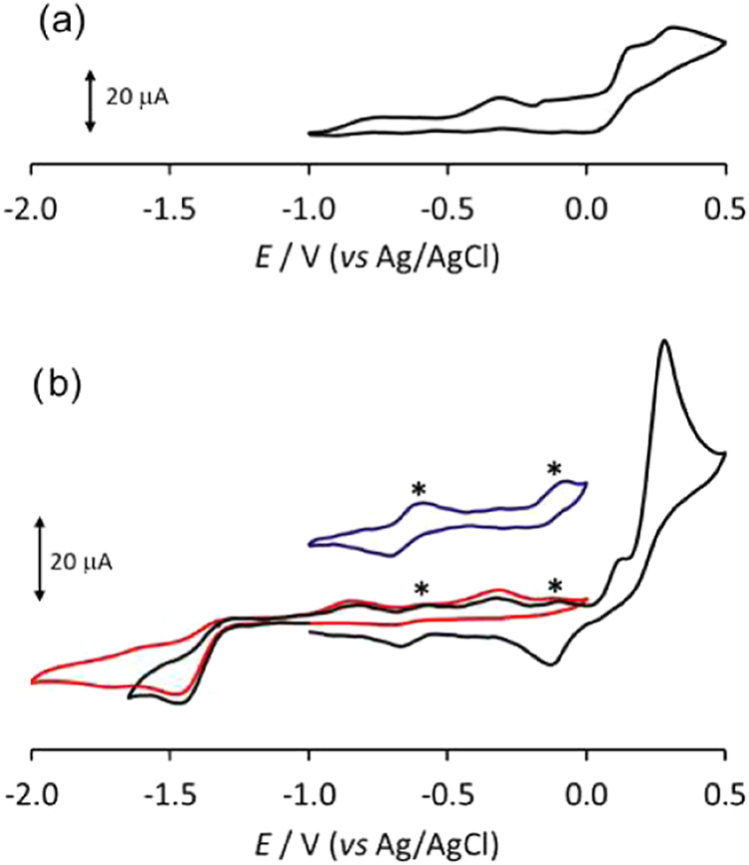
CV response at a Pt electrode in MeCN solution of (a) [Fe_6_C(CO)_15_]^4–^ between −1.0 and +0.5 V; scan rate: 0.1 V s^–1^; (b) [Fe_6_C(CO)_16_]^2–^ between −2.0 and 0.0 V, red line; between −1.65 and +0.5 V, black line; scan rate: 0.1 V s^–1^. Starred peaks are due to impurities. Inset: in blue, CV between −1.0 and 0.0 V (ordinate scale magnified). [N*^n^*Bu_4_][PF_6_] (0.1 mol dm^–3^) supporting electrolyte.

The CV profile of [Fe_6_C(CO)_15_]^4–^ in MeCN/[N*^n^*Bu_4_][PF_6_] solution between −1.0 and +0.5 V features oxidations at −0.32, +0.16, and +0.31 V (Figure 5a), irreversible also at higher scan rates.

**Figure 6:**
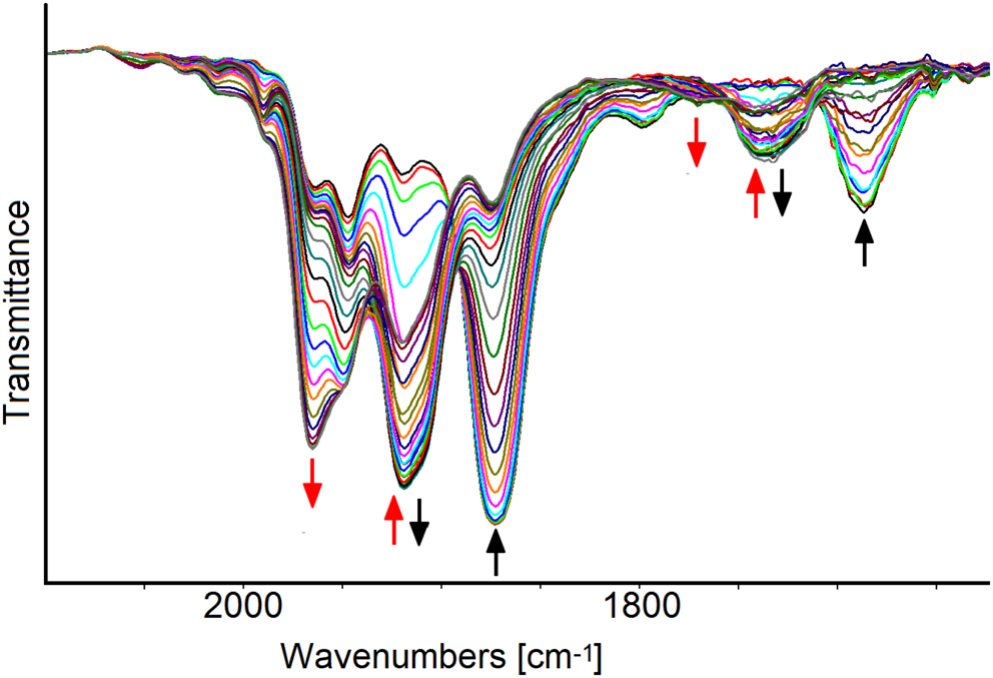
IR spectra of a MeCN solution of [Fe_6_C(CO)_15_]^4–^ recorded in an OTTLE cell during the progressive increase of the potential from −0.2 to +1.0 V (vs Ag pseudo reference electrode, scan rate 1 mV s^–1^). [N*^n^*Bu_4_][PF_6_] (0.1 mol dm^–3^) was used as the supporting electrolyte. The absorptions of the solvent and supporting electrolyte have been subtracted. The two colors (red and black) of the arrows indicate which bands change simultaneously.

When the oxidation processes of [Fe_6_C(CO)_15_]^4–^ were investigated by *in situ* IR-SEC in an optically transparent thin-layer electrochemical (OTTLE) cell,^61^ the sequence of IR spectra recorded between −0.2 and +1.1 V showed two subsequent shifts of the terminal and bridging CO bands at higher wavenumbers pointing out two consecutive oxidations of [Fe_6_C(CO)_15_]^4–^ (Figure 6); in the potential range from −0.2 to +0.5 V, the initial absorptions of [Fe_6_C(CO)_15_]^4–^ (1873 and 1687 cm^–1^, Figure S22 in the Supporting Information, black line) were substituted by two bands at 1919 and 1735 cm^–1^ (Figure S22 in the Supporting Information, red and blue line), due to a new species, presumably with −3 charge, and, unexpectedly, when the working electrode (WE) potential was further increased up to +1.0 V, the IR bands of [Fe_6_C(CO)_16_]^2–^ at 1967 and 1773 cm^–1^ were observed in the solution as the most intense absorptions (Figure S22 in the Supporting Information, green line). The second oxidation was complete at the potential of +1.1 V and the intense ν_CO_ band at 1967 cm^–1^ due to [Fe_6_C(CO)_16_]^2–^ (Figure S23 in the Supporting Information) was accompanied by three weak bands at 2113, 2054, and 2008 cm^–1^, that we observed also during the [Fe_6_C(CO)_16_]^2–^ oxidation (see below). Presumably, decomposition reactions of the Fe_6_C core released the CO necessary for the formation of [Fe_6_C(CO)_16_]^2–^. Nevertheless, the IR spectrum of the solution, at the end of the back-reduction scan (Figure S24 in the Supporting Information), showed that the tetra-anion had reformed in high yields (Figure S25 in the Supporting Information). It must be highlighted that in the IR spectral sequence of Figure S24 in the Supporting Information, that is, the back-reduction scan, the 1919 cm^–1^ band is barely visible.

The CV of [Fe_6_C(CO)_16_]^2–^ in MeCN solution (Figure 5b) confirmed the previous results in dichloroethane, that is, an irreversible two-electron reduction (*E*_p_ = −1.47 V), whose products are oxidized at −0.82 and −0.32 V, and irreversible oxidation processes.^62^ However, when we examined the CV profiles between 0.0 and −1.65 V at increasing scan rates from 0.1 to 4.0 V/s, we observed the increase of a weak back-reoxidation peak (Figure S26 in the Supporting Information) and the decrease of the two peaks due to the decomposition products. The first oxidation at +0.13 V, in the same range of scan rates, remained irreversible. The process at −0.32 V, observable in the back-scan after the irreversible reduction of [Fe_6_C(CO)_16_]^2–^, can be confidently attributed to the oxidation of [Fe_6_C(CO)_15_]^4–^ formed by the reduction/decabonylation of [Fe_6_C(CO)_16_]^2–^.

**Figure 7:**
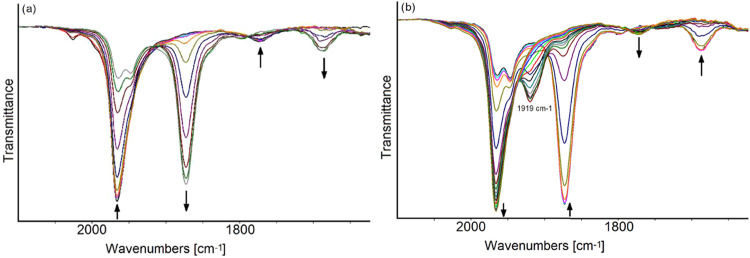
IR spectra of a MeCN solution of [Fe_6_C(CO)_16_]^2–^ recorded in an OTTLE cell during (a) the progressive decrease of the potential from −0.6 to −1.2 V (vs Ag pseudo reference electrode, scan rate 1 mV s^–1^) and (b) the oxidation back-scan from −1.2 to 0.0 V (vs Ag pseudo reference electrode). [N*^n^*Bu_4_][PF_6_] (0.1 mol dm^–3^) as the supporting electrolyte. The absorptions of the solvent and supporting electrolyte have been subtracted.

When the reduction of [Fe_6_C(CO)_16_]^2–^ was investigated by IR-SEC, during the progressive decrease of the WE potential from −0.6 to −1.2 V (vs Ag pseudo reference electrode, scan rate 1 mV s^–1^), a red shift of the terminal and bridging ν_CO_ bands of [Fe_6_C(CO)_16_]^2–^ from 1967 and 1773 to 1873 and 1687 cm^–1^ was observed, indicating the quantitative formation of the decarbonylated tetra-anion [Fe_6_C(CO)_15_]^4–^ (Figure 7a). An IR spectrum superimposable to the initial one was obtained after the oxidation back-scan from −1.2 up to 0.0 V (Figure S27 in the Supporting Information); moreover, an intermediate, medium intensity, IR absorption at 1919 cm^–1^ was observed in the IR spectra recorded during the back-oxidation between −0.6 and −0.2 V (Figure 7b) and was attributed the intermediate cluster with −3 charge.

We also performed the oxidation of [Fe_6_C(CO)_16_]^2–^ in the OTTLE cell. When the WE potential was increased from +0.2 to +0.6 V, the IR absorptions of the dianion were replaced by bands at higher wavenumbers (2113(w), 2054(s), 2008–1990(br) cm^–1^) (Figure S28a in the Supporting Information), and the starting compound was almost completely reobtained in the potential back-scan (Figure S28c in the Supporting Information). Moreover, we observed that a fast decrease of the 2113 and 2054 cm^–1^ bands corresponded to an increased intensity of bands between 2009 and 1999 cm^–1^ that slowly restored the 1967 cm^–1^ absorption of [Fe_6_C(CO)_16_]^2–^ when the WE potential was lowered to −0.6 V (Figure S28b in the Supporting Information). We were not able to unequivocally assign the observed ν_CO_ bands to oxidized species, on the other hand, based on the results of the IR-SEC experiments reported in this paper, we cannot exclude the formation of the oxidized anionic [Fe_6_C(CO)_16/15_]^−^ and neutral Fe_6_C(CO)_17_ clusters, since the shift of the terminal CO stretching band (87 cm^–1^, from 1967 to 2054 cm^–1^) is similar to that observed in the above-reported two-electron reduction/decarbonylation of [Fe_6_C(CO)_16_]^2–^ to give [Fe_6_C(CO)_15_]^4–^ (94 cm^–1^). The formation of the 86 cluster valence electrons (CVE) Fe_6_C(CO)_17_ recently isolated and crystallographically characterized,^39^ seems more plausible to us that reversible redox fragmentation to lower nuclearity species followed by a back redox condensation to the initial cluster in spite of the lack of free CO.

As in the case of the previously reported [Ru_6_C(CO)_16_]^2–^/[Ru_6_C(CO)_15_]^4–^ clusters,^40^ the electron transfers are followed by relatively fast decarbonylation/carbonylation reactions, due to the stability of the 86 CVE species, that make the processes chemically irreversible in the CV time scale at the lowest scan rates. However, when the same electron transfers are studied by *in situ* IR-SEC in an OTTLE cell, a microelectrolysis reactor without free volume, the CO dissociated in the reduction remains available for the back-oxidation step, and the reaction can be reversed almost completely. In the same way, the geometry of the reactor affected also the oxidation processes where the two consecutive one-electron removals are accompanied by uptake of CO arising from traces of decomposition reactions.

**Scheme 4:**
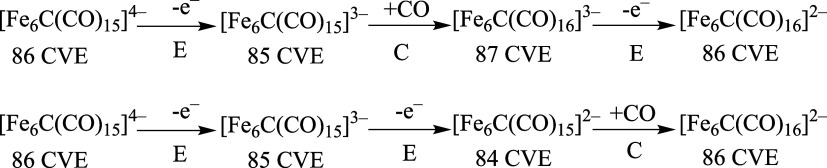
Proposed Mechanisms for the Electrochemical Oxidation of [Fe_6_C(CO)_15_]^4–^

The IR-SEC experiments onto the Fe_6_C clusters have demonstrated that the transformation (1) in the OTTLE cell is almost quantitative in both directions, and surprisingly reversible also under Ar atmosphere, the missing CO presumably being effectively recovered by decomposition reactions of [Fe_6_C(CO)_15_]^4–^ or of present impurities (starred bands at 1964 and 1947 cm^–1^ in the black spectrum of Figure S22 in the Supporting Information).
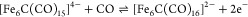
1During the oxidation of the tetra-anion, an intermediate species accumulates (Figure 6) suggesting two consecutive electron removals: an EEC or ECE mechanism can be supposed for this transformation (Scheme 4), the carbonylation reaction under an argon atmosphere being the slow step that enables the accumulation of [Fe_6_C(CO)_15_]^3–^.

On the other hand, during the reduction of [Fe_6_C(CO)_16_]^2–^ (Figure 7a), the formation of [Fe_6_C(CO)_15_]^4–^ was observed right from the beginning, without ever seeing IR bands attributable to intermediate species, as if two electrons were consecutively acquired and one CO ligand dissociated according to a EEC mechanism (for complete discussion of the reaction mechanism see Section 2.5).

Moreover, during the reverse back-oxidation step, the intermediate cluster with −3 charge (ν_CO_ 1919 cm^–1^) accumulated in a minor extent (Figure 7b) with respect to the direct oxidation of [Fe_6_C(CO)_15_]^4–^, the only apparent difference being the presence of dissolved CO arising from the forward reduction step of [Fe_6_C(CO)_16_]^2–^. These findings seem to indicate that the interconversion of [Fe_6_C(CO)_16_]^2–^/[Fe_6_C(CO)_15_]^4–^ is the result of two one-electron steps and that the intermediate – 3 charge cluster can accumulate more or less during the IR-SEC experiments, depending on the experimental conditions as scan rate, dissolved CO, and presence of impurities.

The quantitative formation of [Fe_6_C(CO)_15_]^4–^ by the [Fe_6_C(CO)_16_]^2–^ reduction was achieved in an OTTLE cell under an Ar atmosphere, with the evolved CO remaining available for the following back-oxidation step to the starting cluster. In the case of [Ru_6_C(CO)_16_]^2–^ the reduced cluster was observed only in mixture with unidentified products, probably arising from reactions due to evolved CO. Under an Ar atmosphere, for the complete two-electron oxidation of [Ru_6_C(CO)_15_]^4–^ was essential the use of MeCN coordinating solvent to obtain the 86 CVE species [Ru_6_C(CO)_15_(MeCN)]^2–^. On the other hand, despite, or perhaps thanks, to the lower stability of Fe clusters in comparison to those of Ru, the oxidation of [Fe_6_C(CO)_15_]^4–^ allowed the sequential and almost quantitative observation of the −3 and −2 charged clusters, whose formation was made possible by CO released by decomposition reactions that did not lower to much the yield in the subsequent back-reduction.

Concerning the [Fe_6_C(CO)_16_]^2–^ formation from [Fe_6_C(CO)_15_]^4–^, an EEC mechanism can be supposed, with 85–84 CVE species as intermediates (Scheme 4).

Lastly, while the neutral stable cluster Ru_6_C(CO)_17_ had been long known, the IR-SEC studies here reported provided evidence of the existence of the analogous Fe_6_C(CO)_17_, whose isolation and structural characterization have been reported, only recently and independently, by Rose et al.^39^

### 2.5 Computational Studies on the Redox Intermediates

The clusters potentially involved in the redox processes starting from [Fe_6_C(CO)_16_]^2–^ and [Fe_6_C(CO)_15_]^4–^ were computationally simulated by means of CPCM/r^2^SCAN-3c calculations. In all cases, the DFT-optimized structures showed good agreement with the X-ray outcomes. The one-electron reduction of [Fe_6_C(CO)_16_]^2–^ should afford [Fe_6_C(CO)_16_]^3–^ as first intermediate. Such a species could follow two different pathways: the elimination of a carbonyl ligand, with the formation of [Fe_6_C(CO)_15_]^3–^, or the addition of a second electron leading to [Fe_6_C(CO)_16_]^4–^. In the first case, the final [Fe_6_C(CO)_15_]^4–^ cluster is obtained by subsequent addition of an electron (ECE mechanism), while in the second case through elimination of a CO from [Fe_6_C(CO)_16_]^4–^ (EEC mechanism).

From a thermodynamic point of view, the dissociation of a CO ligand from [Fe_6_C(CO)_16_]^3–^ appears thermodynamically unfavorable: [Fe_6_C(CO)_16_]^3–^ → CO + [Fe_6_C(CO)_15_]^3–^, Δ*G* = +11.8 kcal mol^–1^. Such an outcome is more consistent with an EEC mechanism. The elimination of CO is characterized by negative Gibbs energy variation after the second reduction: [Fe_6_C(CO)_16_]^4–^ → CO + [Fe_6_C(CO)_15_]^4–^, Δ*G* = −23.9 kcal mol^–1^.

Focusing the attention on the reverse process, i.e., the oxidation of [Fe_6_C(CO)_15_]^4–^, the removal of the first electron should afford [Fe_6_C(CO)_15_]^3–^. The coordination of a CO ligand leading to [Fe_6_C(CO)_16_]^3–^ is clearly favorable, being the reverse of the reaction previously described reaction. It is however worth noting that the concentration of free CO under the experimental conditions should be very low, being the decomposition of some cluster molecules the only possible source. If we suppose that the −3 species observed is [Fe_6_C(CO)_15_]^3–^, then its oxidation should require higher potential with respect to the parent anion [Fe_6_C(CO)_15_]^4–^. Accordingly, the HOMO energy of [Fe_6_C(CO)_15_]^3–^ is about 0.66 eV lower than that of [Fe_6_C(CO)_15_]^4–^. The −3 species that accumulates during the oxidation process is, therefore, most likely [Fe_6_C(CO)_15_]^3–^, and the oxidation pathway should follow an EEC mechanism. As a further confirmation, the HOMO energy value of [Fe_6_C(CO)_16_]^3–^ is about 0.87 eV higher than that of [Fe_6_C(CO)_15_]^4–^, thus, it is unlikely that this cluster could accumulate during the oxidation process. The DFT-optimized structures of the clusters here described are collected in Figure S36 in the Supporting Information together with the simulated IR spectra. Unfortunately, the simulated terminal carbonyl regions of [Fe_6_C(CO)_15_]^3–^ and [Fe_6_C(CO)_16_]^3–^ are quite similar; thus, the assignment of the observed intermediate from the experimental IR spectra appears difficult.

It is worth noting that the change of global charge poorly affects the {Fe_6_C} cage, and the carbonyl ligands maintain their coordination mode. The root-mean-square deviation (RMSD) values for selected couples are collected in Table S6 in the Supporting Information.

The second oxidation of the proposed EEC mechanism should afford the unsaturated [Fe_6_C(CO)_15_]^2–^ anion, as shown in Figure S37 in the Supporting Information. Its structure is roughly similar to that of the parent −3 cluster (Table S6 in the Supporting Information). Some carbonyl ligands change their coordination modes upon oxidation. On considering the experimental conditions, the formation of the acetonitrile derivative [Fe_6_C(CO)_15_(MeCN)]^2–^ is also possible. The presence of small amounts of free CO derived from the decomposition of some cluster molecules leads to the final product according to the reactions [Fe_6_C(CO)_15_]^2–^ + CO → [Fe_6_C(CO)_16_]^2–^, Δ*G* = −38.4 kcal mol^–1^, or [Fe_6_C(CO)_15_(MeCN)]^2–^ + CO → [Fe_6_C(CO)_16_]^2–^ + MeCN, Δ*G* = −25.4 kcal mol^–1^. Differently from the Ru analogue, [Fe_6_C(CO)_15_(MeCN)]^2–^ was not detected probably because of the lower stability of the Fe clusters and the consequent relatively higher concentration of available CO. The Gibbs energy variation for the CO/MeCN exchange is in fact similar to that calculated for the corresponding Ru species at CPCM/PBEh-3c level, −21.8 kcal mol^–1^.^40^ The DFT-optimized structure of [Fe_6_C(CO)_15_(MeCN)]^2–^ is shown in Figure S37 in the Supporting Information.

The oxidation of [Fe_6_C(CO)_16_]^2–^ afforded the recently reported Fe_6_C(CO)_17_ neutral cluster.^39^ For completeness, this species was also computationally investigated starting from the published X-ray data. The carbonyl region of the simulated IR spectrum is noticeably blue-shifted (about 77 cm^–1^ for what concerns the terminal ν_CO_ stretchings) with respect to the parent [Fe_6_C(CO)_16_]^2–^, as observable in Figure S38 in the Supporting Information. The computational outcome is in good agreement with the data obtained from the spectroelectrochemical measurements.

## 3 Conclusions

Four new heteroleptic iron carbide carbonyl clusters have been isolated and fully characterized: that is, [Fe_6_C(CO)_14_(CO_3_)]^4–^, [Fe_6_C(CO)_15_(PTA)]^2–^, [Fe_5_C(CO)_13_(PPh_3_)]^2–^, and [Fe_5_C(CO)_13_(COMe)]^3–^. Their formation is accompanied by traces of previously known [Fe_6_C(CO)_16_]^2–^. All of these species have been obtained upon oxidation of [Fe_6_C(CO)_15_]^4–^ under different experimental conditions. Formally, their formation may be viewed as a two-electron oxidation of [Fe_6_C(CO)_15_]^4–^ followed by the addition of a further ligand to the resulting unsaturated cluster. In particular, addition of CO (always present due to partial degradation of the clusters) affords [Fe_6_C(CO)_16_]^2–^, addition of CO_3_^2–^ (formed *in situ* by the Hieber reaction promoted by bases such as Na_2_CO_3_ and NaOH) affords [Fe_6_C(CO)_14_(CO_3_)]^4–^, and addition of PTA affords [Fe_6_C(CO)_15_(PTA)]^2–^. In all of these cases, the Fe_6_C cage of the parent cluster is retained. Conversely, when the oxidation is carried out in the presence of PPh_3_, one Fe atom is removed, resulting in pentanuclear species [Fe_5_C(CO)_13_(PPh_3_)]^2–^. For these syntheses, [Cp_2_Fe][PF_6_], [C_7_H_7_][BF_4_], and Me_3_NO, can be used as oxidizing agents. In contrast, when MeI, CF_3_SO_3_Me or MeCOCl are used, [Fe_5_C(CO)_13_(COMe)]^3–^ is obtained, as the result of oxidation and addition of Me- or MeCO-groups and removal of one Fe atom. These results clearly indicate the synthetic potentiality of the chemical oxidation of [Fe_6_C(CO)_15_]^4–^, which may lead to different Fe_6_C and Fe_5_C heteroleptic species depending on the reaction conditions.

Electrochemical and spectroelectrochemical studies, supported by computational investigations, point out that oxidation of [Fe_6_C(CO)_15_]^4–^ proceeds *via* an EEC mechanism. The [Fe_6_C(CO)_15_]^3–^ intermediate is sufficiently stable to accumulate during IR-SEC also thanks to the relative stability of its HOMO, and it has been spectroscopically identified. It is worth noting that DFT calculations support an EEC mechanism also for the electrochemical reduction of [Fe_6_C(CO)_16_]^2–^ to [Fe_6_C(CO)_15_]^4–^, given the unfavorable dissociation of CO from [Fe_6_C(CO)_16_]^3–^.

The fact that two electrons may be electrochemically removed in sequence from [Fe_6_C(CO)_15_]^4–^, and the process may be almost quantitatively reversed in the IR-SEC time scale, indicates that its overall two-electron oxidation does not immediately lead to degradation of its Fe_6_C cage. This is in keeping with the isolation of heteroleptic Fe_6_C clusters when chemical oxidation is carried out in the presence of suitable ligands. At the same time, this suggests that degradation to Fe_5_C species mainly depends on the nature of the ligands present in the reaction medium. In this sense, coordination of carbonate to [Fe_6_C(CO)_14_(CO_3_)]^4–^ gives insight into the “decapitation” of the Fe_6_C parent cluster. Indeed, degradation of Fe_6_C to Fe_5_C carbonyl clusters is usually reported as an oxidative process involving elimination of Fe^2+^ ions.^3,29^ Thus, the Fe atoms chelated by the carbonate of [Fe_6_C(CO)_14_(CO_3_)]^4–^ may be viewed as an incipient oxidized FeCO_3_ group, ready to “leave” the cluster.

The hexanuclear carbide cluster [Fe_6_C(CO)_16_]^2–^ has been viewed for a long time as an inert species reluctant to CO substitution. We have previously shown that Lewis acids can be added to [Fe_6_C(CO)_16_]^2–^, upon its reduction to [Fe_6_C(CO)_15_]^4–^.^5^ Very recently, Rose et al. have demonstrated the possibility of adding Lewis bases to [Fe_6_C(CO)_16_]^2–^ upon its oxidation *via* an unsaturated [Fe_6_C(CO)_16_] neutral cluster.^39^ Herein, we have developed an alternative oxidative process for the addition of Lewis bases, which involves the reduced [Fe_6_C(CO)_15_]^4–^ cluster. This procedure is rather versatile and can be applied both to soft Lewis bases, such as CO and phosphines, and harder Lewis bases, such as CO_3_^2–^. Even if sometimes partial fragmentation of the Fe_6_C cage is observed, it also allows the isolation of intact hexa-iron carbide clusters. Overall, the addition of Lewis bases enabled by oxidation of [Fe_6_C(CO)_15_]^4–^ may be viewed as an alternative way to tackle the carbide problem in nitrogenase-related cluster chemistry, eventually allowing the introduction of organic and/or inorganic sulfur into an intact Fe_6_C cage. More in general, the synthetic approach herein described might be used for the obtainment of Fe_6_C and Fe_5_C carbide carbonyl clusters differently functionalized for applications in catalysis and electrocatalysis.^23,24,37,38^

## 4 Experimental Section

### 4.1 General Procedures

All reactions and sample manipulations were carried out using standard Schlenk techniques under nitrogen and in dried solvents. All of the reagents were commercial products (Aldrich) of the highest purity available and used as received, except [NEt_4_]_4_[Fe_6_C(CO)_15_] and [NMe_3_CH_2_Ph]_4_[Fe_6_C(CO)_15_] which have been prepared according to the literature.^5^ Analyses of C, H, and N were obtained with a Thermo Quest Flash EA 1112NC instrument. IR spectra were recorded on a PerkinElmer Spectrum One interferometer in CaF_2_ cells. ATR-IR spectra were recorded on a PerkinElmer Spectrum Two interferometer. ^1^H, ^13^C{^1^H}, ^31^P{^1^H} and ^1^H–^13^C *gs*-HMBC NMR measurements were performed on Varian Mercury Plus 400 MHz and Bruker Avance III 600 MHz instruments. The proton and carbon chemical shifts were referenced to the nondeuterated aliquot of the solvent. The phosphorus chemical shifts were referenced to external H_3_PO_4_ (85% in D_2_O). Reaction yields have been calculated based on the total amount of Fe in the isolated products and starting materials. Structure drawings have been performed with Mercury.^63^

Caution! Extreme care should be taken in both the handling of the cryogen liquid nitrogen and its use in the Schlenk line trap to avoid the condensation of oxygen from air.

Caution! CO may be generated during manipulation of these compounds. All of the operations must be carried out under a well-ventilated fume hood.

### 4.2 Reaction of [NEt_4_]_4_[Fe_6_C(CO)_15_] with Me_3_NO and Na_2_CO_3_: Synthesis of [NEt_4_]_3_[H_3_O][Fe_6_C(CO)_14_(CO_3_)]

Me_3_NO·2H_2_O (90 mg, 0.810 mmol) was added in two portions over a period of 3 h to a solution of [NEt_4_]_4_[Fe_6_C(CO)_15_] (0.480 g, 0.373 mmol) in MeCN (20 mL) containing also Na_2_CO_3_ (0.100 g, 0.943 mmol). The resulting solution was stirred overnight at room temperature, and then the solvent was removed under vacuum. The solid residue was washed with H_2_O (3 × 20 mL) and toluene (2 × 20 mL), and extracted with THF (20 mL), acetone (20 mL) and MeCN (20 mL). The residue was dried under vacuum after each extraction. The THF solution contained [NEt_4_]_2_[Fe_6_C(CO)_16_], as evidenced by IR analysis (ν_CO_ 1964(vs) cm^–1^). Slow diffusion of *n*-hexane (40 mL) on the acetone solution afforded crystals of [NEt_4_]_3_[H_3_O][Fe_6_C(CO)_14_(CO_3_)] suitable for SC-XRD analyses (yield 0.13 g, 29% based on Fe). The MeCN solution contained [NEt_4_]_4_[Fe_6_C(CO)_15_], as evidenced by IR analysis (ν_CO_ 1869(vs) cm^–1^).

#### [NEt_4_]_3_[H_3_O][Fe_6_C(CO)_14_(CO_3_)]

C_40_H_63_Fe_6_N_3_O_18_ (1209.03): calcd. C 39.74, H 5.25, N 3.48; found: C 39.97, H 5.41, N 3.61. IR (acetone, 293 K) ν_CO_: 2015(w), 1947(vs), 1785(w) cm^–1^. IR (solid, ATR, 293 K): ν_CO_: 1925(sh), 1900(vs), 1738(m) cm^–1^; ν_CO_3__: 1575–1615(sh) cm^–1^. ^1^H NMR (CD_3_CN, 298 K) δ_H_: 3.20 (br, CH_2_, cation), 1.23 (br, CH_3_, cation) ppm. ^13^C{^1^H} NMR (CD_3_CN, 298 K) δ_C_: 478.4 (Fe_6_*C*), 229.0, 227.2, 223.4, 221.1 (CO), 53.2 (CH_2_, cation), 7.8 (CH_3_, cation).

### 4.3 Reaction of [NEt_4_]_4_[Fe_6_C(CO)_15_] with Me_3_NO and PTA: Synthesis of [NEt_4_]_2_[Fe_6_C(CO)_15_(PTA)]

Me_3_NO·2H_2_O (90 mg, 0.810 mmol) and PTA (180 mg, 1.14 mmol) were added in small portions over a period of 3 h to a solution of [NEt_4_]_4_[Fe_6_C(CO)_15_] (0.480 g, 0.373 mmol) in MeCN (20 mL). After 4 h, the solvent was removed in vacuum and the residue washed with H_2_O (3 × 20 mL) and toluene (2 × 20 mL), and extracted with THF (20 mL). Slow diffusion of *n*-hexane (40 mL) on the THF solution afforded crystals of [NEt_4_]_2_[Fe_6_C(CO)_15_(PTA)] suitable for SC-XRD analyses (yield 0.12 g, 27% based on Fe).

#### [NEt_4_]_2_[Fe_6_C(CO)_15_(PTA)]

C_38_H_52_Fe_6_N_5_O_15_P (1184.91): C 38.52, H 4.42, N 5.91; found: C 38.78, H 4.09, N 6.13. IR (acetone, 293 K) ν_CO_: 2009(w), 1950(vs) cm^–1^. IR (MeCN, 293 K) ν_CO_: 2010(w), 1952(vs), 1748(m) cm^–1^. ^1^H NMR (CD_3_CN, 298 K) δ_H_: 4.44–4.35 (m, CH_2_, PTA), 3.94 (m, CH_2_, PTA), 3.19 (br, CH_2_, cation), 1.24 (br, CH_3_, cation) ppm. ^13^C{^1^H} NMR (CD_3_CN, 298 K) δ_C_: 485.4 (d, *J*_C–P_ = 6.0 Hz, Fe_6_*C*), 233.0 (CO), 73.5 (d, *J*_C–P_ = 6.3 Hz, CH_2_–N, PTA), 57.1 (d, *J*_C–P_ = 13.3 Hz, CH_2_–P, PTA), 53.9 (CH_2_, cation), 8.2 (CH_3_, cation). ^31^P{^1^H} NMR (CD_3_CN, 298 K) δ_P_: −23.7 ppm.

### 4.4 Reaction of [NEt_4_]_4_[Fe_6_C(CO)_15_] with Me_3_NO and PPh_3_: Synthesis of [NEt_4_]_2_[Fe_5_C(CO)_13_(PPh_3_)]

Me_3_NO·2H_2_O (90 mg, 0.810 mmol) and PPh_3_ (0.280 g, 1.07 mmol) were added in small portions over a period of 4 h to a solution of [NEt_4_]_4_[Fe_6_C(CO)_15_] (0.430 g, 0.334 mmol) in MeCN (20 mL). The resulting solution was stirred at room temperature for 3 days, and then the solvent was removed in vacuum. The solid residue was washed with H_2_O (3 × 20 mL), toluene (2 × 20 mL) and THF (20 mL), and extracted with acetone (20 mL). The residue was dried under vacuum after each extraction. Slow diffusion of *n*-hexane (40 mL) on the acetone solution afforded crystals of [NEt_4_]_2_[Fe_5_C(CO)_13_(PPh_3_)] suitable for SC-XRD analyses (yield 0.11 g, 23% based on Fe).

#### [NEt_4_]_2_[Fe_5_C(CO)_13_(PPh_3_)]

C_48_H_55_Fe_5_N_2_O_13_P (1178.16): C 48.93, H 4.71, N 2.38; found: C 48.71, H 5.04, N 2.62. IR (acetone, 293 K) ν_CO_: 2002(w), 1963(s), 1945(vs) cm^–1^. IR (MeCN, 293 K) ν_CO_: 2004(w), 1965(s), 1948(vs), 1745(w) cm^–1^. ^1^H NMR (CD_3_CN, 298 K) δ_H_: 7.41–7.25 (m, Ph), 3.17 (br, CH_2_, cation), 1.21 (br, CH_3_, cation) ppm. ^13^C{^1^H} NMR (CD_3_CN, 298 K) δ_C_: 478.4 (Fe_5_*C*), 225.2, 223.4, 221.2 (CO), 139.5 (d, *J*_C–P_ = 37.0 Hz, Ph), 135.1 (d, *J*_C–P_ = 10.7 Hz, Ph), 129.8 (s, Ph), 128.2 (d, *J*_C–P_ = 8.6 Hz, Ph), 53.1 (CH_2_, cation), 7.8 (CH_3_, cation). ^31^P{^1^H} NMR (CD_3_CN, 298 K) δ_P_: 69.7 ppm.

### 4.5 Reaction of [NEt_4_]_4_[Fe_6_C(CO)_15_] with MeI: Synthesis of [NEt_4_]_3_[Fe_5_C(CO)_13_(COMe)]

A solution of MeI (30 μL, 0.482 mmol) in MeCN (0.5 mL) was added dropwise over a period of 2 h to a solution of [NEt_4_]_4_[Fe_6_C(CO)_15_] (0.370 g, 0.287 mmol) in MeCN (20 mL), and the mixture was stirred at room temperature overnight. Then, the solvent was removed in vacuum and the residue washed with H_2_O (3 × 20 mL) and toluene (2 × 20 mL), and extracted with THF (20 mL), and MeCN (20 mL). The residue was dried under vacuum after each extraction. The THF solution contained [NEt_4_]_2_[Fe_6_C(CO)_16_], as evidenced by IR analysis (ν_CO_ 1964(vs) cm^–1^). Slow diffusion of *n*-hexane (2 mL) and di-isopropyl ether (30 mL) on the MeCN solution afforded crystals of [NEt_4_]_3_[Fe_5_C(CO)_13_(COMe)] (polymorph with space group *P*1) suitable for SC-XRD analyses (yield 0.10 g, 27% based on Fe).

#### [NEt_4_]_3_[Fe_5_C(CO)_13_(COMe)]

C_40_H_63_Fe_5_N_3_O_14_ (1089.18): C 44.11, H 5.83, N 3.86; found: C 44.34, H 6.03, N 3.54. IR (MeCN, 293 K) ν_CO_: 1988(w), 1921(s), 1793(w) cm^–1^. IR (nujol mull, 293 K) ν_CO_: 1983(w), 1897(s), 1884 (sh), 1838 (m), 1729(ms), 1711(m) cm^–1^; ν_COMe_: 1575(m) cm^–1^. ^1^H NMR (CD_3_CN, 298 K) δ_H_: 3.19 (br, CH_2_, cation), 1.29 (s, CO*Me*), 1.22 (br, CH_3_, cation) ppm. ^13^C{^1^H} NMR (CD_3_CN, 298 K) δ_C_: 485.8 (Fe_5_*C*), 272.7 (*C*OMe), 236.7, 227.2, 223.2, 221.1 (CO), 53.1 (CH_2_, cation), 30.9 (CO*Me*), 7.8 (CH_3_, cation).

NOTE: The–COMe resonances were assigned by ^1^H–^13^C *gs*-HMBC NMR spectroscopy.

### 4.6 Reaction of [NMe_3_CH_2_Ph]_4_[Fe_6_C(CO)_15_] with MeI: Synthesis of [NMe_3_CH_2_Ph]_3_[Fe_5_C(CO)_13_(COMe)]

A solution of MeI (20 μL, 0.321 mmol) in MeCN (0.5 mL) was added dropwise over a period of 2 h to a solution of [NMe_3_CH_2_Ph]_4_[Fe_6_C(CO)_15_] (0.390 g, 0.285 mmol) in MeCN (20 mL), and the mixture was stirred at room temperature overnight. Then, the solvent was removed in vacuum and the residue washed with H_2_O (3 × 20 mL) and toluene (2 × 20 mL), and extracted with THF (20 mL), and MeCN (20 mL). The residue was dried under vacuum after each extraction. Slow diffusion of *n*-hexane (40 mL) on the THF solution afforded crystals of [NMe_3_CH_2_Ph]_2_[Fe_6_C(CO)_16_] suitable for SC-XRD analyses (yield 0.091 g, 29% based on Fe). Slow diffusion of *n*-hexane (2 mL) and di-isopropyl ether (40 mL) on the MeCN solution afforded a mixture of crystals of [NMe_3_CH_2_Ph]_4_[Fe_6_C(CO)_15_] (two different polymorphs, both with space group *C*2/*c*) and [NMe_3_CH_2_Ph]_3_[Fe_5_C(CO)_13_(COMe)] suitable for SC-XRD analyses (yield 0.101 g).

#### [NMe_3_CH_2_Ph]_2_[Fe_6_C(CO)_16_]

IR (THF, 293 K) ν_CO_: 1966(s), 1772(w) cm^–1^.

#### [NMe_3_CH_2_Ph]_4_[Fe_6_C(CO)_15_]

IR (MeCN, 293 K) ν_CO_: 1873(s), 1688(w) cm^–1^.

#### [NMe_3_CH_2_Ph]_3_[Fe_5_C(CO)_13_(COMe)]

IR (MeCN, 293 K) ν_CO_: 1988(w), 1921(s), 1793(w) cm^–1^.

### 4.7 Reaction of [NEt_4_]_4_[Fe_6_C(CO)_15_] with CF_3_SO_3_Me and Na_2_CO_3_: Synthesis of [NEt_4_]_3_[H_3_O][Fe_6_C(CO)_14_(CO_3_)] and [NEt_4_]_3_[Fe_5_C(CO)_13_(COMe)]

A solution of CF_3_SO_3_Me (23 μL, 0.203 mmol) in MeCN (3 mL) was added dropwise to a solution of [NEt_4_]_4_[Fe_6_C(CO)_15_] (0.450 g, 0.350 mmol) in MeCN (20 mL) containing also Na_2_CO_3_ (0.100 g, 0.943 mmol). The resulting solution was stirred at room temperature for 7 h, and then, the solvent was removed under vacuum. The solid residue was washed with H_2_O (3 × 20 mL) and toluene (2 × 20 mL), and extracted with THF (20 mL), acetone (20 mL) and MeCN (20 mL). The residue was dried under vacuum after each extraction. The THF solution contained [NEt_4_]_2_[Fe_6_C(CO)_16_], as evidenced by IR analysis (ν_CO_ 1964(vs) cm^–1^). Slow diffusion of *n*-hexane (40 mL) on the acetone solution afforded crystals of [NEt_4_]_3_[H_3_O][Fe_6_C(CO)_14_(CO_3_)] suitable for SC-XRD analyses (yield 0.11 g, 26% based on Fe). Slow diffusion of *n*-hexane (2 mL) and di-isopropyl ether (30 mL) on the MeCN solution afforded crystals of [NEt_4_]_3_[Fe_5_C(CO)_13_(COMe)] (polymorph with space group *C*2) suitable for SC-XRD analyses (yield 0.09 g, 20% based on Fe).

#### [NEt_4_]_3_[H_3_O][Fe_6_C(CO)_14_(CO_3_)]

C_40_H_63_Fe_6_N_3_O_18_ (1209.03): calcd. C 39.74, H 5.25, N 3.48; found: C 39.42, H 5.01, N 3.66. IR (acetone, 293 K) ν_CO_: 2015(w), 1947(vs), 1785(w) cm^–1^. IR (solid, ATR, 293 K) ν_CO_: 1925(sh), 1900(vs), 1738(m) cm^–1^; ν_CO_3__: 1575–1615(sh) cm^–1^. ^1^H NMR (CD_3_CN, 298 K) δ_H_: 3.20 (br, CH_2_, cation), 1.23 (br, CH_3_, cation) ppm. ^13^C{^1^H} NMR (CD_3_CN, 298 K) δ_C_: 478.4 (Fe_6_*C*), 229.0, 227.2, 223.4, 221.1 (CO), 53.2 (CH_2_, cation), 7.8 (CH_3_, cation).

#### [NEt_4_]_3_[Fe_5_C(CO)_13_(COMe)]

C_40_H_63_Fe_5_N_3_O_14_ (1089.18): calcd. C 44.11, H 5.83, N 3.86; found: C 39.95, H 5.97, N 4.04. IR (MeCN, 293 K) ν_CO_: 1988(w), 1921(s), 1793(w) cm^–1^.

### 4.8 Electrochemical and Spectroelectrochemical Studies

Electrochemical measurements were performed with a PalmSens4 instrument interfaced to a computer employing PSTrace5 electrochemical software. CV measurements were carried out at room temperature under Ar in MeCN solutions containing [N*^n^*Bu_4_][PF_6_] (0.1 mol of dm^–3^) as the supporting electrolyte. Anhydrous MeCN (Sigma-Aldrich) was stored under argon over 3 Å molecular sieves. Electrochemical grade [N*^n^*Bu_4_][PF_6_] was purchased from Fluka and used without further purification. Cyclic voltammetry was performed in a three-electrode cell, the working and counter electrodes consisted, respectively, of a Pt disk sealed in a PEEK tube and a Pt plate, sealed in a glass tube. A leakless miniature Ag/AgCl/KCl electrode (eDAQ) was employed as a reference. The three-electrode home-built cell was predried by heating under vacuum and filled with argon. The Schlenk-type construction of the cell maintained anhydrous and anaerobic conditions. The solution of supporting electrolyte, prepared under argon, was introduced into the cell and the CV of the solvent was recorded. The analyte was then introduced, and voltammograms were recorded. Under the present experimental conditions, the one-electron oxidation of ferrocene occurs at *E*° = +0.42 V vs Ag/AgCl.

Infrared (IR) spectroelectrochemical measurements were carried out using an optically transparent thin-layer electrochemical (OTTLE) cell^61^ equipped with CaF_2_ windows, platinum mini-grid working and auxiliary electrodes and silver wire pseudo reference electrode, whose potential is different from that of the Ag/AgCl/KCl electrode used in CV and can change from one experiment to another. In the IR SEC experiments, as specified in the main text, we reported the potentials measured vs this pseudo reference. During the microelectrolysis procedures, the electrode potential was controlled by a PalmSens4 instrument interfaced to a computer employing PSTrace5 electrochemical software. Argon-saturated MeCN solutions of the compound under study, containing [N*^n^*Bu_4_][PF_6_] (0.1 M) as the supporting electrolyte, were used. The *in situ* spectroelectrochemical experiments were performed by collecting spectra of the solution at constant time intervals during the oxidation or reduction obtained by continuously increasing or lowering the initial working potential at a scan rate of 1.0 mV/s. IR spectra were recorded on a PerkinElmer Spectrum 100 FT-IR spectrophotometer.

### 4.9 X-ray Crystallographic Study

Crystal data and collection details for [NEt_4_]_3_[H_3_O][Fe_6_C(CO)_14_(CO_3_)] (two independent crystals have been collected), [NEt_4_]_2_[Fe_6_C(CO)_15_(PTA)], [NMe_3_CH_2_Ph]_4_[Fe_6_C(CO)_15_] (two different polymorphs, both with space group *C*2/*c*), [NMe_3_CH_2_Ph]_2_[Fe_6_C(CO)_16_], [NEt_4_]_2_[Fe_5_C(CO)_13_(PPh_3_)], [NEt_4_]_3_[Fe_5_C(CO)_13_(COMe)] (two polymorphs, space group *P*1 and *C*2), [NMe_3_CH_2_Ph]_3_[Fe_5_C(CO)_13_(COMe)], [NMe_3_CH_2_Ph]_2_[Fe_5_C(CO)_14_] (two different polymorphs, both with space group *P*1̅) are reported in Table S5 in the Supporting Information. The diffraction experiments were carried out on a Bruker APEX II diffractometer equipped with a PHOTON2 detector using Mo–Kα radiation. Data were corrected for Lorentz polarization and absorption effects (empirical absorption correction SADABS).^64^ Structures were solved by direct methods and refined by full-matrix least-squares based on all data using *F*.^2^^65^ Hydrogen atoms were fixed at calculated positions and refined by a riding model unless otherwise stated. All nonhydrogen atoms were refined with anisotropic displacement parameters. Further details may be found in the Supporting Information.

### 4.10 Computational Details

Geometry optimizations were carried out with the r^2^-SCAN-3c method,^66^ based on the *meta*-GGA r^2^SCAN functional and a triple-ζ Gaussian atomic orbital basis set, with corrections for London dispersion and basis set superposition error.^67−70^ The C-PCM implicit solvation model (acetonitrile) was added to all of the calculations.^71^ IR simulations were carried out using the harmonic approximation. Thermal corrections were calculated at 298.15 K. Calculations were carried out using ORCA 5.0.3 and the output files were analyzed with Multiwfn, version 3.8.^72−74^ The Cartesian coordinates of the DFT-optimized structures are provided as Supporting Information (.xyz file format).

## Supporting Information Available

IR spectra; NMR spectra; supplementary electrochemical and spectroelectrochemical figures; X-ray crystallographic study; computational studies (PDF)
DFT-optimized coordinates (XYZ)

## Supplementary Material

ic5c01014_si_001.pdf

ic5c01014_si_002.xyz
