# Supplementary material for: Chemical and Electrochemical Investigation of the Oxidation of a Highly Reduced Fe6C Iron Carbide Carbonyl Cluster: A Synthetic Route to Heteroleptic Fe6C and Fe5C Clusters
Source: Inorg Chem. 2025 May 6;64(19):9744–57. doi: 10.1021/acs.inorgchem.5c01014 (PMC12093381; doi:10.1021/acs.inorgchem.5c01014)
Supplement: Supplementary file 1 — ic5c01014_si_001.pdf [file ic5c01014_si_001.pdf]

Supporting Information for

Chemical and Electrochemical Investigation of the Oxidation of a Highly Reduced Fe_6C Iron Carbide Carbonyl Cluster: a Synthetic Route to Heteroleptic Fe_6C and Fe_5C Clusters

Tiziana Funaioli,[a] Cristiana Cesari,[b] Beatrice Berti,[b] Marco Bortoluzzi,[c] Cristina Femoni,[b] Francesca Forti,[b] Maria Carmela Iapalucci,[b] Giorgia Scorzoni[b] and Stefano Zacchini[b]*

[a] Dipartimento di Chimica e Chimica Industriale, Università di Pisa, Via G. Moruzzi 13 - 56124, Pisa, Italy.

[b] Dipartimento di Chimica Industriale "Toso Montanari", Università di Bologna, Via P. Gobetti 85 - 40129 Bologna. Italy. E-mail: stefano.zacchini@unibo.it

[c] Dipartimento di Scienze Molecolari e Nanosistemi, Ca' Foscari University of Venice, Via Torino 155 – 30175 Mestre (Ve), Italy.

	Page/s
IR spectra	S2-S6
NMR spectra	S7-S12
Supplementary electrochemical and spectroelectrochemical figures	S13-S16
X-Ray crystallographic study	S17-S30
Computational studies	S31-S53
References	S54

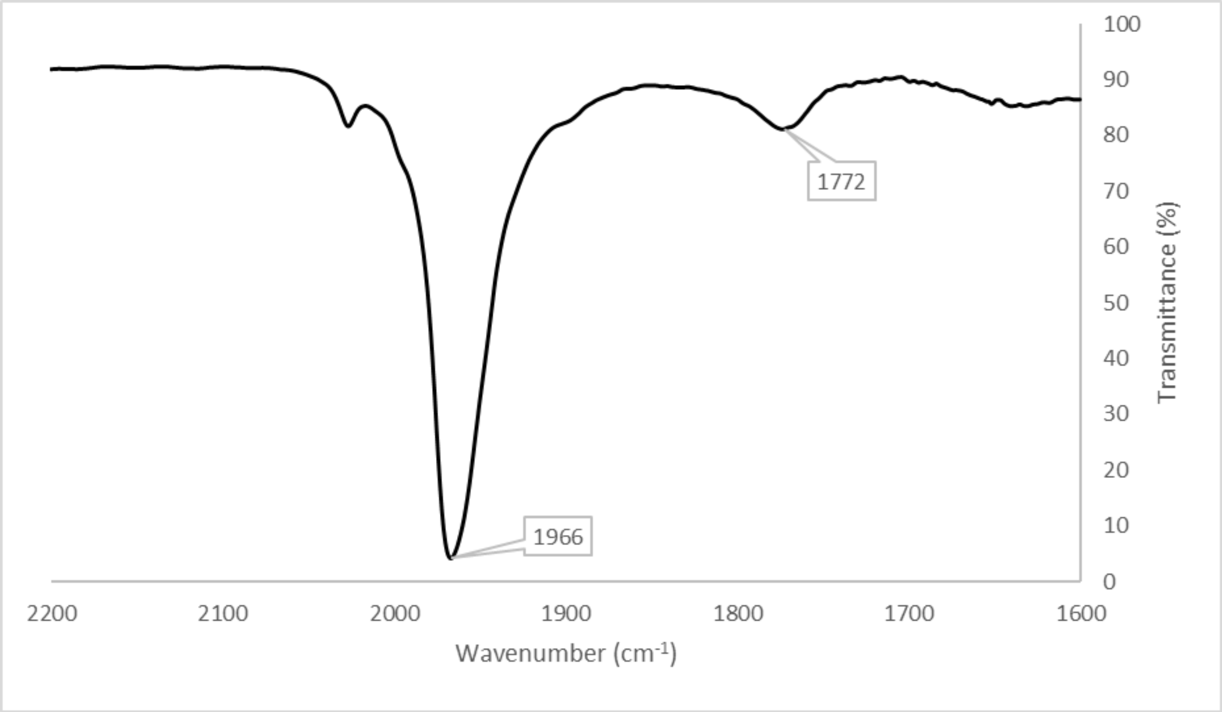

Figure S1. IR spectrum in the ν_{CO} region of $[NEt_4]_2[Fe_6C(CO)_{16}]$ recorded in CH_3CN.

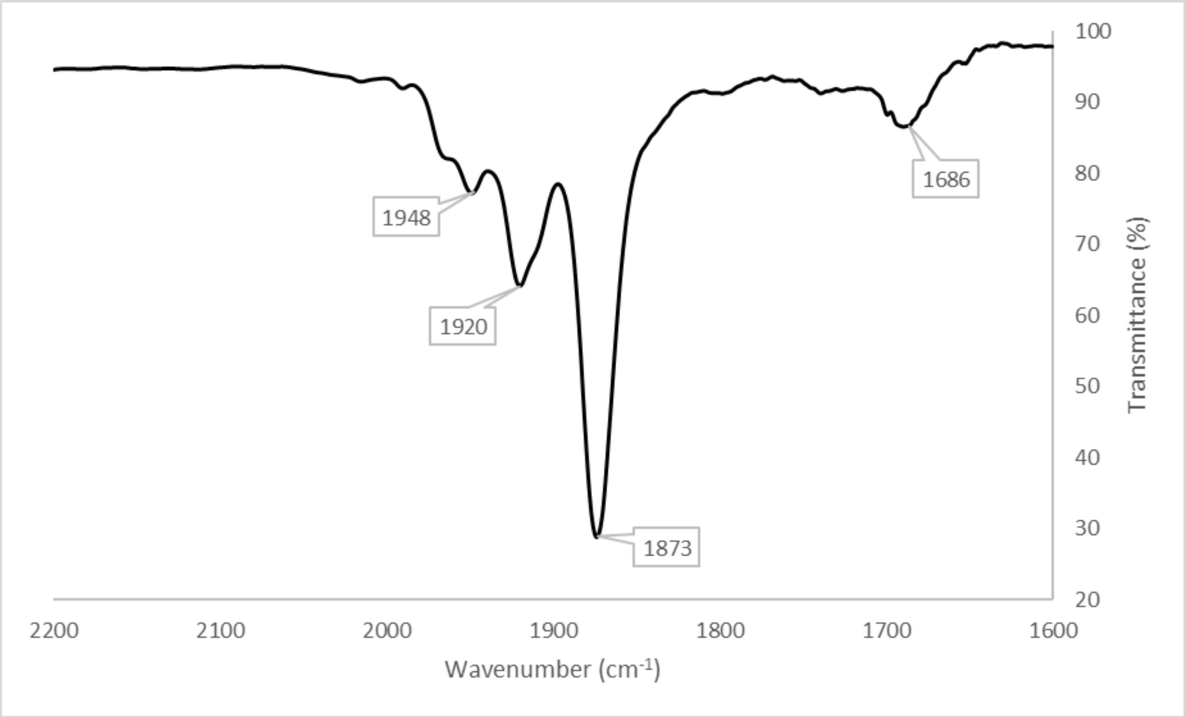

Figure S2. IR spectrum in the ν_{CO} region of $[NEt_4]_4[Fe_6C(CO)_{15}]$ recorded in CH_3CN.

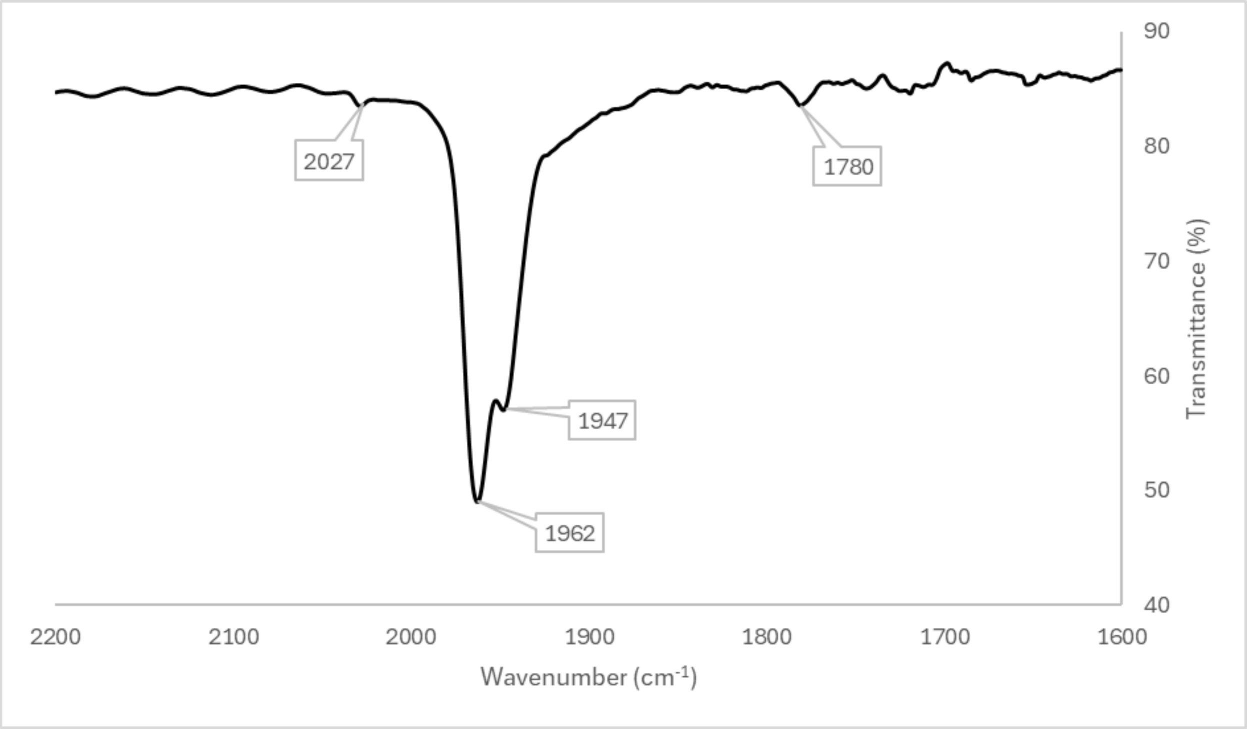

Figure S3. IR spectrum in the ν_{CO} region of $[NEt_4]_2[Fe_5C(CO)_{14}]$ recorded in THF.

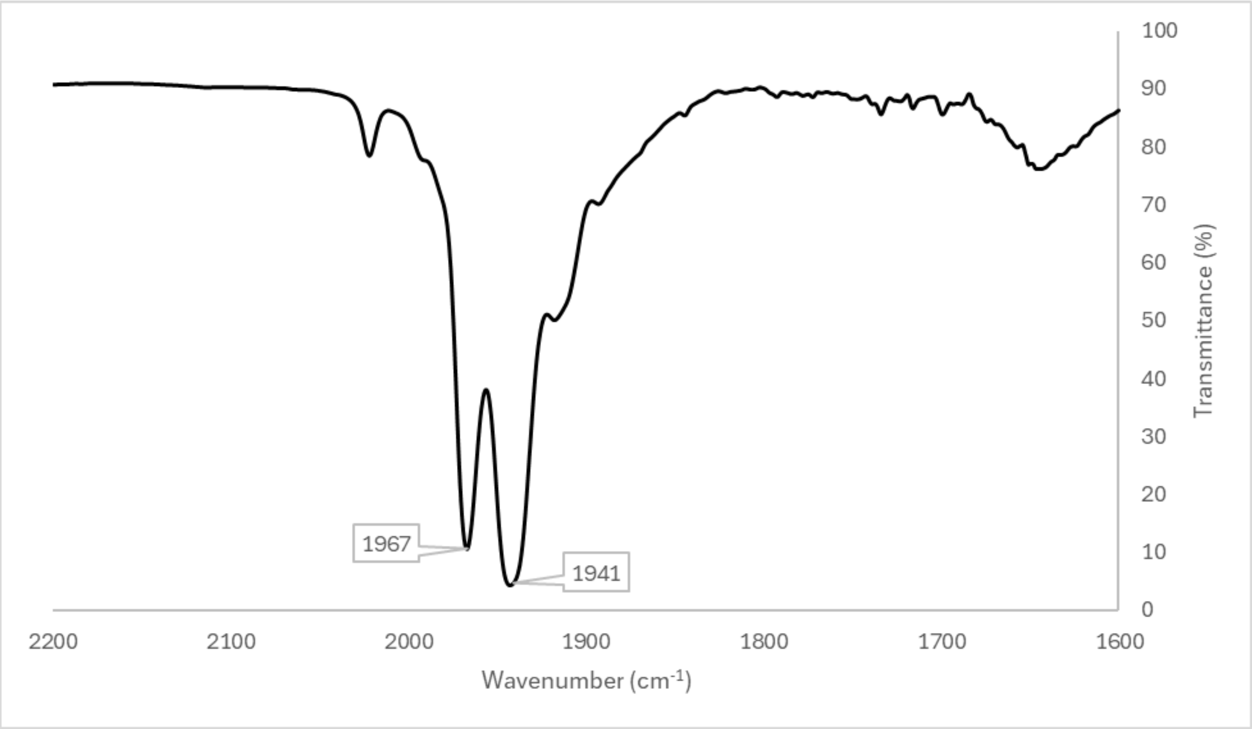

Figure S4. IR spectrum in the ν_{CO} region of $[NEt_4]_2[Fe_4C(CO)_{12}]$ recorded in THF.

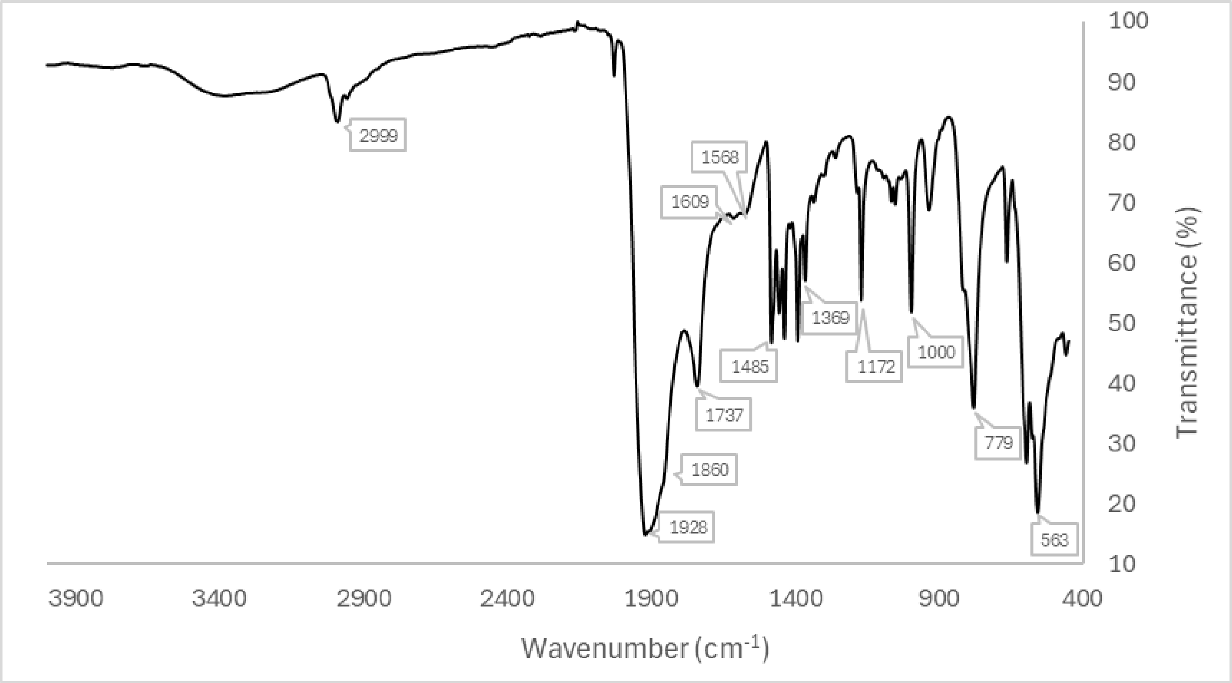

Figure S5. ATR-IR spectrum of $[NEt_4]_3[H_3O][Fe_6C(CO)_{14}(CO_3)]$ as a solid.

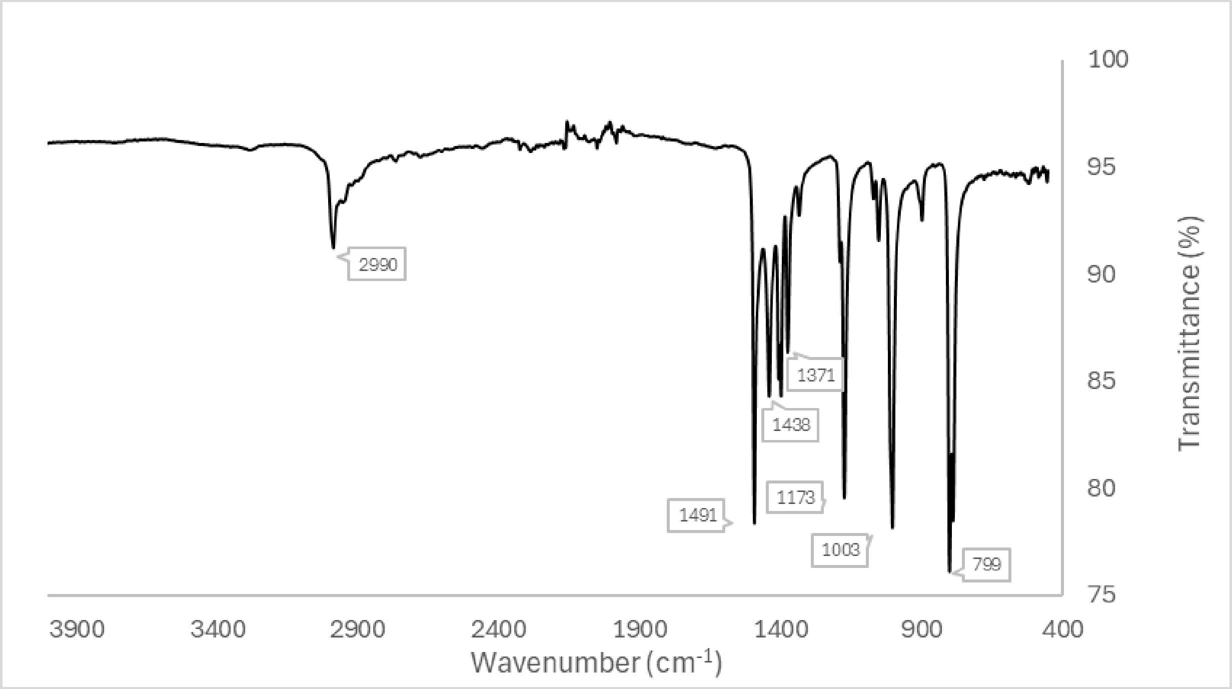

Figure S6. ATR-IR spectrum of $[NEt_4]Br$ as a solid.

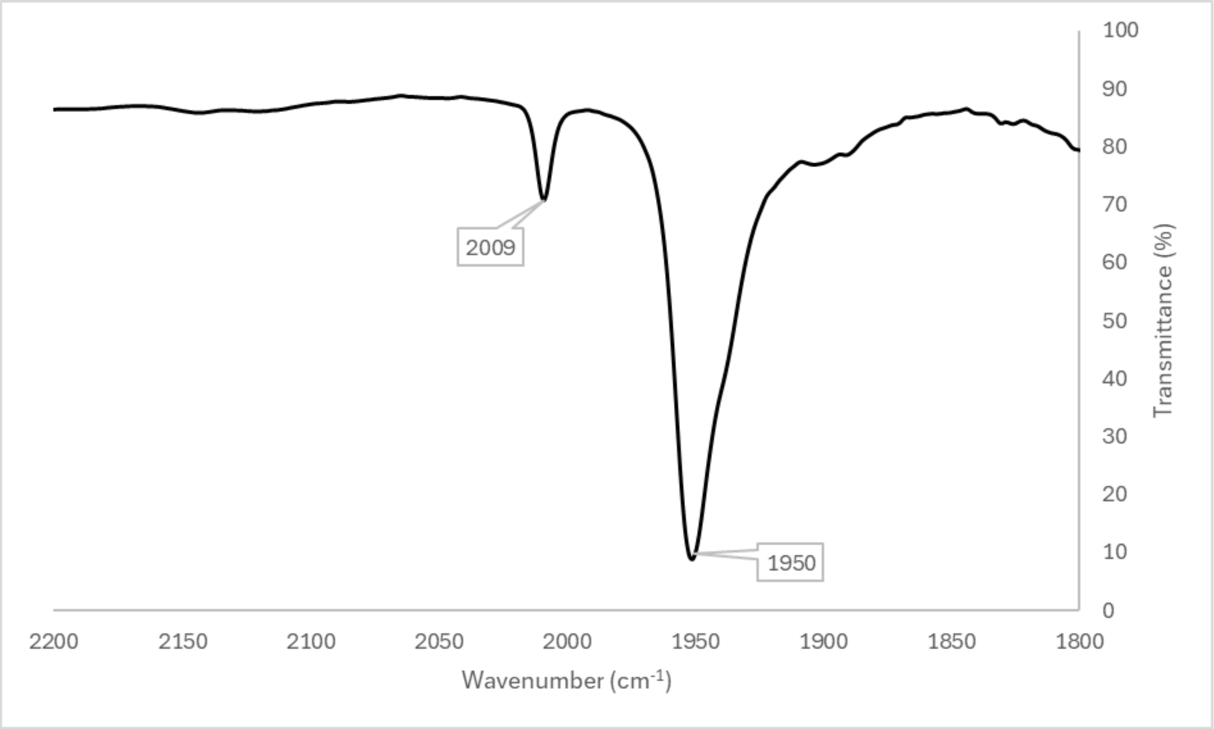

Figure S7. IR spectrum in the ν_{CO} region of $[NEt_4]_2[Fe_6C(CO)_{15}(PTA)]$ recorded in acetone.

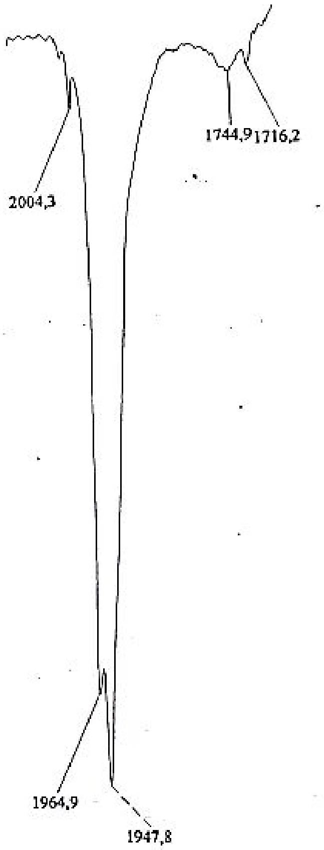

Figure S8. IR spectrum in the ν_{CO} region of $[NEt_4]_2[Fe_5C(CO)_{13}(PPh_3)]$ recorded in CH_3CN.

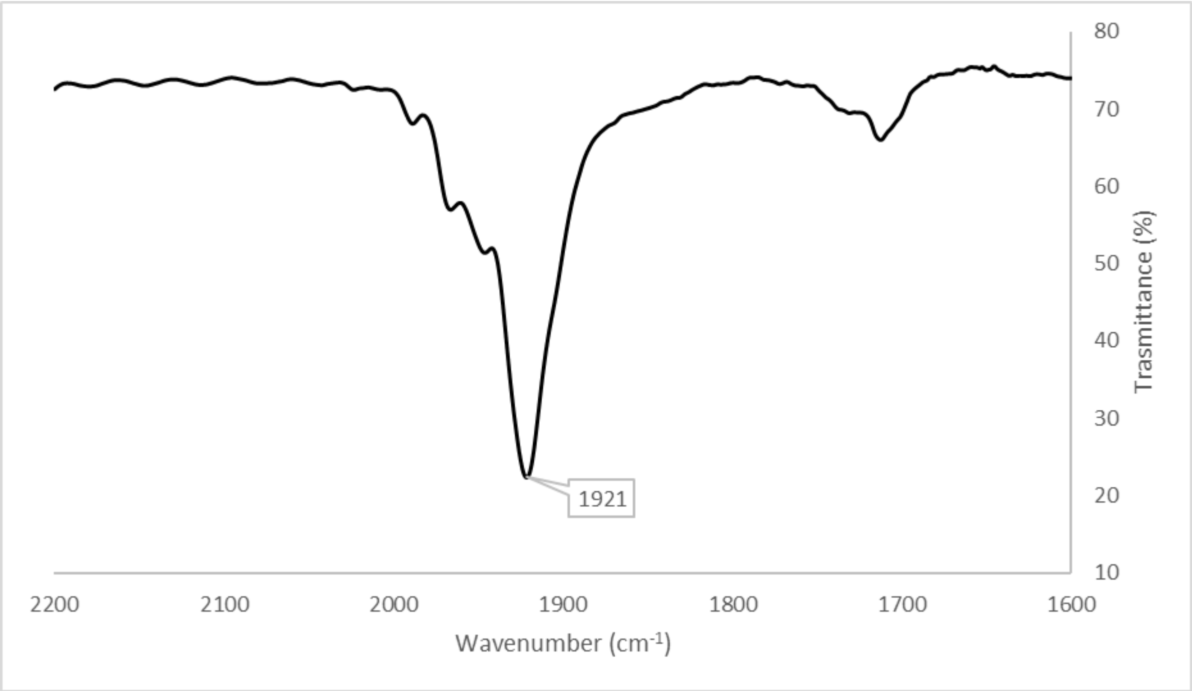

Figure S9. IR spectrum in the ν_{CO} region of $[NEt_4]_3[Fe_5C(CO)_{13}(COMe)]$ recorded in CH_3CN.

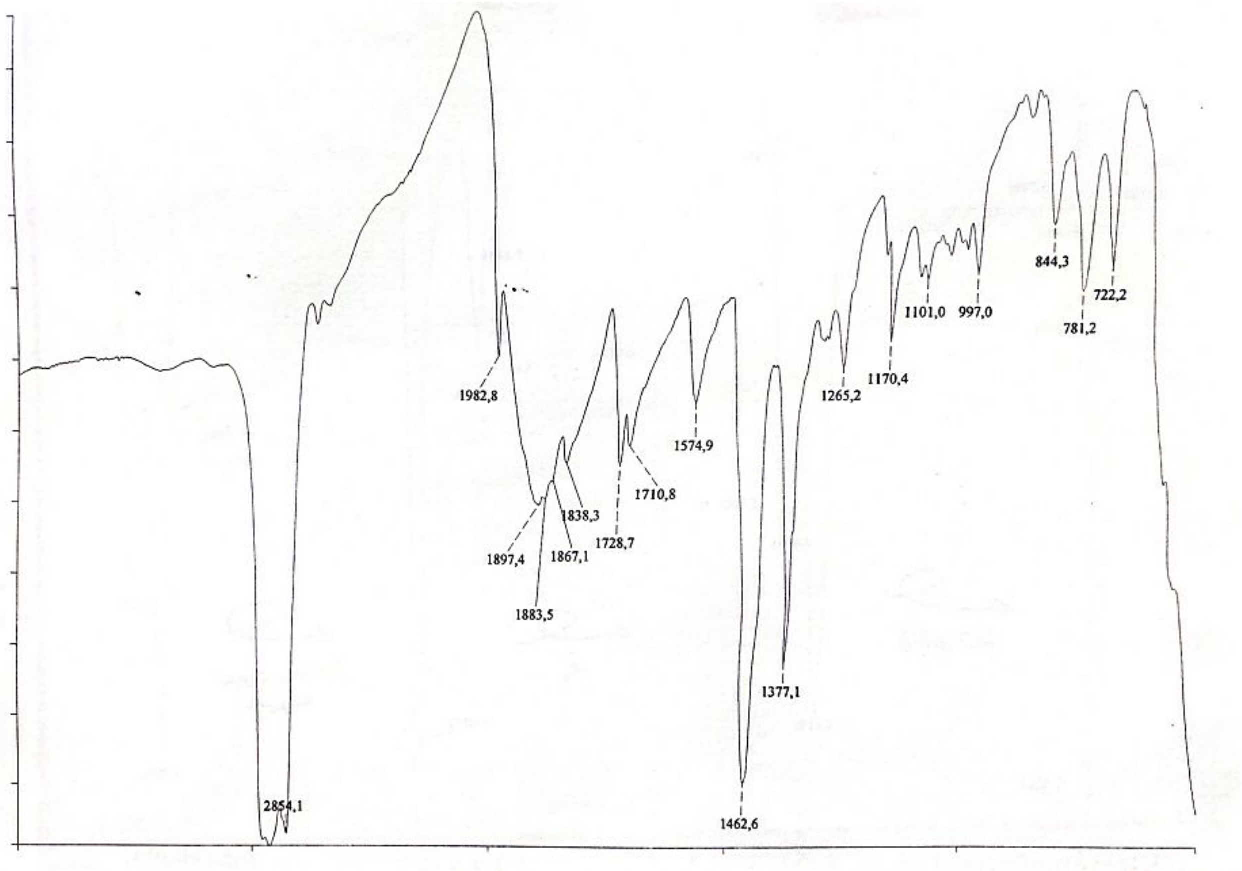

Figure S10. IR spectrum of $[NEt_4]_3[Fe_5C(CO)_{13}(COMe)]$ in nujol mull.

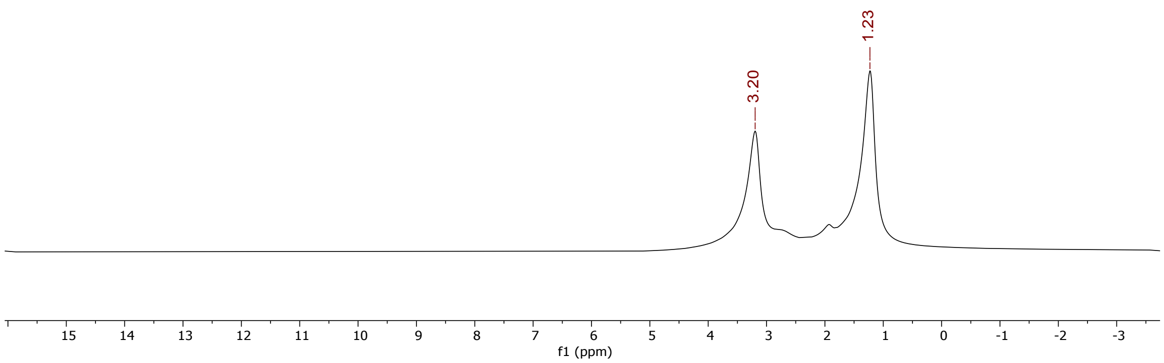

Figure S11. ^{1}H NMR spectrum of $[NEt_4]_3[H_3O][Fe_6C(CO)_{14}(CO_3)]$ recorded in CD_3CN at 298 K.

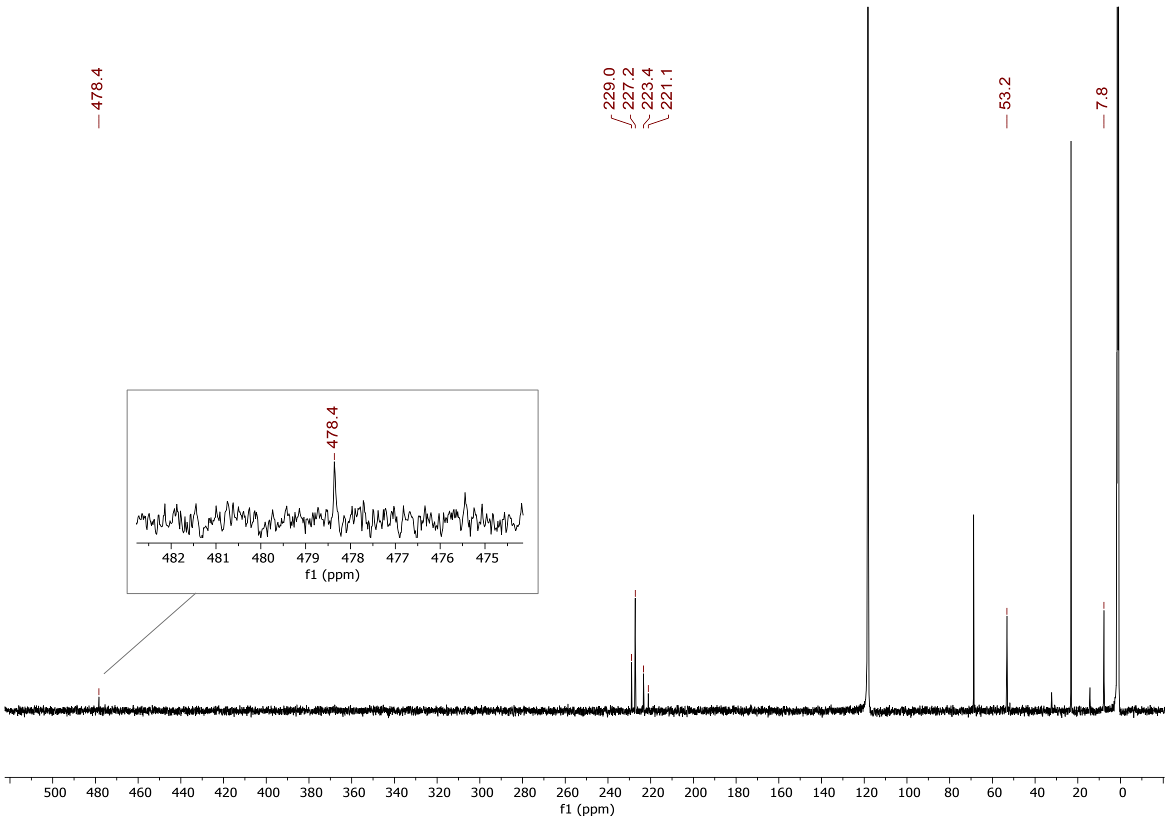

Figure S12. $^{13}C\{^{1}H\}$ NMR spectrum of $[NEt_4]_3[H_3O][Fe_6C(CO)_{14}(CO_3)]$ recorded in CD_3CN at 298 K.

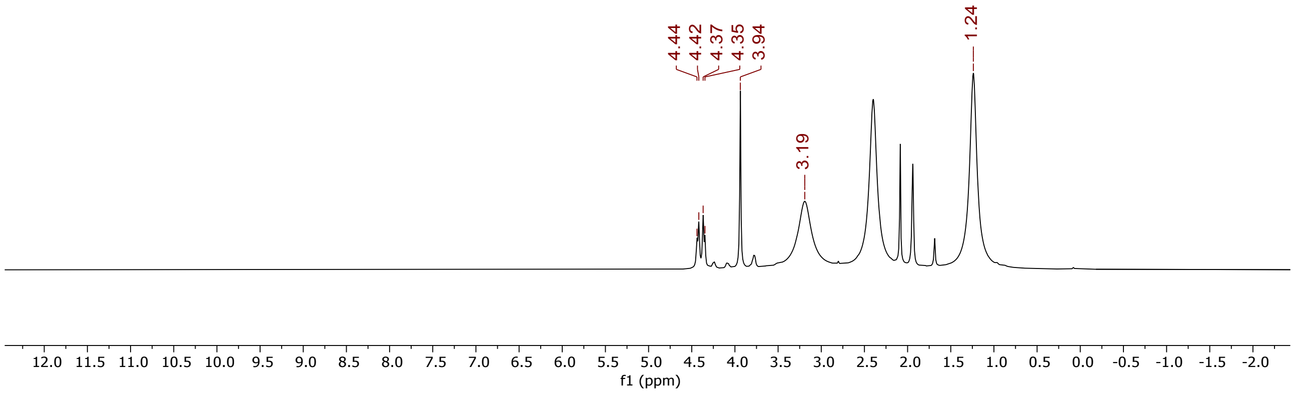

Figure S13. ^{1}H NMR spectrum of $[NEt_4]_2[Fe_6C(CO)_{15}(PTA)]$ recorded in CD_3CN at 298 K.

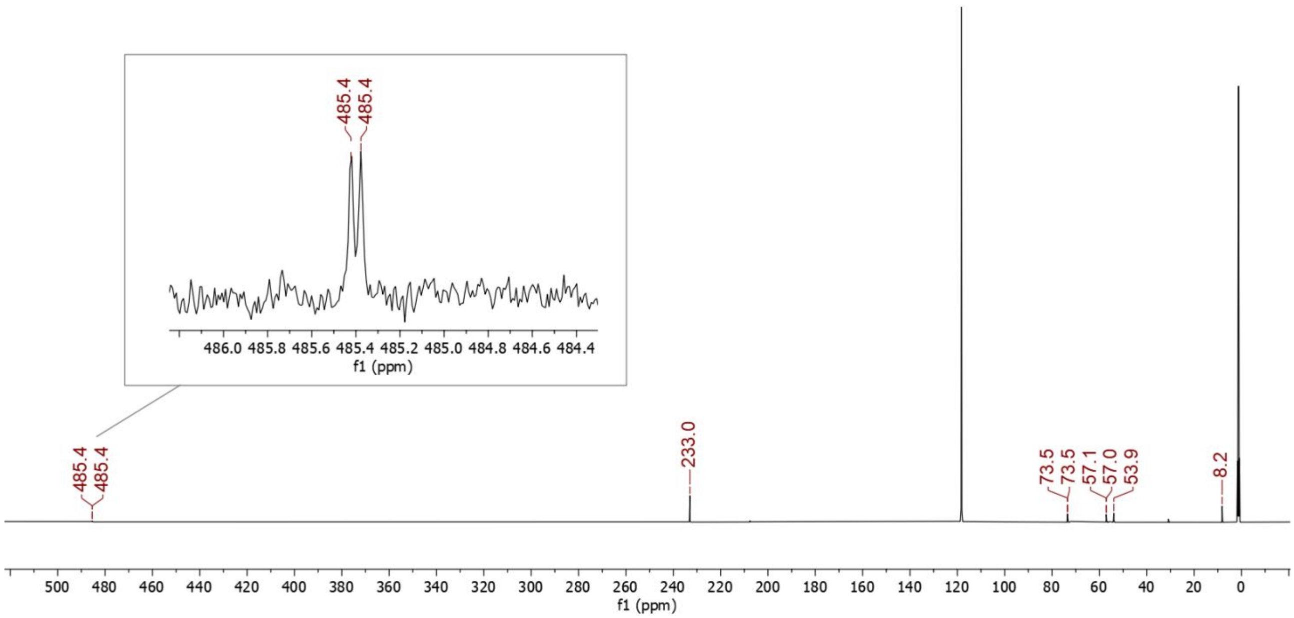

Figure S14. $^{13}C\{^{1}H\}$ NMR spectrum of $[NEt_4]_2[Fe_6C(CO)_{15}(PTA)]$ recorded in CD_3CN at 298 K.

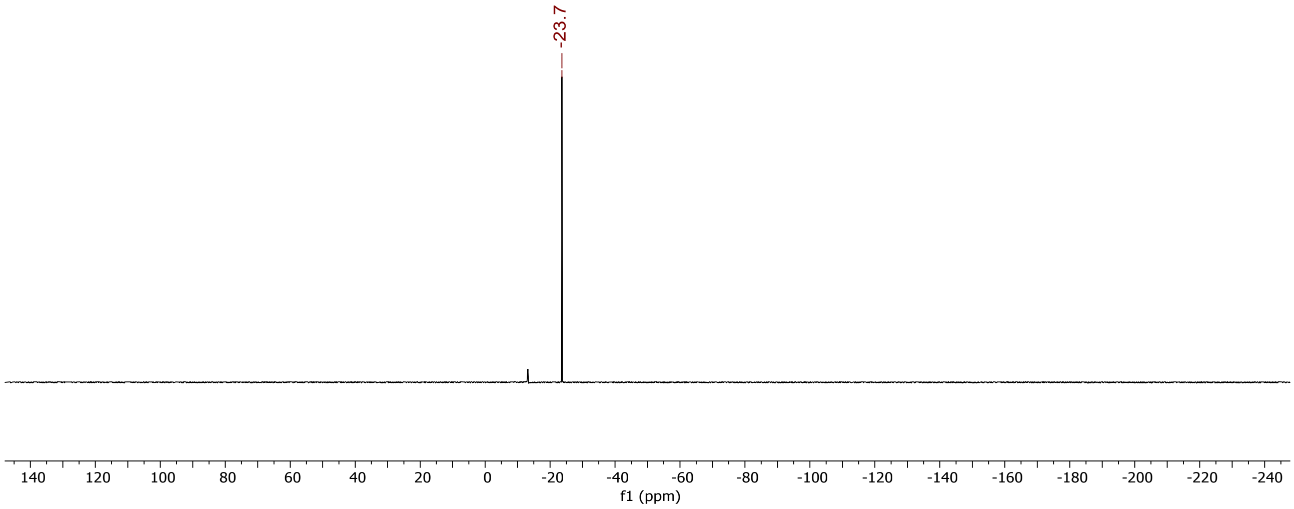

Figure S15. $^{31}P\{^{1}H\}$ NMR spectrum of $[NEt_4]_2[Fe_6C(CO)_{15}(PTA)]$ recorded in CD_3CN at 298 K.

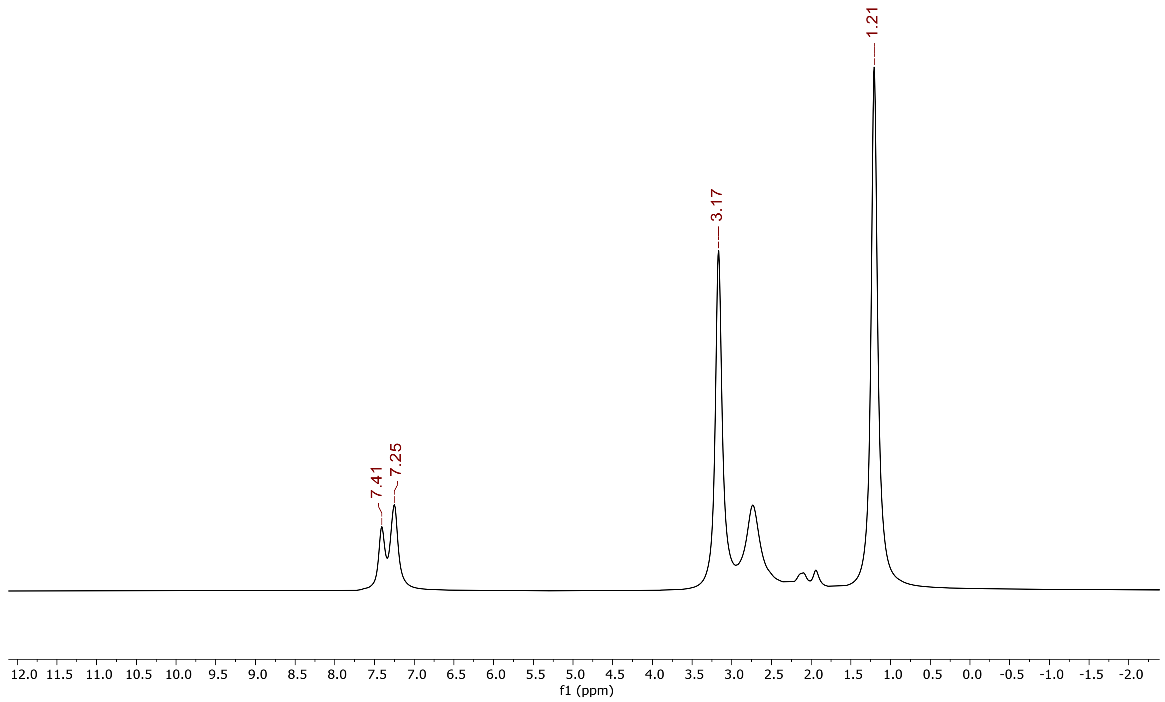

Figure S16. ^{1}H NMR spectrum of $[NEt_4]_2[Fe_5C(CO)_{13}(PPh_3)]$ recorded in CD_3CN at 298 K

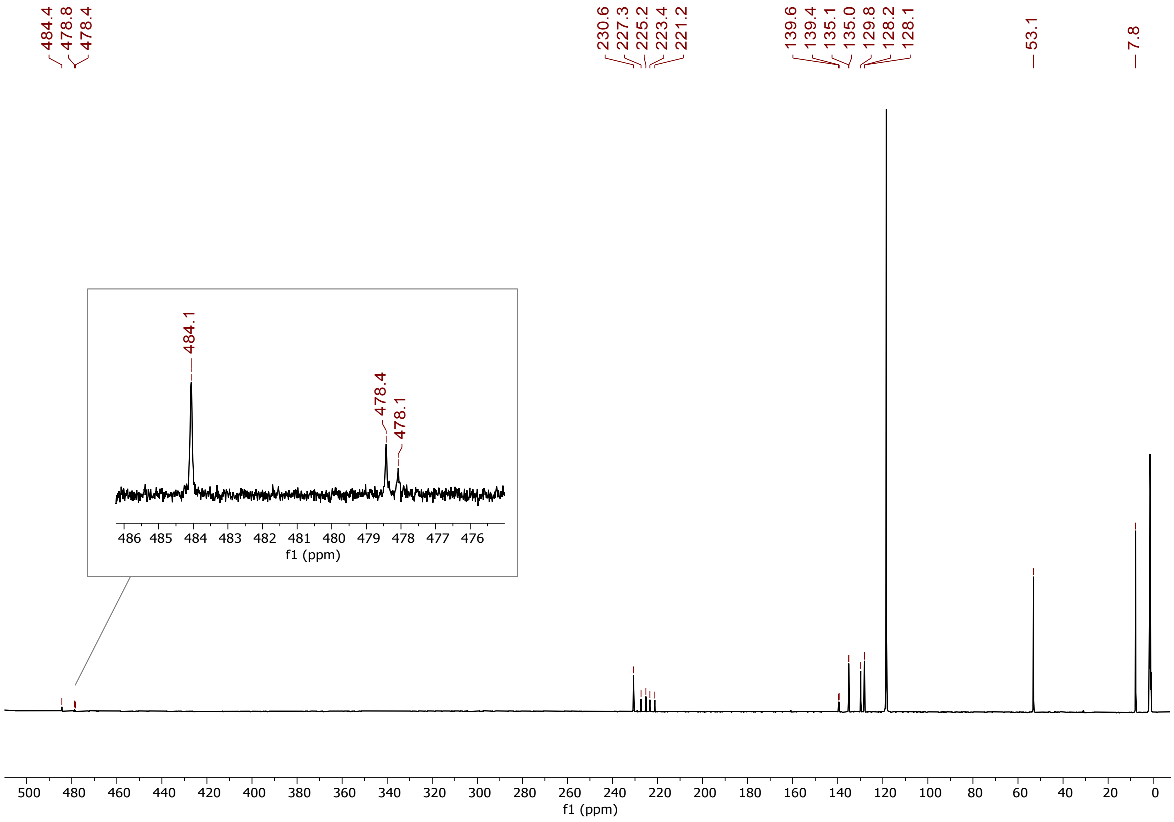

Figure S17. $^{13}C\{^{1}H\}$ NMR spectrum of $[NEt_4]_2[Fe_5C(CO)_{13}(PPh_3)]$ recorded in CD_3CN at 298 K. Its carbide resonates at δ_C = 478.4 ppm. The resonances at 484.1 and 478.1 ppm are attributable to $[NEt_4]_2[Fe_6C(CO)_{16})]$ and $[NEt_4]_2[Fe_5C(CO)_{14}]$, respectively.

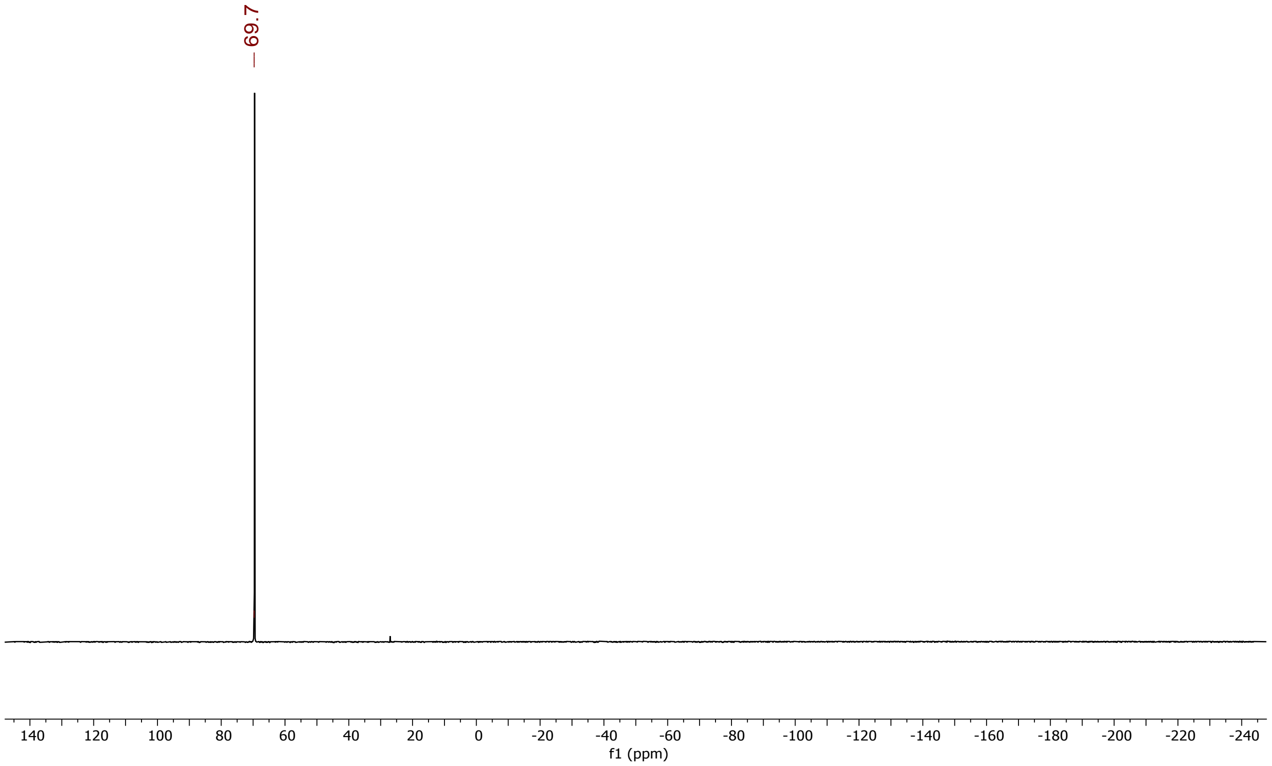

Figure S18. $^{31}P\{^{1}H\}$ NMR spectrum of $[NEt_4]_2[Fe_5C(CO)_{13}(PPh_3)]$ recorded in CD_3CN at 298 K.

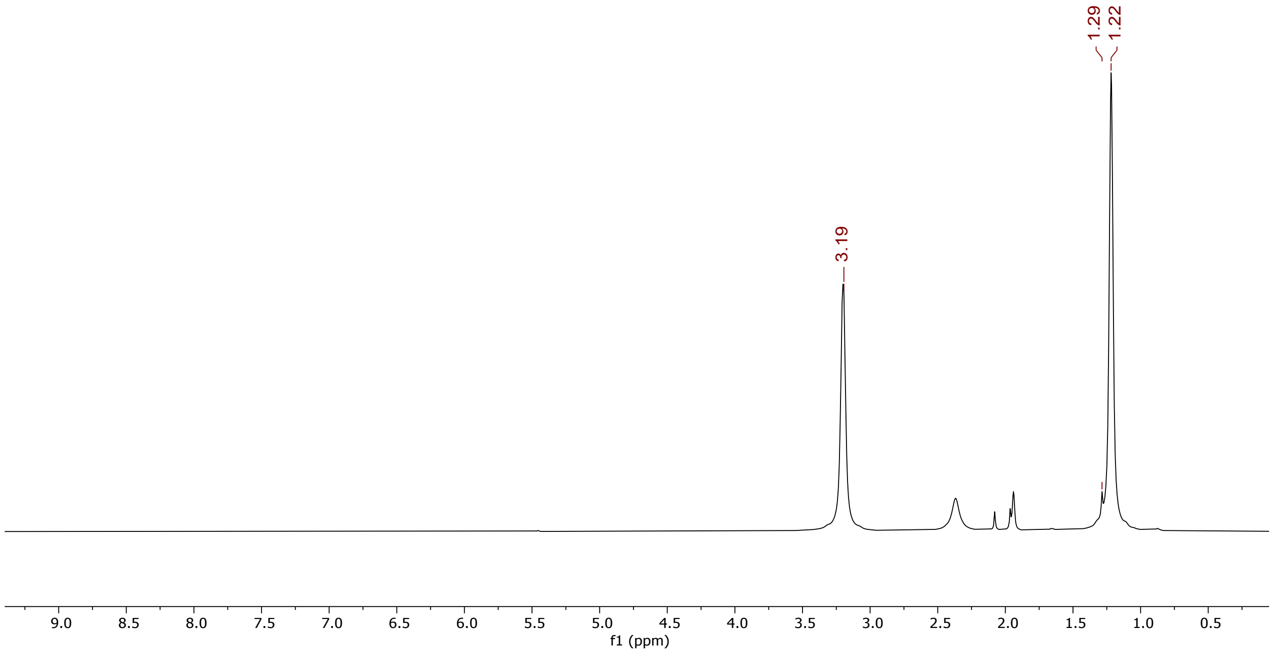

Figure S19. ^{1}H NMR spectrum of $[NEt_4]_3[Fe_5C(CO)_{13}(COMe)]$ recorded in CD_3CN at 298 K.

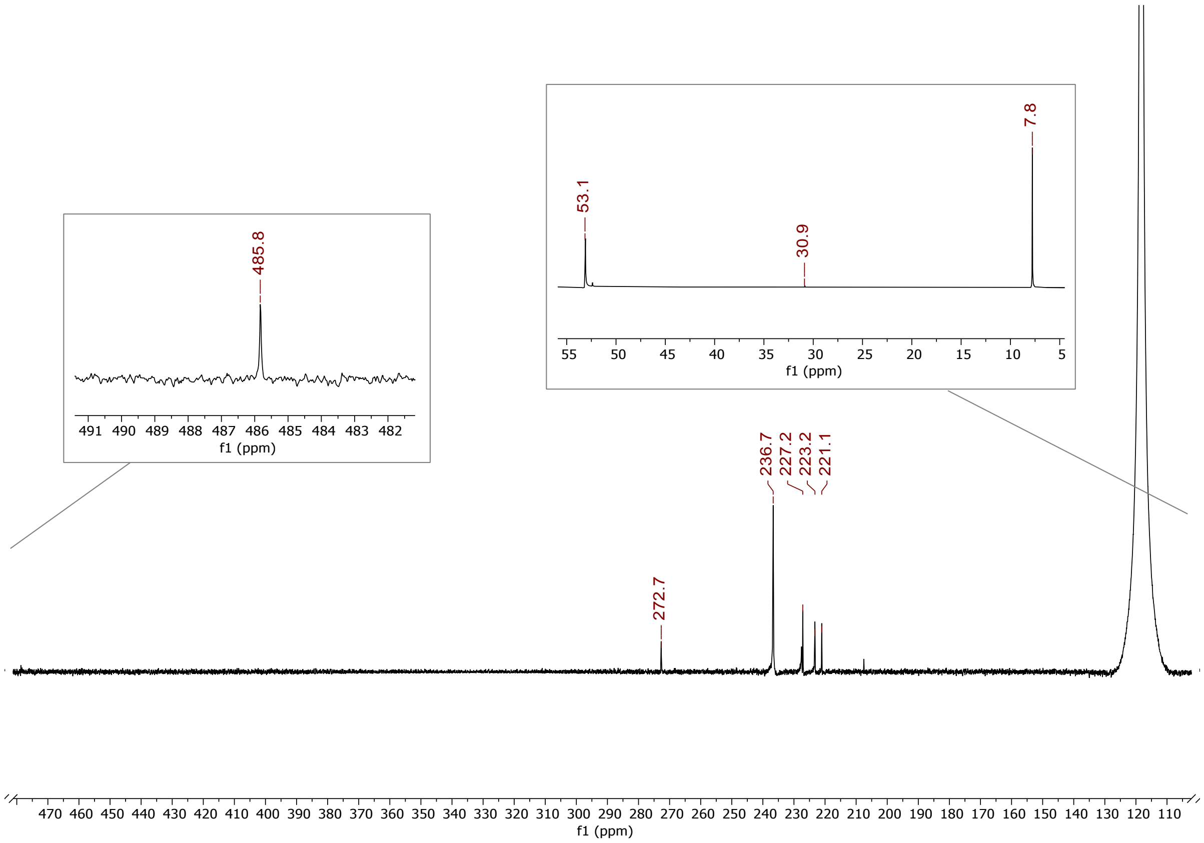

Figure S20. $^{13}C\{^{1}H\}$ NMR spectrum of $[NEt_4]_3[Fe_5C(CO)_{13}(COMe)]$ recorded in CD_3CN at 298 K.

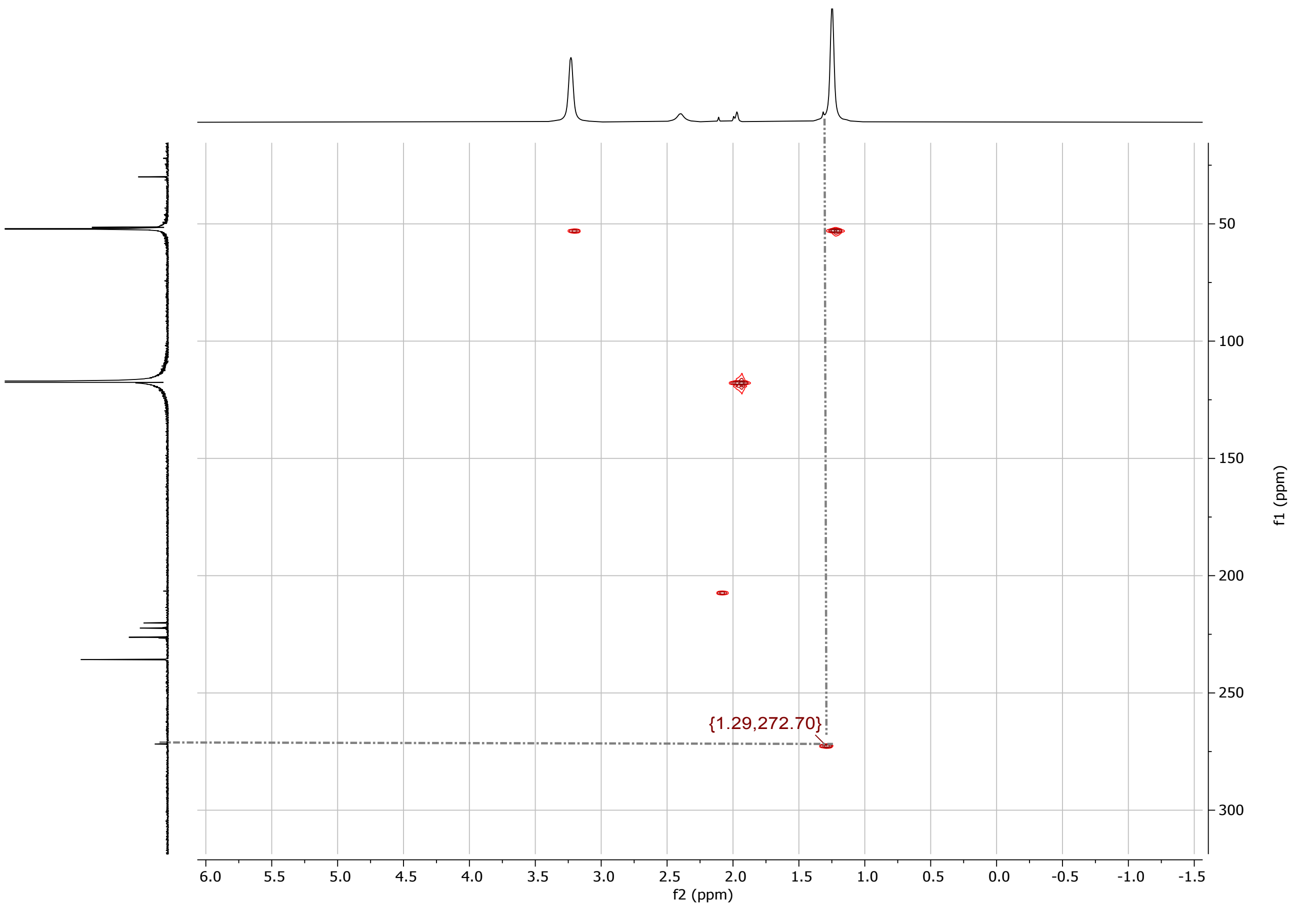

Figure S21. HMBC spectrum of $[NEt_4]_3[Fe_5C(CO)_{13}(COMe)]$ recorded in CD_3CN at 298 K.

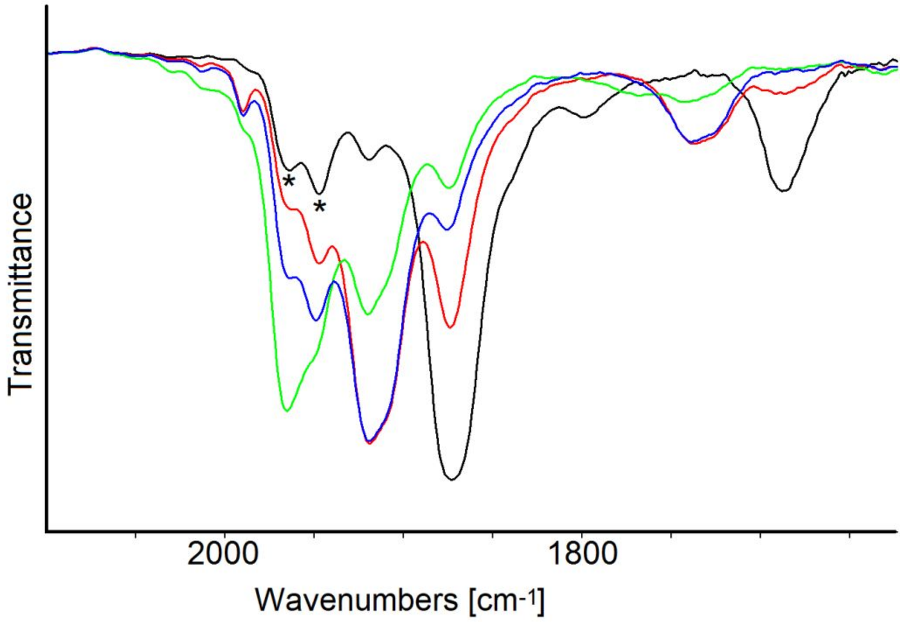

Figure S22. Selected IR spectra from Figure 6 at the WE potential of: –0.2 V (black line, starred bands are due to impurities); +0.44 V (red line); +0.68 (blue line); +1.0 V (green line). The absorptions of the solvent and supporting electrolyte have been subtracted.

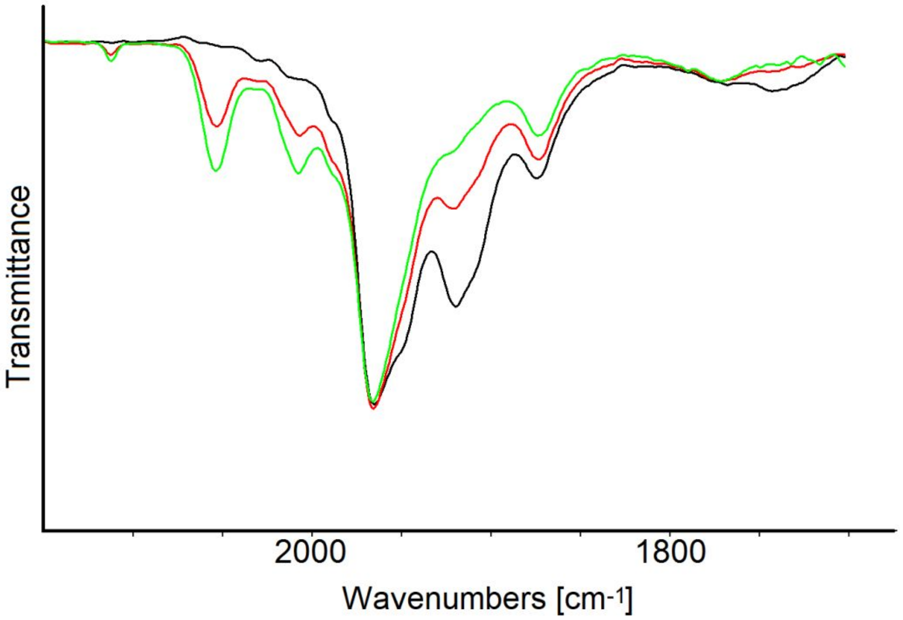

Figure S23. IR spectra of a CH_3CN solution of $[Fe_6C(CO)_{15}]^{4-}$ recorded in an OTTLE cell during the progressive increase of the potential from +1.0 to +1.1 V (*vs* Ag pseudo-reference electrode). $[N^nBu_4][PF_6]$ (0.1 mol dm^{-3}) as the supporting electrolyte. The absorptions of the solvent and supporting electrolyte have been subtracted.

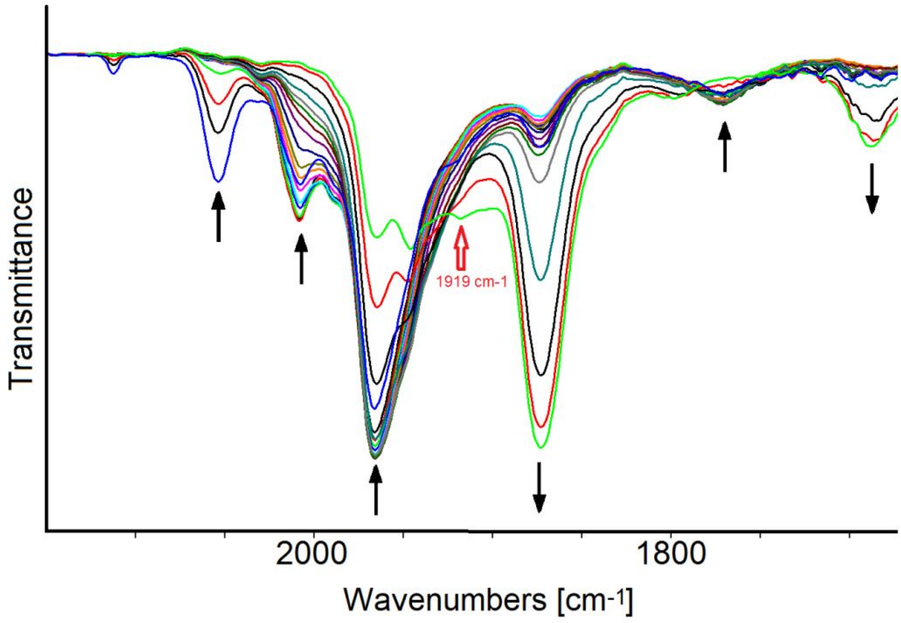

Figure S24. IR spectra of the CH_3CN solution of $[Fe_6C(CO)_{15}]^{4-}$, during the back reduction scan following the oxidation of Figure S23, as obtained by the progressive decrease of the WE potential from +1.1 up to –1.0 V (*vs* Ag pseudo-reference electrode, scan rate 2 mV s^{-1}). $[N^nBu_4][PF_6]$ (0.1 mol dm^{-3}) as the supporting electrolyte. The absorptions of the solvent and supporting electrolyte have been subtracted.

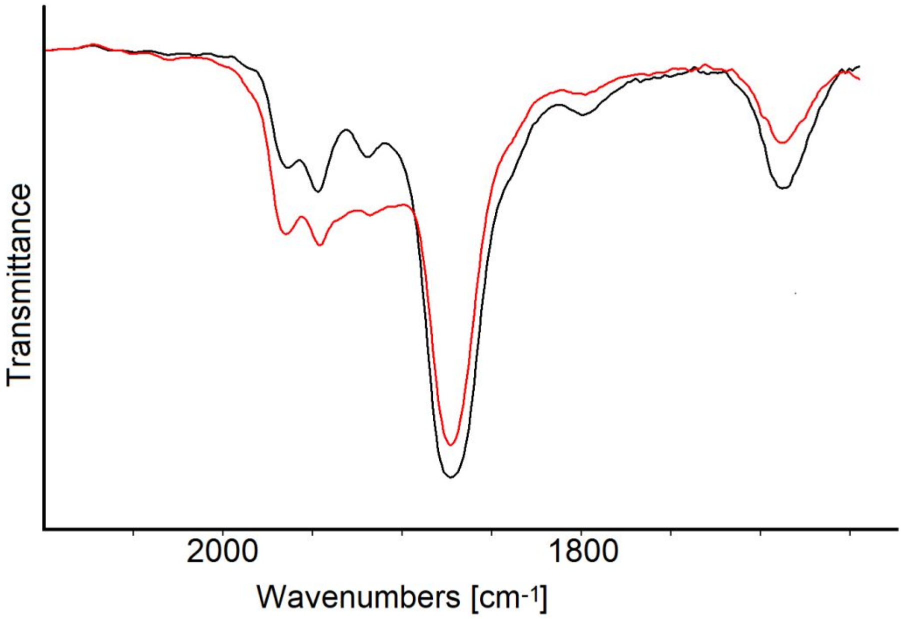

Figure S25. IR spectra of a CH_3CN solution of $[Fe_6C(CO)_{15}]^{4-}$ recorded in an OTTLE cell before (black line) and after (red line) the slow cyclic voltammetry between –1.0 and +1.1 V (*vs* Ag pseudo-reference electrode, scan rate 1 mV s^{-1}) $[N^nBu_4][PF_6]$ (0.1 mol dm^{-3}) as the supporting electrolyte. The absorptions of the solvent and supporting electrolyte have been subtracted.

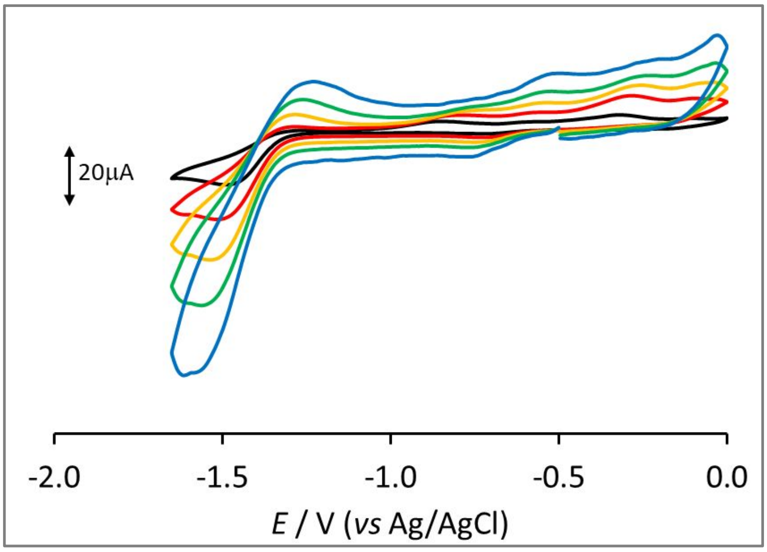

Figure S26. CV response of $[Fe_6C(CO)_{16}]^{2-}$ at a Pt electrode in CH_3CN solution between –1.65 and 0.0 V at increasing scan rates (0.1, 0.4, 1.0, 2.0 and 4.0 V s^{-1}). $[N^nBu_4][PF_6]$ (0.1 mol dm^{-3}) supporting electrolyte.

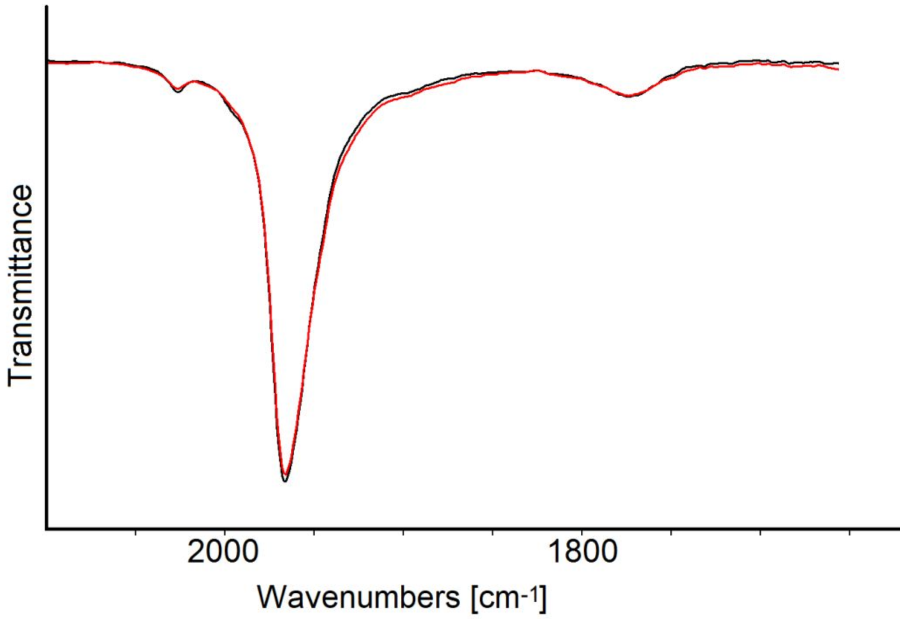

Figure S27. IR spectra of a CH_3CN solution of $[Fe_6C(CO)_{16}]^{2-}$ recorded in an OTTLE cell before (black line) and after (red line) a slow cyclic voltammetry between 0.0 and –1.2 V (*vs* Ag pseudo-reference electrode, scan rate 1 mV s^{-1}). $[N^nBu_4][PF_6]$ (0.1 mol dm^{-3}) as the supporting electrolyte. The absorptions of the solvent and supporting electrolyte have been subtracted.

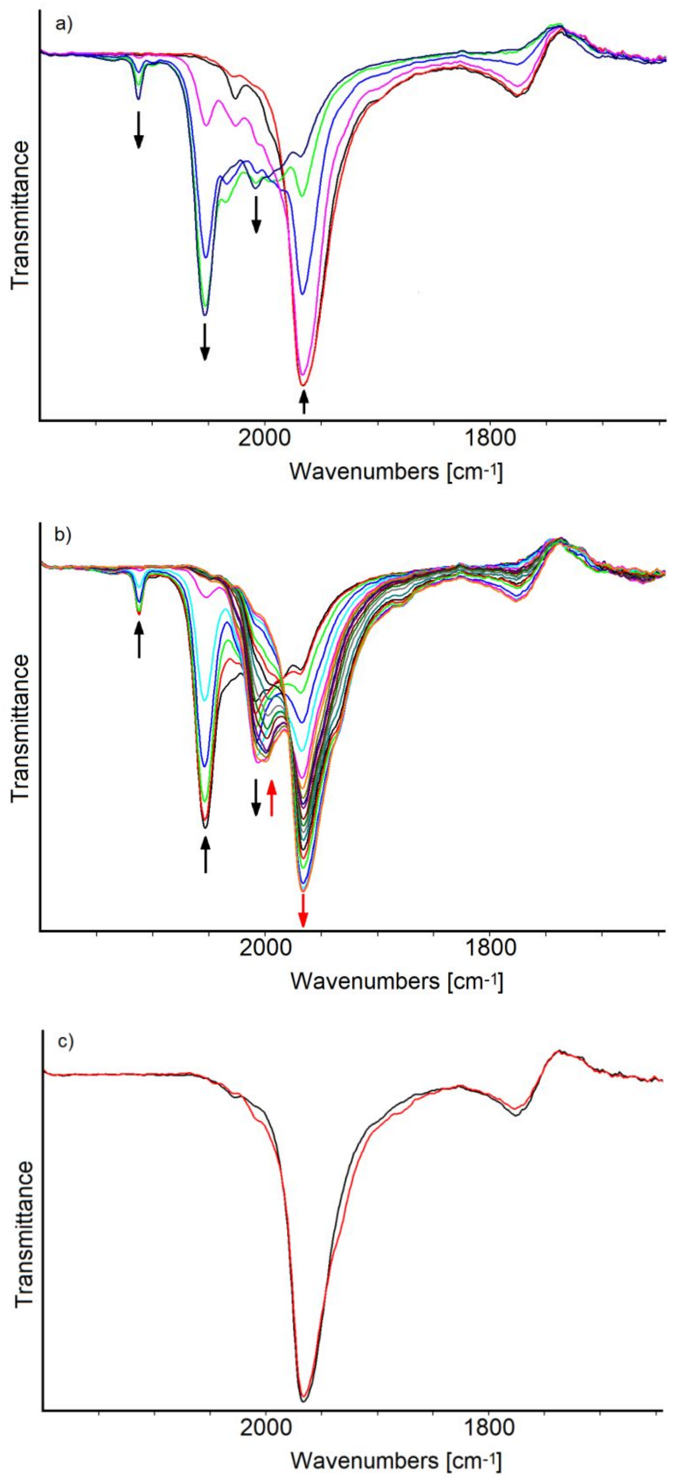

Figure 28. IR spectra of a CH_3CN solution of $[Fe_6C(CO)_{16}]^{2-}$ recorded in an OTTLE cell during a) the progressive increase of the potential from +0.2 to +0.6 V (*vs* Ag pseudo-reference electrode, scan rate 2 mV s^{-1}); b) the reduction back-scan from +0.6 to –0.6 V; c) before (black line) and after (red line) the slow cyclic voltammetry between –0.6 and +0.6 V (*vs* Ag pseudo-reference electrode, scan rate 2 mV s^{-1}) $[N^nBu_4][PF_6]$ (0.1 mol dm^{-3}) as the supporting electrolyte. The absorptions of the solvent and supporting electrolyte have been subtracted. The two colours (red and black) of the arrows indicate which bands change simultaneously.

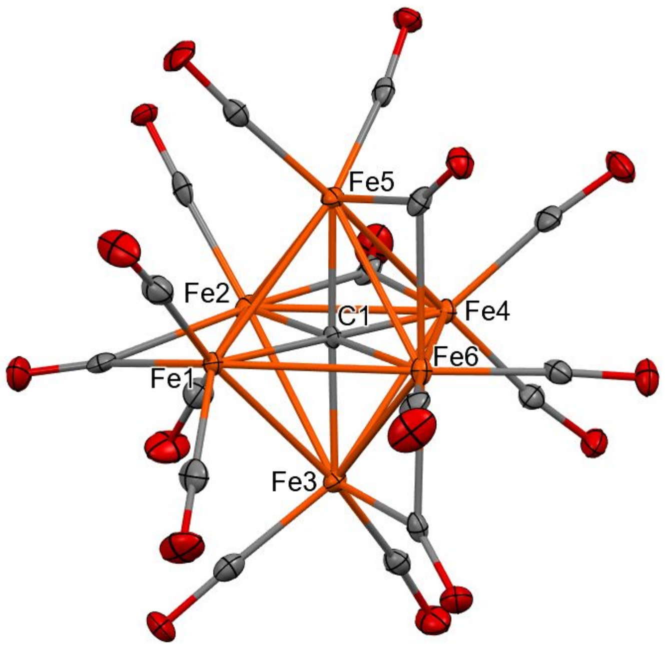

Figure S29. Molecular structure of $[Fe_6C(CO)_{16}]^{2-}$ as found in $[NMe_3CH_2Ph]_2[Fe_6C(CO)_{16}]$ (orange, Fe; red, O; grey, C). Thermal ellipsoids are at the 30% probability level.

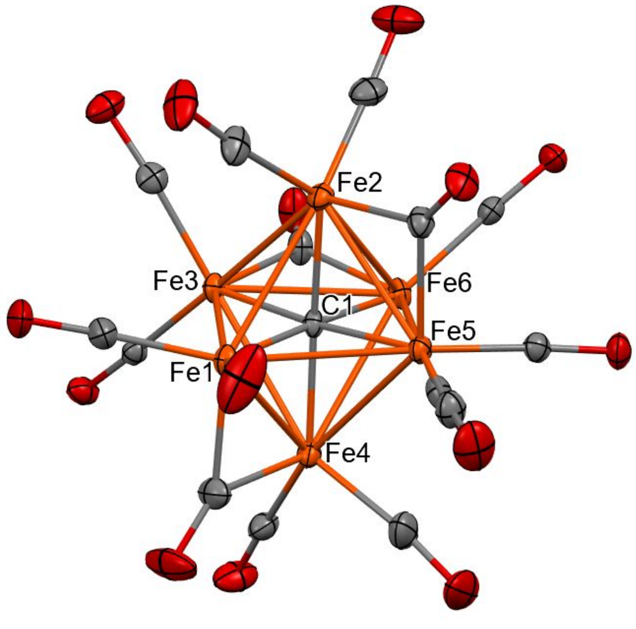

Figure S30. Molecular structure of $[Fe_6C(CO)_{15}]^{4-}$ as found in $[NMe_3CH_2Ph]_4[Fe_6C(CO)_{15}]$ (polymorph 1, space group *C*2/*c*) (orange, Fe; red, O; grey, C). Thermal ellipsoids are at the 30% probability level.

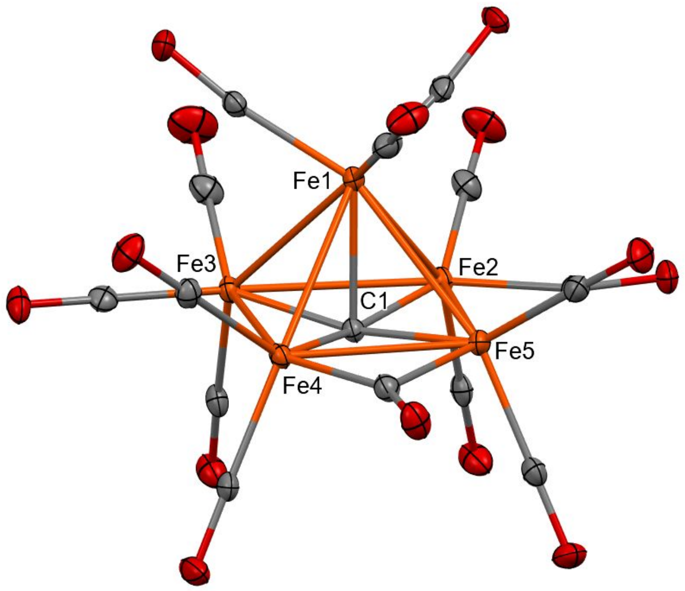

Figure S31. Molecular structure of $[Fe_5C(CO)_{14}]^{2-}$ as found in $[NMe_3CH_2Ph]_2[Fe_5C(CO)_{14}]$ (polymorph 1, space group $P\bar{1}$) (orange, Fe; red, O; grey, C). Thermal ellipsoids are at the 30% probability level.

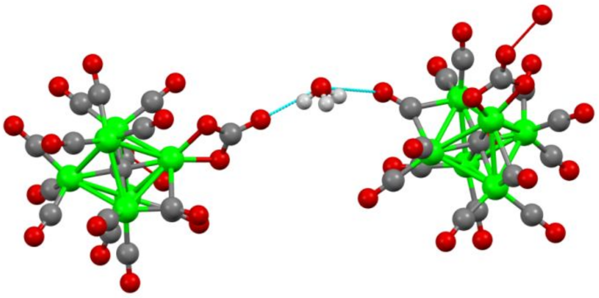

(a)

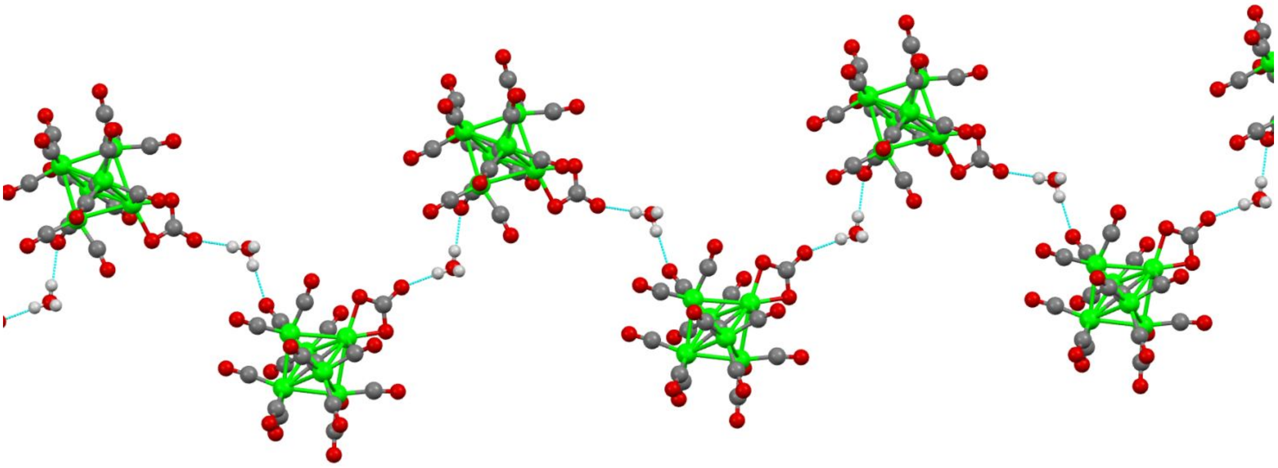

(b)

Figure S32. Hydrogen bonds of $[NEt_4]_3[H_3O][Fe_6C(CO)_{14}(CO_3)]$: (a) a single unit where $[H_3O]^+$ is H-bonded to two $[Fe_6C(CO)_{14}(CO_3)]^{4-}$ via the one CO_3^{2-} and one μ-CO ligand; (b) a piece of the resulting infinite H-bonded chain (green, Fe; red, O; grey, C; white, H).

Table S1. Main bond distances (Å) of $[NEt_4]_3[H_3O][Fe_6C(CO)_{14}(CO_3)]$ (two independent crystals have been collected), $[NEt_4]_2[Fe_6C(CO)_{15}(PTA)]$, $[NMe_3CH_2Ph]_4[Fe_6C(CO)_{15}]$ (two different polymorphs, both with space group *C*2/*c*), $[NMe_3CH_2Ph]_2[Fe_6C(CO)_{16}]$.

	**Fe-Fe**	**Fe-C$_{carbide}$**
$[NEt_4]_3[H_3O][Fe_6C(CO)_{14}(CO_3)]$ Crystal 1*	2.5689(4)-2.7915(4) Average 2.6886(14)	1.876(2)-1.9443(19) Average 1.902(5)
$[NEt_4]_3[H_3O][Fe_6C(CO)_{14}(CO_3)]$ Crystal 2*	2.5674(7)-2.7893(7) Average 2.687(2)	1.869(3)-1.941(3) Average 1.901(7)
$[NEt_4]_2[Fe_6C(CO)_{15}(PTA)]$**	2.559(5)-2.914(5) Average 2.67(2)	1.84(2)-1.93(2) Average 1.89(7)
$[NMe_3CH_2Ph]_4[Fe_6C(CO)_{15}]$ *C*2/*c*, polymorph 1	2.5283(18)-2.7248(18) Average 2.646(7)	1.862(7)-1.882(7) Average 1.87(2)
$[NMe_3CH_2Ph]_4[Fe_6C(CO)_{15}]$ *C*2/*c*, polymorph 2	2.5509(6)-2.7329(6) Average 2.659(2)	1.872(3)-1.890(3) Average 1.880(7)
$[NMe_3CH_2Ph]_2[Fe_6C(CO)_{16}]$	2.5682(11)-2.7114(10) Average 2.664(3)	1.871(5)-1.897(5) Average 1.884(12)

* Two independent crystals of the same compound obtained in two independent batches have been analyzed, in order to get better insights on the bonding parameters of the coordinated carbonate ligand and its H-bond with $[H_3O]^+$.

** Two independent molecules in the unit cell.

Table S2. Main bond distances (Å) and angles (°) of the coordinated CO_3^{2-} anion of $[NEt_4]_3[H_3O][Fe_6C(CO)_{14}(CO_3)]$. Two independent crystals of the same compound obtained in two independent batches have been analyzed, in order to get better insights on the bonding parameters of the coordinated carbonate ligand and its H-bond with $[H_3O]^+$.

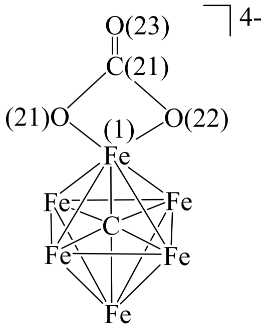	**Crystal 1**	**Crystal 2**
Fe(1)-O(21)	2.0344(14)	2.016(3)
Fe(1)-O(22)	2.0186(14)	2.032(3)
C(21)-O(21)	1.305(2)	1.296(4)
C(21)-O(22)	1.304(2)	1.307(4)
C(21)-O(23)	1.245(2)	1.244(4)
O(21)-Fe(1)-O(22)	65.03(6)	64.89(10)
Fe(1)-O(21)-C(21)	90.39(11)	91.4(2)
Fe(1)-O(22)-C(21)	91.12(11)	90.4(2)
O(21)-C(21)-O(22)	113.30(17)	113.1(3)
O(21)-C(21)-O(23)	123.38(18)	123.6(3)
O(22)-C(21)-O(23)	123.32(18)	123.3(3)

Table S3. Hydrogen bonds (Å and °) of $[NEt_4]_3[H_3O][Fe_6C(CO)_{14}(CO_3)]$.

D-H···A	d(D-H)	d(H···A)	d(D···A)	<(DHA)
**Crystal 1**				
O(101)-H(101)···O(23)	0.848(10)	1.856(18)	2.678(3)	163(5)
O(101)-H(102)···O(12)#1	0.848(10)	2.09(2)	2.879(3)	154(4)
O(111)-H(111)···O(23)	0.845(10)	2.00(6)	2.710(8)	142(9)
O(111)-H(112)···O(12)#1	0.847(10)	2.13(7)	2.774(8)	133(9)
**Crystal 2**				
O(101)-H(101)···O(23)	1.007(19)	1.664(19)	2.670(6)	179(6)
O(101)-H(102)···O(12)#1	1.008(19)	1.91(3)	2.875(5)	160(5)
O(111)-H(111)···O(23)	1.00(2)	1.94(17)	2.716(16)	133(18)
O(111)-H(112)···O(12)#1	1.00(2)	2.18(17)	2.748(15)	114(13)

Symmetry transformations used to generate equivalent atoms: #1 x+1/2, -y+1/2, z-1/2. Two independent crystals of the same compound obtained in two independent batches have been analyzed, in order to get better insights on the bonding parameters of the coordinated carbonate ligand and its H-bond with $[H_3O]^+$.

Table S4. Main bond distances (Å) of $[NMe_3CH_2Ph]_2[Fe_5C(CO)_{14}]$ (two different polymorphs, both with space group $P\bar{1}$), $[NEt_4]_3[Fe_5C(CO)_{13}(COMe)]$ (two polymorphs, space group *P*1 and *C*2), $[NMe_3CH_2Ph]_3[Fe_5C(CO)_{13}(COMe)]$, and $[NEt_4]_2[Fe_5C(CO)_{13}(PPh_3)]$.

	Fe-Fe	Fe-$C_{carbide}$	Fe-COMe
$[NMe_3CH_2Ph]_2[Fe_5C(CO)_{14}]$ $P\bar{1}$, polymorph 1	2.397(2)-2.6775(14) Average 2.589(5)	1.772(10)-1.972(8) Average 1.858(19)	-
$[NMe_3CH_2Ph]_2[Fe_5C(CO)_{14}]$ $P\bar{1}$, polymorph 2	2.5464(6)-2.7157(6) Average 2.6138(17)	1.859(3)-1.997(3) Average 1.894(7)	-
$[NEt_4]_3[Fe_5C(CO)_{13}(COMe)]$* *P*1	2.531(4)-2.760(4) Average 2.635(16)	1.765(19)-1.995(18) Average 1.89(6)	1.91(2) and 1.96(2)
$[NEt_4]_3[Fe_5C(CO)_{13}(COMe)]$ *C*2	2.531(6)-2.746(4) Average 2.638(11)	1.73(2)-2.07(2) Average 1.91(4)	1.93(3)
$[NMe_3CH_2Ph]_3$ $[Fe_5C(CO)_{13}(COMe)]$	2.5413(7)-1.7570(6) Average 2.6291(18)	1.825(3)-1.996(3) Average 1.895(7)	1.918(3)
$[NEt_4]_2[Fe_5C(CO)_{13}(PPh_3)]$	2.5314(7)-2.7034(7) Average 2.618(2)	1.851(3)-2.006(3) Average 1.901(7)	-

* Two independent molecules in the unit cell.

X-ray Crystallographic Study.

Crystal data and collection details for $[NEt_4]_3[H_3O][Fe_6C(CO)_{14}(CO_3)]$ (two independent crystals have been collected), $[NEt_4]_2[Fe_6C(CO)_{15}(PTA)]$, $[NMe_3CH_2Ph]_4[Fe_6C(CO)_{15}]$ (two different polymorphs, both with space group *C*2/*c*), $[NMe_3CH_2Ph]_2[Fe_6C(CO)_{16}]$, $[NEt_4]_2[Fe_5C(CO)_{13}(PPh_3)]$, $[NEt_4]_3[Fe_5C(CO)_{13}(COMe)]$ (two polymorphs, space group *P*1 and *C*2), $[NMe_3CH_2Ph]_3[Fe_5C(CO)_{13}(COMe)]$, $[NMe_3CH_2Ph]_2[Fe_5C(CO)_{14}]$ (two different polymorphs, both with space group $P\bar{1}$) are reported in Table S5. The diffraction experiments were carried out on a Bruker APEX II diffractometer equipped with a PHOTON2 detector using Mo–Kα radiation. Data were corrected for Lorentz polarization and absorption effects (empirical absorption correction SADABS).[1] Structures were solved by direct methods and refined by full-matrix least-squares based on all data using F^2.[2] Hydrogen atoms were fixed at calculated positions and refined by a riding model, unless otherwise stated. All non-hydrogen atoms were refined with anisotropic displacement parameters.

$[NEt_4]_3[H_3O][Fe_6C(CO)_{14}(CO_3)]$: Two independent crystals have been collected in order to better elucidate the nature of the compound. The asymmetric unit of the unit cell contains one cluster anion, three $[NEt_4]^+$ cations and one $[H_3O]^+$ cation, all located in general positions. The $[H_3O]^+$ cation is disordered and, thus, it has been split into two positions and refined isotropically using one occupancy factor per disordered group. The H-atoms of the $[H_3O]^+$ cation have been located in the Fourier difference map and refined isotropically with the 1.5-fold thermal parameter of the parent O-atom. Restraints have been applied to the O-H distances [DFIX 0.84 0.01 line in SHELXL]. $[NEt_4]_3[H_3O][Fe_6C(CO)_{14}(CO_3)]$ could be alternatively formulated as $[NEt_4]_3[Fe_6C(CO)_{14}(HCO_3)]\cdot H_2O$. Refinement parameters for these two alternative formulations were very similar, even though $[NEt_4]_3[H_3O][Fe_6C(CO)_{14}(CO_3)]$ refined slightly better than $[NEt_4]_3[Fe_6C(CO)_{14}(HCO_3)]\cdot H_2O$ for both collected crystals. More importantly, the C-O distances of the coordinated anion were more in agreement with CO_3^{2-} than HCO_3^-. Indeed, the uncoordinated C-O distance is shorter than those involving Fe-bonded oxygens, whereas the opposite is usually found for metal-bonded HCO_3^- anions.

$[NEt_4]_2[Fe_6C(CO)_{15}(PTA)]$: The asymmetric unit of the unit cell contains two cluster anions and four $[NEt_4]^+$ cations, all located in general positions. All C, O and N atoms were restrained to have similar thermal parameters (SIMU line in SHELXL, s.u. 0.01). Restraints to bond distances were applied as follow (s.u. 0.02): 1.47 Å for C–N and 1.53 Å for C–C in $[NEt_4]^+$.

$[NMe_3CH_2Ph]_4[Fe_6C(CO)_{15}]$ (polymorph 1, space group *C*2/*c*): The asymmetric unit of the unit cell contains one cluster anion on a general position, half of a cluster anion on a 2-fold axis, and six

$[NMe_3CH_2Ph]^+$ cations (on general positions). The cluster anion located on 2 displays some disorder of the metal cage and CO ligands, which have been split into two positions and refined using one occupancy factor per disordered group. All the atoms of the disordered cluster anion, and all C and N atoms of the $[NMe_3CH_2Ph]^+$ cations were restrained to have similar thermal parameters (SIMU line in SHELXL, s.u. 0.01). The C atoms of the Ph rings were constrained to fit regular hexagons (AFIX 66 line in SHELXL). Restraints to bond distances were applied as follow (s.u. 0.02): 1.47 Å for C–N and 1.51 Å for $C(sp^3)$–$C(sp^2)$ in $[NMe_3CH_2Ph]^+$.

$[NMe_3CH_2Ph]_4[Fe_6C(CO)_{15}]$ (polymorph 2, space group *C*2/*c*): The asymmetric unit of the unit cell contains one cluster anion on a general position, half of a cluster anion on a 2-fold axis, and six $[NMe_3CH_2Ph]^+$ cations (on general positions). Two $[NMe_3CH_2Ph]^+$ cations were disordered and, thus, they have been split into two positions and refined using one occupancy factor per disordered group. All C and N atoms of the $[NMe_3CH_2Ph]^+$ cations were restrained to have similar thermal parameters (SIMU line in SHELXL, s.u. 0.01). The C atoms of the Ph rings were constrained to fit regular hexagons (AFIX 66 line in SHELXL). Restraints to bond distances were applied as follow (s.u. 0.02): 1.47 Å for C–N and 1.51 Å for $C(sp^3)$–$C(sp^2)$ in $[NMe_3CH_2Ph]^+$.

$[NMe_3CH_2Ph]_2[Fe_6C(CO)_{16}]$: The asymmetric unit of the unit cell contains one cluster anion and two $[NMe_3CH_2Ph]^+$ cations, all located on general positions. The metal cage of the cluster anion displays some minor disorder. The six Fe positions have been each split into two positions and the refined occupancy factor of the major image was 0.9634(8). Since the minor image represented less than 4%, it was not possible to locate its CO ligands. Thus, only the CO ligands of the major image have been included in the final model.

$[NEt_4]_2[Fe_5C(CO)_{13}(PPh_3)]$: The asymmetric unit of the unit cell contains one cluster anion (on a general position), one $[NEt_4]^+$ cation (on a general position), and two halves of two $[NEt_4]^+$ cations (located on 2-fold axes). One of these cations is disordered over two symmetry related (by 2) and equally populated positions. The crystal appears to be racemically twinned with refined batch factor 0.0358(14). All C and N atoms of the $[NEt_4]^+$ cations were restrained to have similar thermal parameters (SIMU line in SHELXL, s.u. 0.01). Restraints to bond distances were applied as follow (s.u. 0.02): 1.47 Å for C–N and 1.53 Å for C–C in $[NEt_4]^+$.

$[NEt_4]_3[Fe_5C(CO)_{13}(COMe)]$ (polymorph 1, space group *P*1): The asymmetric unit of the unit cell contains two cluster anions and six $[NEt_4]^+$ cations, all located in general positions. The crystal appears to be non-merohedrally twinned with four components. The TwinRotMat routine of PALTON was used to determine the twinning matrices and to write the reflection data file (.hkl) containing the twin components. Refinement was performed using the instruction HKLF 5 in SHELXL and three BASF parameters, which refined as 0.19(3), 0.09(5) and 0.23(5). All C, O and N

atoms were restrained to have similar thermal parameters (SIMU line in SHELXL, s.u. 0.01). Restraints to bond distances were applied as follow (s.u. 0.02): 1.47 Å for C–N and 1.53 Å for C–C in $[NEt_4]^+$.

$[NEt_4]_3[Fe_5C(CO)_{13}(COMe)]$ (polymorph 2, space group *C*2): The asymmetric unit of the unit cell contains one cluster anion (on a general position), one $[NEt_4]^+$ cation (on a general position), and four halves of $[NEt_4]^+$ cations located on 2-fold axes. The $[NEt_4]^+$ cations located on 2 are disordered over two equally populated and symmetry related positions. The crystals appear to be racemically twinned with refined batch factor 0.40(9). All C, O and N atoms were restrained to have similar thermal parameters (SIMU line in SHELXL, s.u. 0.01). Restraints to bond distances were applied as follow (s.u. 0.02): 1.47 Å for C–N and 1.53 Å for C–C in $[NEt_4]^+$.

$[NMe_3CH_2Ph]_3[Fe_5C(CO)_{13}(COMe)]$: The asymmetric unit of the unit cell contains one cluster anion and three $[NMe_3CH_2Ph]^+$ cations, all located on general positions.

$[NMe_3CH_2Ph]_2[Fe_5C(CO)_{14}]$ (polymorph 1, space group $P\bar{1}$): The asymmetric unit of the unit cell contains two cluster anions and four $[NMe_3CH_2Ph]^+$ cations, all located on general positions. All C, O and N atoms were restrained to have similar thermal parameters (SIMU line in SHELXL, s.u. 0.01).

$[NMe_3CH_2Ph]_2[Fe_5C(CO)_{14}]$ (polymorph 2, space group $P\bar{1}$): The asymmetric unit of the unit cell contains one cluster anion and two $[NMe_3CH_2Ph]^+$ cations, all located on general positions.

Table S5. Crystal data and experimental details for $[NEt_4]_3[H_3O][Fe_6C(CO)_{14}(CO_3)]$ (two independent crystals have been collected in order to better locate the H-atom of the $[H_3O]^+$ cation), $[NEt_4]_2[Fe_6C(CO)_{15}(PTA)]$, $[NMe_3CH_2Ph]_4[Fe_6C(CO)_{15}]$ (two different polymorphs, both with space group *C*2/*c*), $[NMe_3CH_2Ph]_2[Fe_6C(CO)_{16}]$, $[NEt_4]_2[Fe_5C(CO)_{13}(PPh_3)]$, $[NEt_4]_3[Fe_5C(CO)_{13}(COMe)]$ (two polymorphs, space group *P*1 and *C*2), $[NMe_3CH_2Ph]_3[Fe_5C(CO)_{13}(COMe)]$, $[NMe_3CH_2Ph]_2[Fe_5C(CO)_{14}]$ (two different polymorphs, both with space group $P\bar{1}$).

	**$[NEt_4]_3[H_3O]$ $[Fe_6C(CO)_{14}(HCO_3)]$ Crystal 1**	**$[NEt_4]_3[H_3O]$ $[Fe_6C(CO)_{14}(HCO_3)]$ Crystal 2**	**$[NMe_3CH_2Ph]_2[Fe_6C(CO)_{16}]$**
Formula	$C_{40}H_{63}Fe_6N_3O_{18}$	$C_{40}H_{63}Fe_6N_3O_{18}$	$C_{37}H_{32}Fe_6N_2O_{16}$
Fw	1209.03	1209.03	1095.74
T, K	100(2)	100(2)	100(2)
λ, Å	0.71073	0.71073	0.71073
Crystal system	Monoclinic	Monoclinic	Triclinic
Space Group	$P2_1/n$	$P2_1/n$	$P\bar{1}$
a, Å	12.6892(6)	12.6771(11)	10.4928(7)
b, Å	27.7317(12)	27.706(2)	13.6025(10)
c, Å	14.0712(6)	14.0744(12)	15.7983(12)
α, °	90	90	89.606(2)
β, °	91.733(2)	91.765(3)	71.449(2)
γ, °	90	90	71.342(2)
Cell Volume, $Å^3$	4949.3(4)	4941.1(7)	2014.0(3)
Z	4	4	2
D_c, g cm^{-3}	1.623	1.625	1.807
μ, mm^{-1}	1.785	1.788	2.179
F(000)	2496	2496	1104
Crystal size, mm	0.21×0.18×0.14	0.16×0.13×0.11	0.21×0.14×0.11
θ limits, °	1.623-26.999	1.623-25.999	1.589–25.100
Index ranges	$-16 \le h \le 16$<br>$-35 \le k \le 35$<br>$-17 \le l \le 17$	$-15 \le h \le 15$<br>$-34 \le k \le 34$<br>$-17 \le l \le 17$	$-12 \le h \le 12$<br>$-16 \le k \le 16$<br>$-18 \le l \le 18$
Reflections collected	102680	66882	34482

Independent reflections	10754 [R_{int} = 0.0780]	9638 [R_{int} = 0.0588]	7162 [R_{int} = 0.1239]
Completeness to θ max	99.9%	99.6%	99.9%
Data / restraints / parameters	10754 / 9 / 634	9638 / 12 / 638	7162 / 87 / 575
Goodness on fit on F^2	1.076	1.221	1.040
R_1 (I > 2σ(I))	0.0298	0.0489	0.0494
wR_2 (all data)	0.0621	0.0975	0.1423
Largest diff. peak and hole, e Å^{-3}	0.800 / -0.487	0.766 / -0.575	1.472 / –2.062

	**[NMe$_3$CH$_2$Ph]$_4$[Fe$_6$C(CO)$_{15}$] Polymorph 1**	**[NMe$_3$CH$_2$Ph]$_4$[Fe$_6$C(CO)$_{15}$] Polymorph 2**	**[NEt$_4$]$_2$[Fe$_6$C(CO)$_{15}$(PTA)]**
Formula	$C_{56}H_{64}Fe_6N_4O_{15}$	$C_{56}H_{64}Fe_6N_4O_{15}$	$C_{38}H_{52}Fe_6N_5O_{15}P$
Fw	1368.21	1368.21	1184.91
T, K	100(2)	100(2)	100(2)
λ, Å	0.71073	0.71073	0.71073
Crystal system	Monoclinic	Monoclinic	Orthorhombic
Space Group	*C*2/*c*	*C*2/*c*	*Pca*2$_1$
a, Å	37.438(2)	50.568(3)	23.191(2)
b, Å	12.0561(8)	12.2237(7)	16.1799(16)
c, Å	39.107(2)	30.7116(18)	24.854(3)
α, °	90	90	90
β, °	104.829(2)	114.817(2)	90
γ, °	90	90	90
Cell Volume, Å^3	17063.3(18)	17230.5(18)	9326.1(17)
Z	12	12	8
D_c, g cm^{-3}	1.598	1.582	1.688
μ, mm^{-1}	1.561	1.545	1.922
F(000)	8448	8448	4848
Crystal size, mm	0.19×0.16×0.13	0.31×0.24×0.18	0.16×0.13×0.10
θ limits, °	1.780-25.100	1.461-26.000	1.535–25.049
Index ranges	-44 ≤ h ≤ 44	-62 ≤ h ≤ 62	-27 ≤ h ≤ 27

	$-14 \le k \le 14$<br>$-46 \le l \le 46$	$-15 \le k \le 15$<br>$-37 \le l \le 37$	$-19 \le k \le 19$<br>$-29 \le l \le 29$
Reflections collected	103552	115264	92165
Independent reflections	15052 [R_{int} = 0.1312]	16926 [R_{int} = 0.0387]	16326 [R_{int} = 0.0810]
Completeness to θ max	98.9%	100.0%	99.6%
Data / restraints / parameters	15052 / 726 / 1011	16926 / 952 / 1086	16326 / 912 / 1172
Goodness on fit on F^2	1.095	1.047	1.262
R_1 ($I > 2\sigma(I)$)	0.1166	0.0402	0.1161
wR_2 (all data)	0.2631	0.1001	0.2586
Largest diff. peak and hole, e Å^{-3}	1.431 / -1.235	1.147 / -0.654	1.575 / –1.336

	**$[NEt_4]_2[Fe_5C(CO)_{13}(PPh_3)]$**	**$[NEt_4]_3[Fe_5C(CO)_{13}(COMe)]$ Polymorph 1**	**$[NEt_4]_3[Fe_5C(CO)_{13}(COMe)]$ Polymorph 2**
Formula	$C_{48}H_{55}Fe_5N_2O_{13}P$	$C_{40}H_{63}Fe_5N_3O_{14}$	$C_{40}H_{63}Fe_5N_3O_{14}$
Fw	1178.16	1089.18	1089.18
T, K	100(2)	100(2)	100(2)
λ, Å	0.71073	0.71073	0.71073
Crystal system	Tetragonal	Triclinic	Monoclinic
Space Group	$P4_32_12$	$P1$	$C2$
a, Å	18.9054(4)	12.7520(12)	16.1210(11)
b, Å	18.9054(4)	12.9132(12)	19.9850(14)
c, Å	28.0435(6)	14.6027(14)	14.6130(10)
α, °	90	91.582(3)	90
β, °	90	92.193(3)	92.992(2)
γ, °	90	102.120(3)	90
Cell Volume, Å^3	10023.1(5)	2347.7(4)	4701.6(6)
Z	8	2	4
D_c, g cm^{-3}	1.561	1.541	1.539
μ, mm^{-1}	1.509	1.573	1.571
F(000)	4848	1132	2264

Crystal size, mm	0.19×0.14×0.12	0.18×0.16×0.11	0.16×0.13×0.12
θ limits, °	1.523-26.998	1.641-26.056	1.624–25.099
Index ranges	$-24 \le h \le 24$<br>$-24 \le k \le 24$<br>$-35 \le l \le 35$	$-15 \le h \le 15$<br>$-15 \le k \le 15$<br>$-18 \le l \le 18$	$-19 \le h \le 19$<br>$-23 \le k \le 23$<br>$-17 \le l \le 17$
Reflections collected	215507	27123	22795
Independent reflections	10946 [R_{int} = 0.0915]	17065 [R_{int} = 0.0461]	8378 [R_{int} = 0.0670]
Completeness to θ max	99.9%	99.4%	100.0%
Data / restraints / parameters	10946 / 188 / 660	17065 / 603 / 1120	8378 / 755 / 711
Goodness on fit on F^2	1.046	1.123	1.203
R_1 ($I > 2\sigma(I)$)	0.0280	0.0949	0.1220
wR_2 (all data)	0.0750	0.2474	0.2960
Largest diff. peak and hole, e Å^{-3}	0.840 / -0.429	2.369 / -2.168	1.517 / –2.213

	**[NMe$_3$CH$_2$Ph]$_3$[Fe$_5$C(CO)$_{13}$(COMe)]**	**[NMe$_3$CH$_2$Ph]$_2$[Fe$_5$C(CO)$_{14}$] Polymorph 1**	**[NMe$_3$CH$_2$Ph]$_2$[Fe$_5$C(CO)$_{14}$] Polymorph 2**
Formula	$C_{46}H_{51}Fe_5N_3O_{14}$	$C_{35}H_{32}Fe_5N_2O_{14}$	$C_{35}H_{32}Fe_5N_2O_{14}$
Fw	1149.15	983.87	983.87
T, K	100(2)	100(2)	100(2)
λ, Å	0.71073	0.71073	0.71073
Crystal system	Triclinic	Triclinic	Triclinic
Space Group	$P\bar{1}$	$P\bar{1}$	$P\bar{1}$
a, Å	10.5283(6)	12.4172(7)	11.9752(7)
b, Å	11.8941(7)	16.3972(9)	12.2687(7)
c, Å	19.6696(12)	20.4798(11)	15.5057(9)
α, °	99.602(2)	113.317(2)	79.904(2)
β, °	92.256(2)	92.172(2)	74.563(2)
γ, °	96.101(2)	92.624(2)	61.581(2)

Cell Volume, Å^3	2410.8(2)	3818.1(4)	1928.1(2)
Z	2	4	2
D_c, g cm^{-3}	1.583	1.712	1.695
μ, mm^{-1}	1.537	1.924	1.905
F(000)	1180	1992	996
Crystal size, mm	0.18×0.14×0.12	0.21×0.16×0.12	0.18×0.16×0.13
θ limits, °	1.748-25.099	1.644-25.099	1.890-25.497
Index ranges	$-12 \le h \le 12$<br>$-14 \le k \le 14$<br>$-23 \le l \le 23$	$-14 \le h \le 14$<br>$-19 \le k \le 19$<br>$-24 \le l \le 24$	$-14 \le h \le 14$<br>$-14\le k \le 14$<br>$-18 \le l \le 18$
Reflections collected	30043	47858	24640
Independent reflections	8546 [R_{int} = 0.0423]	13570 [R_{int} = 0.0369]	7153 [R_{int} = 0.0441]
Completeness to θ max	99.5%	99.8%	99.7%
Data / restraints / parameters	8546 / 18 / 623	13570 / 451 / 973	7153 / 0 / 511
Goodness on fit on F^2	1.064	1.066	1.071
R_1 ($I > 2\sigma(I)$)	0.0407	0.0926	0.0370
wR_2 (all data)	0.1025	0.2497	0.0987
Largest diff. peak and hole, e Å^{-3}	1.000 / -0.431	3.874 / -2.667	0.971 / -0.609

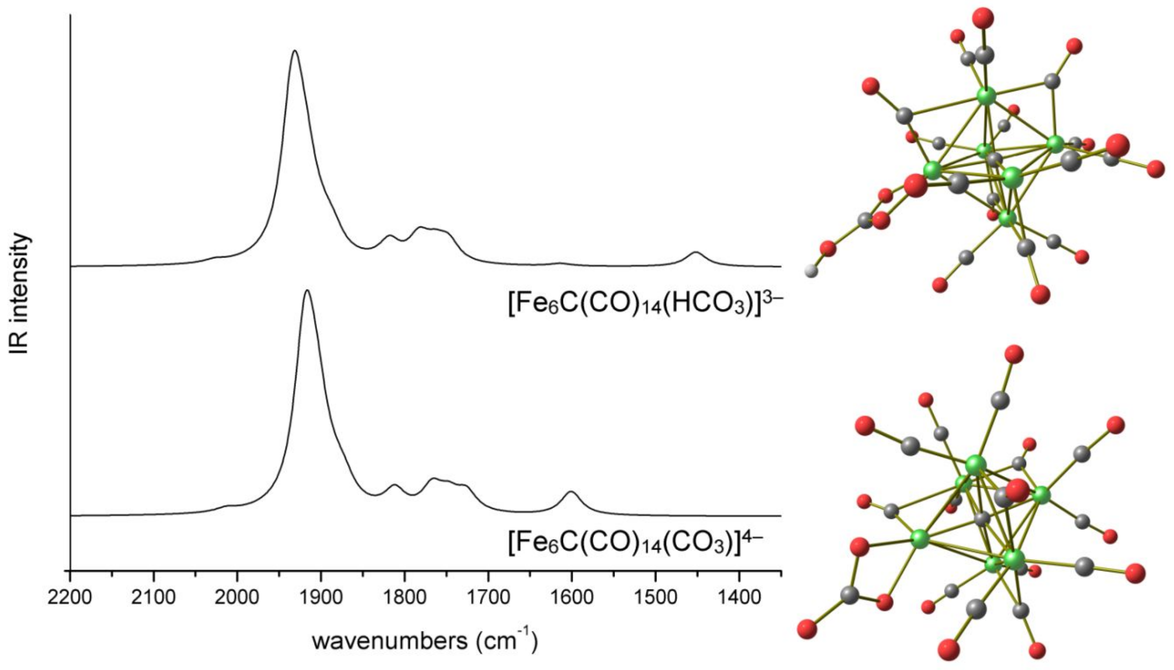

Figure S33. Carbonyl and carbonate stretching region of the unscaled simulated IR spectra (Lorenzian interpolation, FWHM = 30 cm^{-1}) of $[Fe_6C(CO)_{14}(CO_3)]^{4-}$ and $[Fe_6C(CO)_{14}(HCO_3)]^{3-}$ with related DFT-optimized structures (green, Fe; red, O; grey, C; white, H).

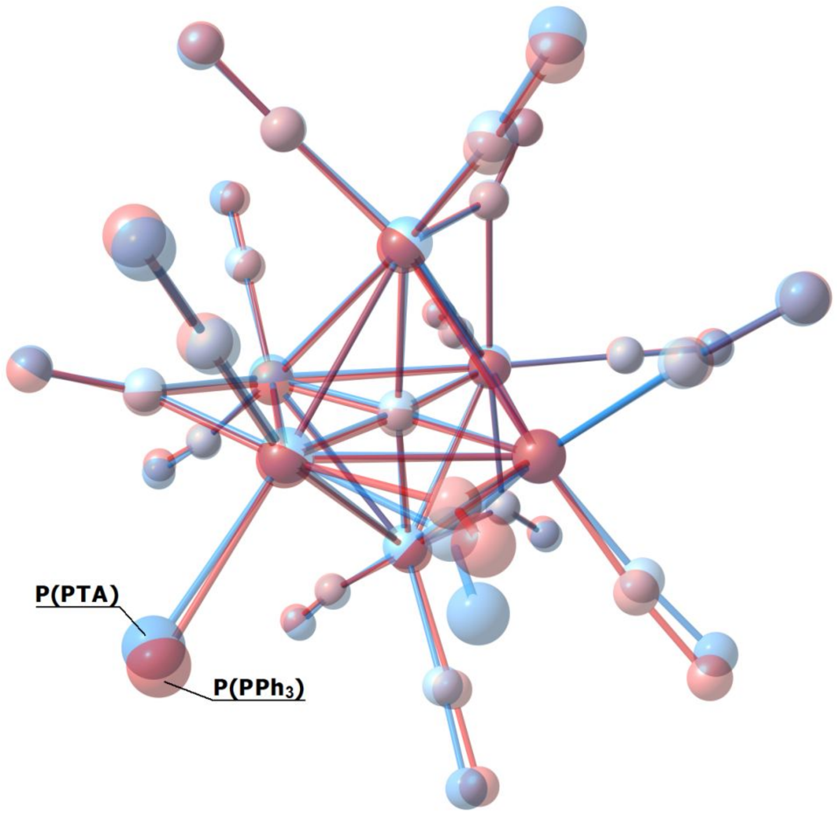

Figure S34. Best superposition of the $\{Fe_6C(CO)_{15}P\}$ fragments of $[Fe_6C(CO)_{15}(PPh_3)]^{2-}$ (red tones) and $[Fe_6C(CO)_{15}(PTA)]^{2-}$ (blue tones).

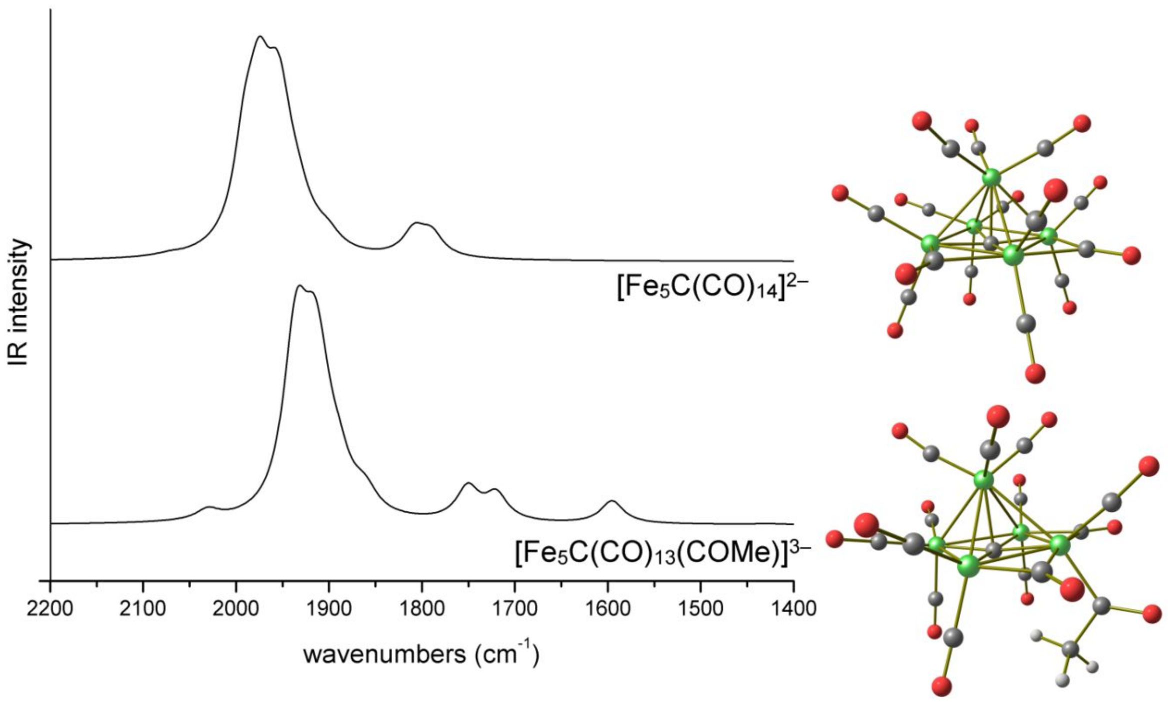

Figure S35. Carbonyl stretching region of the unscaled simulated IR spectra (Lorenzian interpolation, FWHM = 30 cm^{-1}) of $[Fe_5C(CO)_{13}(COMe)]^{3-}$ and $[Fe_5C(CO)_{14}]^{2-}$ with related DFT-optimized structures (green, Fe; red, O; grey, C; white, H).

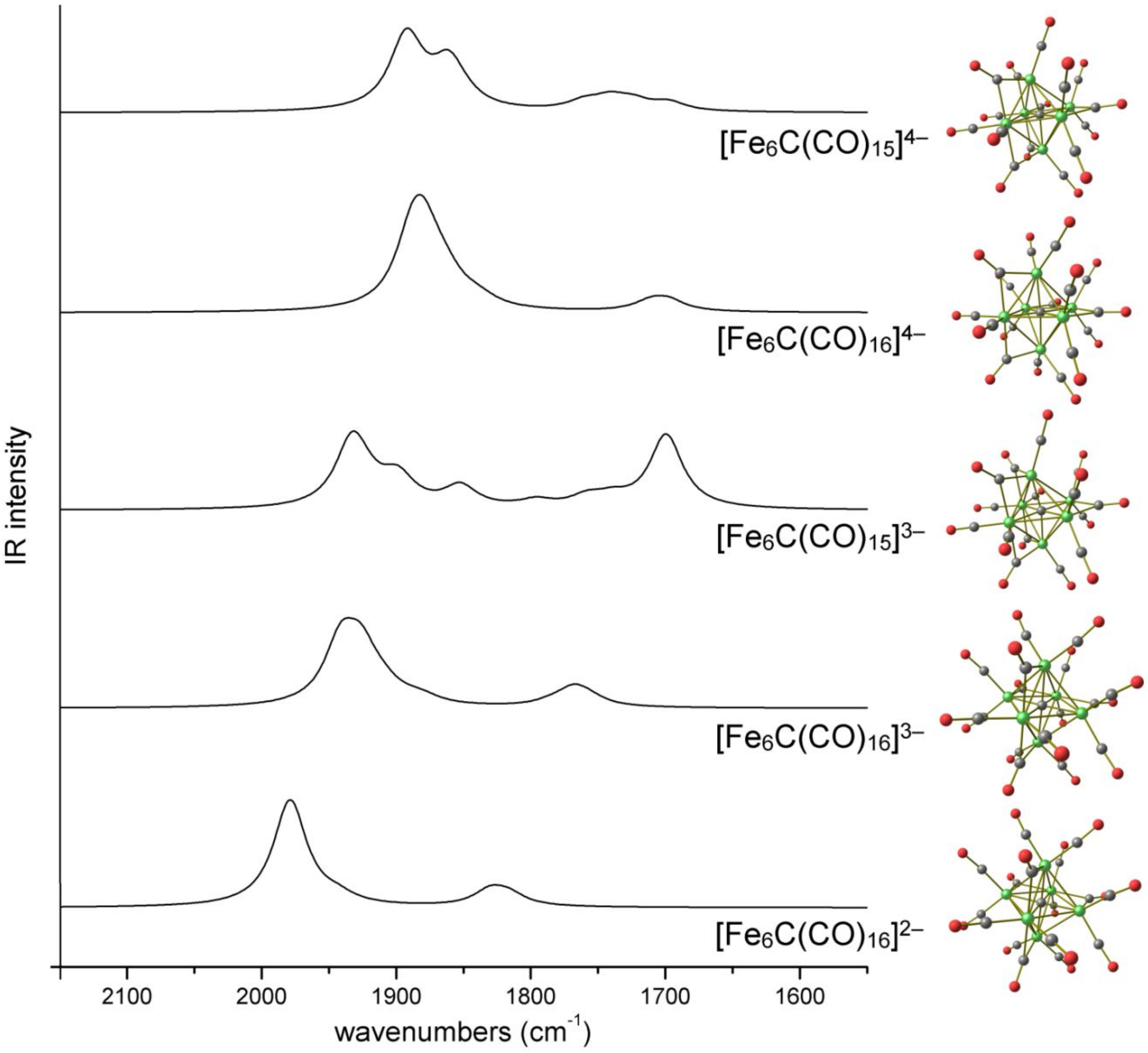

Figure S36. Carbonyl stretching region of the unscaled simulated IR spectra (Lorenzian interpolation, FWHM = 30 cm^{-1}) of $[Fe_6C(CO)_{16}]^{2-}$, $[Fe_6C(CO)_{16}]^{3-}$, $[Fe_6C(CO)_{15}]^{3-}$, $[Fe_6C(CO)_{16}]^{4-}$ and $[Fe_6C(CO)_{15}]^{4-}$ with related DFT-optimized structures (green, Fe; red, O; grey, C).

Table S6. Root-mean-square deviation (RMSD) values (Å) between the DFT-optimized geometries of selected clusters.

**Cluster 1**	**Cluster 2**	**RMSD**
[Fe6C(CO)$_{16}$]$^{2-}$	$[Fe_6C(CO)_{16}]^{3-}$	0.051
$[Fe_6C(CO)_{16}]^{3-}$	$[Fe_6C(CO)_{16}]^{4-}$	0.050
$[Fe_6C(CO)_{15}]^{3-}$	$[Fe_6C(CO)_{15}]^{4-}$	0.099
$[Fe_6C(CO)_{15}]^{2-}$	$[Fe_6C(CO)_{15}]^{3-}$	0.350

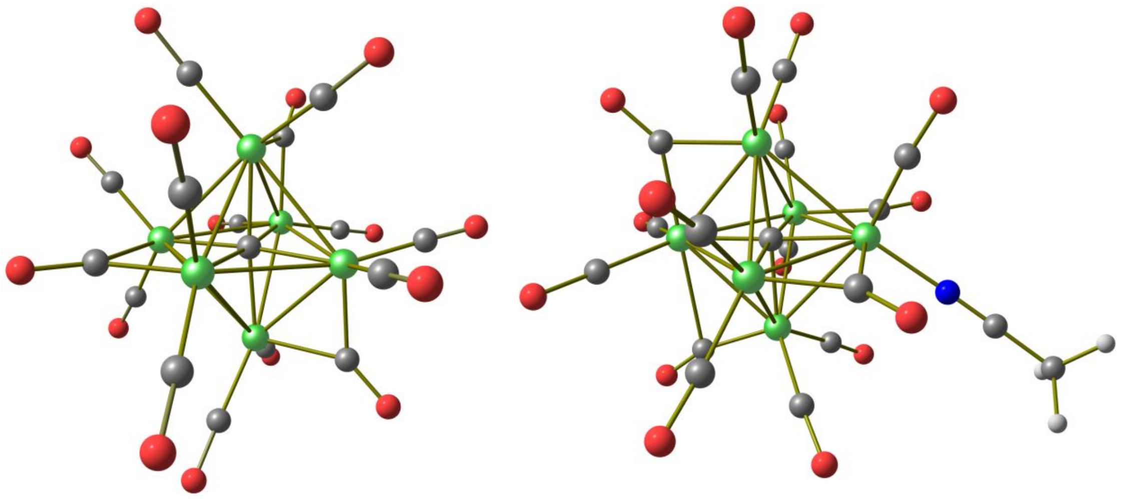

Figure S37. DFT-optimized structures of $[Fe_6C(CO)_{15}]^{2-}$ and $[Fe_6C(CO)_{15}(MeCN)]^{2-}$ (green, Fe; red, O; blue, N; grey, C).

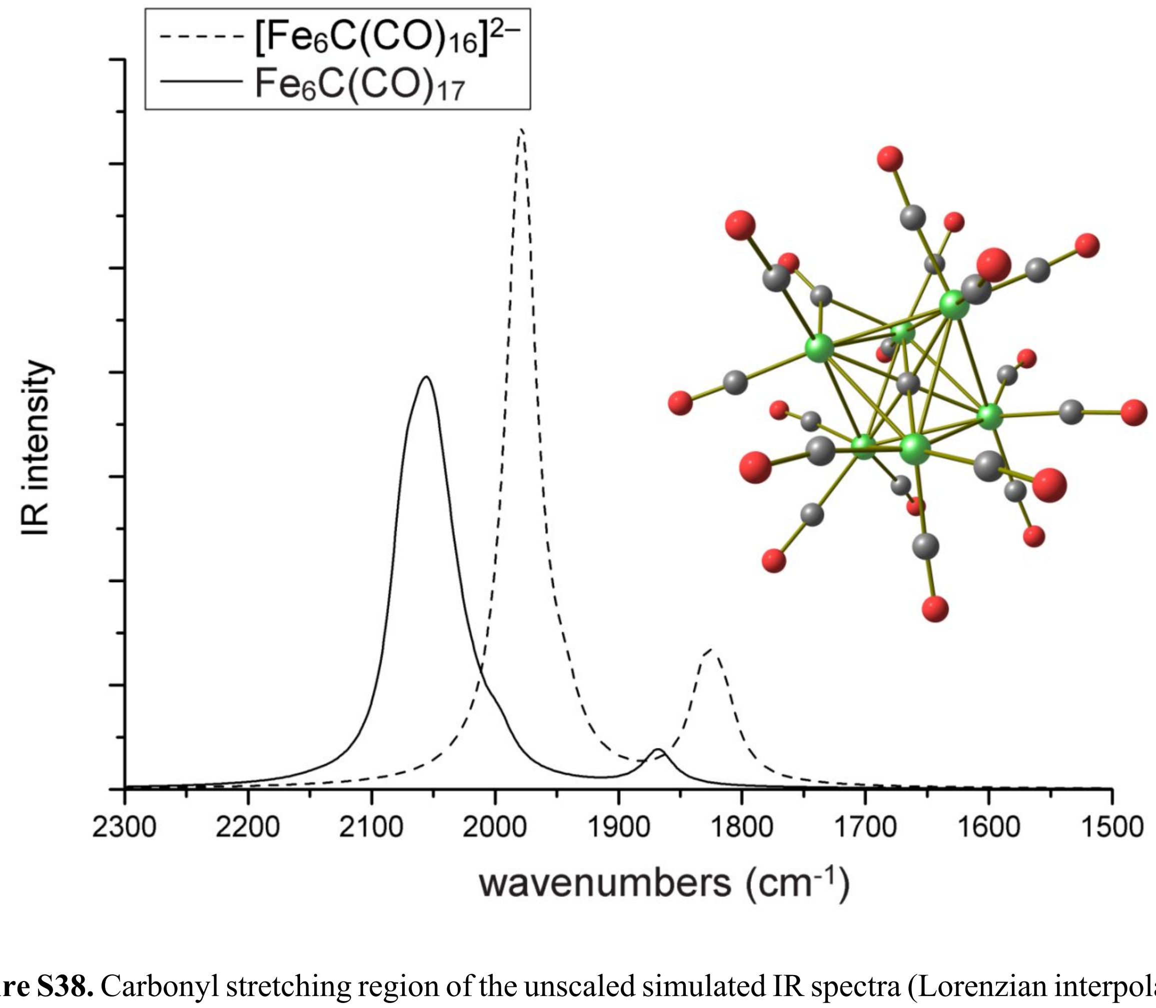

Figure S38. Carbonyl stretching region of the unscaled simulated IR spectra (Lorenzian interpolation, FWHM = 30 cm^{-1}) of $[Fe_6C(CO)_{16}]^{2-}$ and $Fe_6C(CO)_{17}$ with DFT-optimized structure of $Fe_6C(CO)_{17}$ (green, Fe; red, O; grey, C).

==
INPUT FILE for **[$Fe_6C(CO)_{14}(CO_3)]^{4-}$**
==

```
Opt freq  r2scan-3c
%PAL nprocs 8 end
%output
print[p_mos] 1
end #output
%cpcm
epsilon 38.80
refrac 1.3441
end
%scf
guess hcore
end
* xyz -4   1
Fe      1.009242000     -1.049368000     -1.218571000
Fe     -0.460055000     -1.513042000      1.067657000
Fe      1.544670000      0.325570000      1.014492000
Fe      0.248238000      1.625077000     -0.970046000
Fe     -1.577239000     -0.371882000     -0.944589000
Fe     -1.057192000      1.071275000      1.230667000
C       0.000000000      0.000000000      0.000000000
C       0.455642000     -2.991973000      0.776797000
C      -0.936316000     -2.069894000      2.670899000
C       1.816647000     -0.605661000      2.514358000
C       2.849374000     -0.453691000      0.062448000
C       2.444642000      1.833897000      1.369205000
C       0.424165000      3.324753000     -0.481155000
C      -1.050143000      1.846099000     -2.168786000
C       1.563312000      1.594018000     -2.168364000
C      -3.245528000      0.211315000     -1.048106000
C      -1.527463000     -1.314760000     -2.430391000
C      -2.051986000     -1.959800000      0.130521000
C      -2.340450000      0.315032000      2.195229000
C      -0.227885000      1.897957000      2.570477000
C      -2.042490000      2.454532000      0.709990000
O       1.037415000     -3.987819000      0.760904000
O      -1.223374000     -2.496685000      3.707754000
O       2.108806000     -1.169169000      3.473013000
O       3.907212000     -0.801169000     -0.279466000
O       3.037853000      2.791473000      1.579192000
O       0.519423000      4.432080000     -0.203095000
O      -1.718758000      2.246267000     -3.027862000
O       2.388757000      1.731567000     -2.953037000
O      -4.338505000      0.569886000     -1.113366000
O      -1.579728000     -1.971170000     -3.387076000
O      -2.939500000     -2.746825000      0.118988000
O      -3.246746000     -0.053800000      2.796076000
O       0.163557000      2.505004000      3.468653000
O      -2.724628000      3.332795000      0.432070000
C       1.768461000     -2.424860000     -3.064712000
O       1.422440000     -2.918207000     -1.907603000
O       1.739553000     -1.122025000     -3.099030000
O       2.085823000     -3.127859000     -4.042072000
*
```

==
INPUT FILE for **[Fe$_6$C(CO)$_{14}$(HCO$_3$)]$^{3-}$**
==

```
! Opt freq r2scan-3c
%PAL nprocs 8 end
%output
print[p_mos] 1
end #output
%cpcm
epsilon 38.80
refrac 1.3441
end
%scf
guess hcore
end
* xyz -3 1
Fe   1.009242   -1.049368   -1.218571
Fe  -0.460055   -1.513042    1.067657
Fe   1.544670    0.325570    1.014492
Fe   0.248238    1.625077   -0.970046
Fe  -1.577239   -0.371882   -0.944589
Fe  -1.057192    1.071275    1.230667
C    0.000000    0.000000    0.000000
C    0.455642   -2.991973    0.776797
C   -0.936316   -2.069894    2.670899
C    1.816647   -0.605661    2.514358
C    2.849374   -0.453691    0.062448
C    2.444642    1.833897    1.369205
C    0.424165    3.324753   -0.481155
C   -1.050143    1.846099   -2.168786
C    1.563312    1.594018   -2.168364
C   -3.245528    0.211315   -1.048106
C   -1.527463   -1.314760   -2.430391
C   -2.051986   -1.959800    0.130521
C   -2.340450    0.315032    2.195229
C   -0.227885    1.897957    2.570477
C   -2.042490    2.454532    0.709990
O    1.037415   -3.987819    0.760904
O   -1.223374   -2.496685    3.707754
O    2.108806   -1.169169    3.473013
O    3.907212   -0.801169   -0.279466
O    3.037853    2.791473    1.579192
O    0.519423    4.432080   -0.203095
O   -1.718758    2.246267   -3.027862
O    2.388757    1.731567   -2.953037
O   -4.338505    0.569886   -1.113366
O   -1.579728   -1.971170   -3.387076
O   -2.939500   -2.746825    0.118988
O   -3.246746   -0.053800    2.796076
O    0.163557    2.505004    3.468653
O   -2.724628    3.332795    0.432070
C    1.768461   -2.424860   -3.064712
O    1.422440   -2.918207   -1.907603
O    1.739553   -1.122025   -3.099030
O    2.085823   -3.127859   -4.042072
H    2.649706   -3.397235   -4.822759
*
```

```
==================================================================================
INPUT FILE for [Fe5C(CO)13(PPh3)]2–
==================================================================================
!  Opt freq  r2scan-3c
%PAL nprocs 8 end
%output
print[p_mos] 1
end #output
%cpcm
epsilon 38.80
refrac 1.3441
end
* xyz -2   1
Fe     -1.565367000     0.688535000     1.048827000
Fe      0.420078000    -0.866434000     1.581373000
Fe     -1.225448000    -1.321109000    -0.514598000
Fe     -0.616694000     1.121847000    -1.368803000
Fe      0.863032000     1.570661000     0.635466000
C       0.000000000     0.000000000     0.000000000
C      -1.290293000     1.087439000     2.754994000
C      -2.991779000    -0.322904000     1.317764000
C      -2.398150000     2.146141000     0.504222000
C       1.373477000     0.140278000     2.677594000
C      -0.407411000    -1.805087000     2.833515000
C      -0.320446000    -2.169961000    -1.785529000
C      -1.829097000    -2.744686000     0.366248000
C      -2.643920000    -0.891956000    -1.496962000
C      -1.910391000     1.983555000    -2.251893000
C       0.271293000     0.749032000    -2.823980000
C       0.411192000     2.654697000    -0.927679000
C       0.796863000     2.925044000     1.757486000
C       2.583423000     1.748183000     0.398498000
O      -1.195199000     1.352871000     3.876173000
O      -3.957656000    -0.892902000     1.615586000
O      -3.010685000     3.106536000     0.303150000
O       1.939505000     0.611212000     3.585081000
O      -0.994424000    -2.326498000     3.672857000
O       0.235939000    -2.680029000    -2.639734000
O      -2.266757000    -3.672752000     0.895990000
O      -3.615658000    -0.709708000    -2.088680000
O      -2.724835000     2.557712000    -2.786682000
O       0.870405000     0.504774000    -3.780264000
O       0.710276000     3.722474000    -1.357866000
O       0.777768000     3.825508000     2.474559000
O       3.720205000     1.917197000     0.256318000
P       2.012858000    -2.341812000     1.157916000
C       1.450422000    -4.067686000     0.872714000
C       1.643825000    -4.708957000    -0.347459000
H       2.112679000    -4.262789000    -1.042096000
C       1.159846000    -5.990743000    -0.564515000
H       1.286513000    -6.402881000    -1.412270000
C       0.505719000    -6.669447000     0.423737000
H       0.199452000    -7.556110000     0.275948000
C       0.295870000    -6.047459000     1.645032000
H      -0.152188000    -6.512532000     2.342754000
C       0.736365000    -4.748658000     1.850591000
H       0.549202000    -4.317615000     2.676472000
C       3.199739000    -2.430856000     2.549154000
C       3.180834000    -3.412046000     3.532640000
H       2.585314000    -4.147466000     3.458885000
C       4.035358000    -3.315629000     4.625495000
H       4.016452000    -3.984880000     5.301343000
C       4.910678000    -2.251255000     4.737669000
H       5.489183000    -2.186976000     5.486431000
```

C	4.946598000	-1.281408000	3.758951000
H	5.553461000	-0.555440000	3.831864000
C	4.099636000	-1.366482000	2.677313000
H	4.126104000	-0.693450000	2.009037000
C	3.194067000	-2.075434000	-0.217337000
C	3.005013000	-1.082901000	-1.156794000
H	2.207206000	-0.568674000	-1.145857000
C	3.982423000	-0.829569000	-2.121771000
H	3.851975000	-0.128178000	-2.749945000
C	5.128090000	-1.587675000	-2.173371000
H	5.789779000	-1.411855000	-2.831272000
C	5.311472000	-2.616129000	-1.254666000
H	6.088484000	-3.158714000	-1.300096000
C	4.360531000	-2.844884000	-0.275948000
H	4.502321000	-3.533041000	0.362883000

*

==
INPUT FILE for **[$Fe_6C(CO)_{15}(PPh_3)$]$^{2-}$**
==

```
! Opt freq  r2scan-3c
%PAL nprocs 8 end
%output
print[p_mos] 1
end #output
%cpcm
epsilon 38.80
refrac 1.3441
end
* xyz -2   1
Fe      1.461789      0.798460     -0.680655
Fe     -0.353958      1.110711      1.530367
Fe     -1.062032      1.184013     -1.059866
Fe     -0.084842     -1.266929     -1.398275
Fe      1.062108     -1.086873      1.030651
Fe     -1.611308     -0.959003      0.661327
C      -0.094844     -0.000473      0.023965
C       2.442878      0.277770     -2.010685
C       2.680968     -0.289736      0.512788
C       0.401235      1.896121     -1.990751
C       0.748149      1.542169      2.804950
C      -1.207043      2.609184      1.792588
C      -1.647402      0.119118      2.400439
C      -1.798092      2.752658     -0.845432
C      -2.230053      0.817959     -2.302037
C      -0.297123     -0.969432     -3.101793
C       0.974628     -2.604682     -1.769647
C      -1.613968     -2.213132     -1.015416
C       1.278489     -1.152826      2.758156
C       1.534663     -2.764422      0.921204
C      -1.635673     -2.406813      1.641623
C      -3.326682     -0.684405      0.429447
O       3.105633     -0.060060     -2.906669
O       3.848780     -0.371207      0.695548
O       0.641640      2.596777     -2.918561
O       1.449206      1.887360      3.668383
O      -1.757263      3.584674      2.105637
O      -2.264945      0.022217      3.405642
O      -2.295279      3.803571     -0.836315
O      -3.054050      0.599205     -3.093029
O      -0.370956     -0.817478     -4.252094
O       1.664727     -3.471367     -2.120366
O      -2.355720     -3.064860     -1.361824
O       1.425128     -1.290676      3.903953
O       1.842810     -3.884928      0.951580
O      -1.691139     -3.362940      2.297871
O      -4.466641     -0.521790      0.285203
P       2.582596      2.637187     -0.235080
C       1.762091      4.447117      0.311945
C       0.512354      6.816594      1.139957
C       2.006862      4.966794      1.584600
C       0.840392      5.072530     -0.510557
C       0.218950      6.256304     -0.105648
C       1.397100      6.155950      1.995719
H       2.469829      4.290889      2.300426
H       0.433100      4.466955     -1.326011
H      -0.655378      6.543647     -0.677756
H       1.450894      6.401368      3.045358
H      -0.124246      7.597633      1.521257
C       3.819183      3.286269     -1.884959
```

C	5.547693	4.046817	-3.905796
C	3.529045	4.431197	-2.616367
C	4.943122	2.504973	-2.144061
C	5.808894	2.885561	-3.165339
C	4.402574	4.812334	-3.635770
H	2.564747	4.893326	-2.443873
H	4.988102	1.551119	-1.605503
H	6.595301	2.191529	-3.421862
H	4.107219	5.636146	-4.266344
H	6.164523	4.281135	-4.757265
C	4.277142	2.854261	1.115299
C	6.291643	3.276164	2.990537
C	4.305481	2.094811	2.269730
C	5.215947	3.873597	0.904143
C	6.235179	4.073325	1.840136
C	5.319848	2.293411	3.203414
H	3.503044	1.350354	2.401753
H	5.093363	4.488542	0.025187
H	6.955755	4.853353	1.663623
H	5.274996	1.647458	4.072009
H	7.062407	3.410863	3.729600

*

==
INPUT FILE for **[$Fe_5C(CO)_{13}(PTA)]^{2-}$**
==

```
! Opt freq r2scan-3c
%PAL nprocs 16 end
%output
print[p_mos] 1
end #output
%cpcm
epsilon 38.80
refrac 1.3441
end
%scf
guess hcore
end
* xyz -2 1
Fe     -1.536037     0.820352     1.095862
Fe      0.434717    -0.814789     1.638441
Fe     -1.396712    -1.328814    -0.331600
Fe     -0.714784     1.029460    -1.356335
Fe      0.932756     1.463360     0.535164
C      -0.089153    -0.057274     0.035331
C      -1.325224     0.882167     2.834552
C      -3.170318     0.188686     1.050953
C      -1.921083     2.494267     0.782304
C       1.484064     0.370257     2.446276
C      -0.115753    -1.685224     3.054923
C      -0.558241    -2.506524    -1.320284
C      -2.169419    -2.442509     0.796296
C      -2.663067    -0.998703    -1.515573
C      -2.089923     1.872113    -2.054526
C      -0.015022     0.528297    -2.863794
C       0.415878     2.494196    -1.078086
C       0.929100     2.893467     1.539415
C       2.627772     1.539630     0.141931
O      -1.306546     1.071786     3.986715
O      -4.305460    -0.078013     1.110535
O      -2.223404     3.615223     0.667532
O       2.129896     0.816634     3.329469
O      -0.509433    -2.211943     4.019115
O      -0.064427    -3.293899    -2.014435
O      -2.716333    -3.176965     1.512332
O      -3.542943    -0.917581    -2.273854
O      -3.007668     2.425578    -2.507964
O       0.439280     0.179656    -3.878304
O       0.745527     3.531295    -1.557815
O       0.906933     3.836136     2.219155
O       3.763895     1.638009    -0.088728
P       2.346716    -2.414356     1.162698
N       3.171221    -4.678198    -0.227144
N       3.904271    -4.518283     2.132351
N       4.940575    -3.072804     0.413372
C       2.046351    -3.737990    -0.128303
H       1.128471    -4.282936     0.128546
H       1.887445    -3.247665    -1.096762
C       2.881936    -3.545473     2.554239
H       3.281002    -2.925024     3.368033
H       1.999293    -4.075410     2.933366
C       4.065993    -1.905780     0.615714
H       3.996725    -1.328179    -0.313658
H       4.498945    -1.256152     1.384956
C       3.404740    -5.382236     1.047118
H       4.157018    -6.161254     0.866355
H       2.469871    -5.857614     1.369320
```

C	5.122693	-3.835085	1.660894
H	5.478441	-3.156922	2.446371
H	5.887998	-4.600889	1.478037
C	4.411355	-3.983089	-0.61717
H	5.176150	-4.745475	-0.815545
H	4.227039	-3.414907	-1.537695

*

==

INPUT FILE for **[$Fe_6C(CO)_{15}(PTA)]^{2-}$**

==

```
! Opt freq r2scan-3c
%PAL nprocs 8 end
%output
print[p_mos] 1
end #output
%cpcm
epsilon 38.80
refrac 1.3441
end
* xyz -2 1
Fe 1.615485000 0.823557000 -0.659874000
Fe -0.192718000 1.174661000 1.519822000
Fe -0.878707000 1.230967000 -1.073444000
Fe 0.036177000 -1.234527000 -1.388841000
Fe 1.166043000 -1.059784000 1.060520000
Fe -1.494660000 -0.873715000 0.669318000
C 0.000000000 0.000000000 0.000000000
C 2.697113000 0.276676000 -1.941097000
C 2.778281000 -0.229755000 0.648690000
C 0.612242000 1.799205000 -2.100163000
C 0.976341000 1.553270000 2.763765000
C -1.083020000 2.651885000 1.794459000
C -1.461033000 0.163417000 2.448119000
C -1.521330000 2.881640000 -0.969306000
C -2.128934000 0.912546000 -2.162298000
C -0.194805000 -0.944906000 -3.086867000
C 1.127082000 -2.538626000 -1.791973000
C -1.525968000 -2.158399000 -1.110974000
C 1.263909000 -1.244234000 2.853239000
C 1.760196000 -2.716605000 0.934511000
C -1.551478000 -2.402715000 1.595627000
C -3.249060000 -0.676320000 0.442401000
O 3.337184000 0.000000000 -2.808502000
O 4.014362000 -0.325216000 0.874861000
O 0.876619000 2.420513000 -3.052071000
O 1.727729000 1.804059000 3.646082000
O -1.674391000 3.635623000 2.013174000
O -2.040809000 0.014562000 3.464648000
O -1.767155000 4.001289000 -0.996645000
O -3.112233000 0.622926000 -2.843297000
O -0.387290000 -0.749130000 -4.232636000
O 1.799621000 -3.402633000 -2.184666000
O -2.321420000 -2.959304000 -1.419163000
O 1.303334000 -1.365584000 3.986582000
O 2.159082000 -3.781243000 0.810241000
O -1.634966000 -3.422049000 2.125017000
O -4.364547000 -0.561443000 0.347956000
P 2.671603000 2.692335000 -0.246054000
N 2.636816000 5.374963000 0.273394000
N 4.369184000 4.606417000 -1.235244000
N 4.547755000 4.143672000 1.128372000
C 1.781068000 4.269875000 0.144153000
H 1.124763000 4.452708000 -0.574127000
H 1.277824000 4.153380000 0.989189000
C 3.724474000 3.384835000 -1.593141000
H 4.415566000 2.716605000 -1.836710000
H 3.163252000 3.540162000 -2.393440000
C 3.949427000 2.786179000 1.076178000
H 3.536627000 2.572604000 1.951039000
H 4.659071000 2.119567000 0.899715000
```

C	3.353418000	5.638695000	-0.922083000
H	3.801004000	6.517264000	-0.840065000
H	2.711027000	5.695325000	-1.672674000
C	5.248123000	4.498012000	-0.064620000
H	5.941534000	3.815220000	-0.243569000
H	5.704985000	5.363637000	0.077048000
C	3.571414000	5.169478000	1.426620000
H	4.037553000	6.018923000	1.627937000
H	3.054254000	4.905745000	2.229404000

*

==
INPUT FILE for **[$Fe_5C(CO)_{13}(COMe)]^{3-}$**
==

```
! Opt freq r2scan-3c
%PAL nprocs 8 end
%output
print[p_mos] 1
end #output
%cpcm
epsilon 38.80
refrac 1.3441
end
%scf
guess hcore
end
* xyz -3 1
Fe   -1.095207000   -1.555297000   -0.309585000
Fe   -0.135856000   -0.435613000    1.767089000
Fe    1.363825000    1.267583000    0.327009000
Fe    0.120268000    0.084302000   -1.830401000
Fe    1.566613000   -1.235562000   -0.062150000
C     0.000000000    0.000000000    0.000000000
C    -1.244689000   -3.286332000   -0.468927000
C    -1.471618000   -1.758690000    1.692742000
C     0.763597000   -0.996496000    3.192455000
C    -1.077267000    0.884215000    2.466606000
C     0.443214000    2.768091000    0.233301000
C     2.121509000    1.488858000    1.916557000
C     2.671556000    1.710684000   -0.774833000
C    -0.996070000    1.356005000   -2.271641000
C     1.050480000    0.163782000   -3.327209000
C    -1.042411000   -1.382901000   -2.252280000
C     2.246392000   -1.619034000   -1.638532000
C     3.082904000   -0.888622000    0.753922000
C     1.334883000   -2.828858000    0.615490000
C    -2.980585000   -1.260407000   -0.502809000
C    -3.439857000    0.140151000   -0.125267000
H    -3.101563000    0.783740000   -0.783158000
H    -3.095287000    0.369539000    0.761860000
H    -4.419850000    0.171169000   -0.113263000
O    -1.285391000   -4.436008000   -0.569798000
O    -2.222348000   -2.397614000    2.337468000
O     1.366416000   -1.354327000    4.103396000
O    -1.657232000    1.799367000    2.860605000
O    -0.115840000    3.778142000    0.151791000
O     2.641934000    1.676830000    2.932435000
O     3.532743000    2.029013000   -1.479965000
O    -1.742970000    2.199704000   -2.545020000
O     1.657007000    0.212002000   -4.314046000
O    -1.505138000   -1.909770000   -3.203492000
O     2.844641000   -1.989355000   -2.565155000
O     4.106299000   -0.801228000    1.275897000
O     1.329806000   -3.891995000    1.095645000
O    -3.831528000   -2.038062000   -0.912103000
*
```

===

INPUT FILE for **[$Fe_6C(CO)_{16}]^{2-}$**

===

```
! Opt freq r2scan-3c
%PAL nprocs 8 end
%output
print[p_mos] 1
end #output
%cpcm
epsilon 38.80
refrac 1.3441
end
* xyz -2  1
C    -1.740080000    0.608092000    2.099293000
C    -3.061221000   -0.670583000   -0.000041000
C     0.020260000   -0.031295000    0.000001000
C    -0.040951000   -1.207408000    3.054379000
C     0.912874000    1.023513000    2.899635000
C    -1.053811000   -2.914220000    1.196460000
C     1.348887000   -2.446517000    0.000004000
C     2.930946000   -0.703837000    1.300868000
C     2.257715000    1.908468000   -0.000040000
C     0.061414000    2.912554000    1.228580000
C    -2.714634000    1.956807000    0.000052000
Fe   -0.049759000    0.041609000    1.850283000
Fe   -0.399284000   -1.841246000    0.000001000
Fe    1.775477000   -0.561584000    0.000004000
Fe    0.509094000    1.773761000   -0.000009000
Fe   -1.758426000    0.489287000    0.000011000
O    -2.574334000    0.932173000    2.854805000
O    -3.952724000   -1.392981000   -0.000093000
O     0.017731000   -1.974473000    3.906717000
O     1.577847000    1.631278000    3.614567000
O    -1.517443000   -3.665569000    1.929915000
O     1.952131000   -3.460142000    0.000005000
O     3.688144000   -0.814684000    2.154065000
O     3.341224000    2.311646000   -0.000064000
O    -0.266411000    3.719584000    1.978573000
O    -3.321375000    2.927775000    0.000082000
Fe   -0.049774000    0.041613000   -1.850278000
C    -1.053780000   -2.914241000   -1.196457000
C     2.930994000   -0.703846000   -1.300819000
C     0.061339000    2.912509000   -1.228613000
C    -1.740072000    0.608152000   -2.099286000
C    -0.041035000   -1.207388000   -3.054393000
C     0.912917000    1.023477000   -2.899614000
O    -1.517380000   -3.665611000   -1.929912000
O     3.688228000   -0.814699000   -2.153984000
O    -0.266535000    3.719500000   -1.978626000
O    -2.574317000    0.932283000   -2.854786000
O     0.017600000   -1.974429000   -3.906756000
O     1.577933000    1.631216000   -3.614528000
*
```

==
INPUT FILE for **$[Fe_6C(CO)_{16}]^{3-}$**
==

```
!  Opt freq  r2scan-3c
! MOREAD
%moinp "Fe6C(CO)16-2meno-r2scan.gbw"
%PAL nprocs 8 end
%output
print[p_mos] 1
end #output
%cpcm
epsilon 38.80
refrac 1.3441
end
* xyz -3   2
C      -1.868088000     0.440388000     2.096894000
C      -3.220629000    -0.653453000     0.000002000
C      -0.003022000    -0.003755000     0.000000000
C       0.092037000    -1.263846000     3.054749000
C       0.659710000     1.097279000     3.043972000
C      -1.050446000    -2.892782000     1.212921000
C       1.408836000    -2.464992000     0.000011000
C       2.918211000    -0.654831000     1.350929000
C       2.324737000     1.631133000     0.000013000
C       0.281600000     3.055680000     1.215591000
C      -2.622964000     1.981982000    -0.000004000
Fe     -0.077365000     0.020723000     1.882208000
Fe     -0.341120000    -1.854572000     0.000002000
Fe      1.825159000    -0.416768000     0.000006000
Fe      0.485772000     1.816947000     0.000003000
Fe     -1.831210000     0.417131000    -0.000008000
O      -2.737036000     0.648394000     2.864441000
O      -4.153208000    -1.342539000     0.000008000
O       0.268024000    -2.051519000     3.888219000
O       1.179771000     1.727930000     3.867477000
O      -1.580488000    -3.623646000     1.941787000
O       2.109196000    -3.412405000     0.000015000
O       3.658710000    -0.816092000     2.228572000
O       3.367214000     2.179007000     0.000021000
O       0.109948000     3.941173000     1.945402000
O      -3.168560000     3.005136000    -0.000004000
Fe     -0.077355000     0.020721000    -1.882209000
C      -1.050424000    -2.892791000    -1.212922000
C       2.918189000    -0.654820000    -1.350936000
C       0.281620000     3.055680000    -1.215589000
C      -1.868091000     0.440377000    -2.096883000
C       0.092045000    -1.263843000    -3.054754000
C       0.659693000     1.097282000    -3.043984000
O      -1.580452000    -3.623663000    -1.941791000
O       3.658679000    -0.816075000    -2.228588000
O       0.109983000     3.941176000    -1.945400000
O      -2.737030000     0.648373000    -2.864444000
O       0.268031000    -2.051513000    -3.888228000
O       1.179734000     1.727939000    -3.867498000
*
```

==
INPUT FILE for **[$Fe_6C(CO)_{15}$]$^{3-}$**
==

```
!  Opt freq  r2scan-3c
! MOREAD
%moinp "Fe6C(CO)15-4meno-r2scan.gbw"
%PAL nprocs 8 end
%output
print[p_mos] 1
end #output
%cpcm
epsilon 38.80
refrac 1.3441
end
* xyz -3   2
Fe     -0.001019000     0.114171000     -1.849737000
Fe      1.265206000     -1.444220000     -0.000194000
Fe      1.384105000     1.236210000     -0.002394000
C      -0.000003000     -0.063261000     -0.000091000
C      -0.001159000     -0.767786000     -3.343320000
C      -1.362220000     1.260652000     -2.142051000
C      1.364591000     1.257955000     -2.139723000
C      2.233762000     -1.984776000     -1.335477000
C      -0.005860000     -2.869470000     0.002300000
C      2.234364000     -1.981552000     1.335900000
C      3.040113000     0.718165000     -0.002329000
C      1.570003000     2.967596000     -0.001443000
O      -0.003819000     -1.382875000     -4.345717000
O      -2.066254000     1.859421000     -2.896483000
O      2.067972000     1.854766000     -2.896564000
O      2.920621000     -2.370064000     -2.206285000
O      -0.008536000     -4.068908000     0.003316000
O      2.921772000     -2.365025000     2.207091000
O      4.198545000     0.519828000     -0.002493000
O      1.733773000     4.127153000     -0.000807000
Fe      0.001708000     0.117218000     1.849260000
Fe     -1.271159000     -1.438806000     0.002435000
Fe     -1.378921000     1.241821000     0.000139000
C      -0.002519000     -0.761534000     3.344715000
C      1.367523000     1.258604000     2.139659000
C      -1.359153000     1.267648000     2.136745000
C      -2.240566000     -1.973251000     1.339568000
C      -2.242741000     -1.975490000     -1.332187000
C      -3.036551000     0.728811000     0.000897000
C      -1.559421000     2.973775000     -0.003923000
O      -0.003125000     -1.374376000     4.348496000
O      2.073918000     1.855844000     2.893118000
O      -2.059736000     1.868701000     2.892846000
O      -2.927995000     -2.354755000     2.211581000
O      -2.931536000     -2.358371000     -2.202527000
O      -4.195750000     0.534874000     0.001406000
O      -1.719560000     4.133831000     -0.006580000
*
```

==
INPUT FILE for **[$Fe_6C(CO)_{16}$]$^{4-}$**
==

```
! Opt freq r2scan-3c
%PAL nprocs 8 end
%output
print[p_mos] 1
end #output
%cpcm
epsilon 38.80
refrac 1.3441
end
* xyz -4 1
C   -1.892058000   0.448425000   2.048743000
C   -3.170228000  -0.734775000   0.000086000
C   -0.002206000  -0.002045000   0.000057000
C    0.002052000  -1.275303000   3.073576000
C    0.578755000   1.140327000   3.059169000
C   -0.969962000  -2.935650000   1.230739000
C    1.445808000  -2.427936000  -0.002524000
C    2.863148000  -0.644710000   1.416062000
C    2.341806000   1.571938000   0.002667000
C    0.360871000   3.061855000   1.232662000
C   -2.527033000   2.045317000   0.000747000
Fe  -0.089781000   0.017144000   1.914161000
Fe  -0.330957000  -1.893301000  -0.000464000
Fe   1.858114000  -0.422462000   0.000178000
Fe   0.506289000   1.849269000   0.000171000
Fe  -1.856124000   0.425717000   0.000380000
O   -2.747332000   0.662581000   2.854456000
O   -4.115198000  -1.432368000  -0.000574000
O    0.148622000  -2.023145000   3.967197000
O    1.045338000   1.756491000   3.943337000
O   -1.474775000  -3.730798000   1.931470000
O    2.124982000  -3.411393000  -0.004272000
O    3.604992000  -0.805839000   2.310610000
O    3.377519000   2.167941000   0.005057000
O    0.227087000   3.989442000   1.939125000
O   -3.073147000   3.085256000   0.000742000
Fe  -0.089853000   0.018909000  -1.913843000
C   -0.973952000  -2.933754000  -1.231012000
C    2.864340000  -0.644020000  -1.414923000
C    0.362936000   3.059728000  -1.234786000
C   -1.892858000   0.447592000  -2.047947000
C    0.003331000  -1.274631000  -3.071528000
C    0.577247000   1.140672000  -3.061443000
O   -1.479658000  -3.727574000  -1.932545000
O    3.606273000  -0.805984000  -2.309270000
O    0.229731000   3.986607000  -1.942287000
O   -2.748770000   0.659792000  -2.853488000
O    0.149087000  -2.023688000  -3.964235000
O    1.044977000   1.754918000  -3.946250000
*
```

===
INPUT FILE for **[$Fe_6C(CO)_{15}$]$^{4-}$**
===

```
Opt freq  r2scan-3c
%PAL nprocs 8 end
%output
print[p_mos] 1
end #output
%cpcm
epsilon 38.80
refrac 1.3441
end
xyz -4   1
Fe     -0.001099000     0.122406000     -1.822837000
Fe      1.235392000     -1.418790000     -0.000405000
Fe      1.367985000     1.203016000     -0.002209000
C     -0.000057000     -0.053905000     -0.000076000
C     -0.002950000     -0.816513000     -3.248770000
C     -1.342276000     1.210513000     -2.142997000
C      1.347061000     1.203314000     -2.141964000
C      2.259380000     -1.935860000     -1.273421000
C     -0.005277000     -2.821430000     0.001616000
C      2.261141000     -1.934133000     1.271855000
C      3.034771000     0.795757000     -0.001793000
C      1.515278000     2.919111000     -0.002029000
O     -0.004631000     -1.470993000     -4.212697000
O     -2.058798000     1.803709000     -2.878650000
O      2.066444000     1.790706000     -2.879726000
O      2.975518000     -2.295014000     -2.112476000
O     -0.007853000     -4.013346000     0.002028000
O      2.978541000     -2.292362000     2.110232000
O      4.181618000     0.613991000     -0.001689000
O      1.643426000     4.069596000     -0.001589000
Fe      0.001359000     0.125488000     1.822412000
Fe     -1.241077000     -1.413913000     0.002629000
Fe     -1.362885000     1.208837000     0.000088000
C     -0.004715000     -0.810060000     3.250554000
C      1.348942000     1.205873000     2.141190000
C     -1.340472000     1.215744000     2.138252000
C     -2.264756000     -1.926770000     1.277543000
C     -2.269521000     -1.929011000     -1.267532000
C     -3.030785000     0.805801000     0.000238000
C     -1.506191000     2.925297000     -0.003392000
O     -0.008623000     -1.462165000     4.216090000
O      2.069623000     1.794964000     2.876100000
O     -2.055233000     1.810045000     2.875059000
O     -2.980514000     -2.283825000     2.117798000
O     -2.988300000     -2.287311000     -2.104669000
O     -4.178220000     0.627747000     0.000389000
O     -1.631874000     4.076011000     -0.006004000
*
```

==
INPUT FILE for **[$Fe_6C(CO)_{15}]^{2-}$**
==

```
!  Opt freq  r2scan-3c
! MOREAD
%moinp "Fe6C(CO)15-4meno-r2scan.gbw"
%PAL nprocs 8 end
%output
print[p_mos] 1
end #output
%cpcm
epsilon 38.80
refrac 1.3441
end
* xyz -2   1
Fe     -0.001019000     0.114171000     -1.849737000
Fe      1.265206000    -1.444220000     -0.000194000
Fe      1.384105000     1.236210000     -0.002394000
C      -0.000003000    -0.063261000     -0.000091000
C      -0.001159000    -0.767786000     -3.343320000
C      -1.362220000     1.260652000     -2.142051000
C       1.364591000     1.257955000     -2.139723000
C       2.233762000    -1.984776000     -1.335477000
C      -0.005860000    -2.869470000      0.002300000
C       2.234364000    -1.981552000      1.335900000
C       3.040113000     0.718165000     -0.002329000
C       1.570003000     2.967596000     -0.001443000
O      -0.003819000    -1.382875000     -4.345717000
O      -2.066254000     1.859421000     -2.896483000
O       2.067972000     1.854766000     -2.896564000
O       2.920621000    -2.370064000     -2.206285000
O      -0.008536000    -4.068908000      0.003316000
O       2.921772000    -2.365025000      2.207091000
O       4.198545000     0.519828000     -0.002493000
O       1.733773000     4.127153000     -0.000807000
Fe      0.001708000     0.117218000      1.849260000
Fe     -1.271159000    -1.438806000      0.002435000
Fe     -1.378921000     1.241821000      0.000139000
C      -0.002519000    -0.761534000      3.344715000
C       1.367523000     1.258604000      2.139659000
C      -1.359153000     1.267648000      2.136745000
C      -2.240566000    -1.973251000      1.339568000
C      -2.242741000    -1.975490000     -1.332187000
C      -3.036551000     0.728811000      0.000897000
C      -1.559421000     2.973775000     -0.003923000
O      -0.003125000    -1.374376000      4.348496000
O       2.073918000     1.855844000      2.893118000
O      -2.059736000     1.868701000      2.892846000
O      -2.927995000    -2.354755000      2.211581000
O      -2.931536000    -2.358371000     -2.202527000
O      -4.195750000     0.534874000      0.001406000
O      -1.719560000     4.133831000     -0.006580000
*
```

==
INPUT FILE for **[$Fe_6C(CO)_{15}(MeCN)]^{2-}$**
==

```
! Opt freq r2scan-3c
%PAL nprocs 8 end
%output
print[p_mos] 1
end #output
%cpcm
epsilon 38.80
refrac 1.3441
end
* xyz -2 1
Fe    8.445432000    6.723021000    15.515273000
Fe    11.318874000    7.217538000    15.622878000
Fe    9.688333000    7.974118000    13.361146000
Fe    7.987761000    9.557791000    15.196742000
Fe    9.554601000    8.495275000    17.400736000
Fe    10.732031000    10.038473000    15.170953000
C    9.622603000    8.351799000    15.362300000
C    6.634769000    6.952993000    15.368117000
C    8.296403000    6.987118000    17.667746000
C    8.624325000    6.280885000    13.463066000
C    12.349314000    6.248249000    14.398592000
C    11.129662000    5.738734000    16.734375000
C    12.776151000    8.181068000    16.314751000
C    10.958152000    7.442660000    12.127776000
C    8.766186000    8.790777000    11.989057000
C    6.758578000    9.997162000    13.883019000
C    6.758529000    10.139793000    16.443727000
C    9.100326000    11.238729000    15.016505000
C    8.829825000    9.773542000    18.530393000
C    10.787287000    8.017703000    18.692537000
C    11.573684000    10.717956000    13.679149000
C    11.649588000    11.154925000    16.325568000
O    5.485695000    6.975728000    15.273183000
O    7.711071000    6.413956000    18.503455000
O    8.260335000    5.413075000    12.762788000
O    13.026277000    5.602211000    13.733312000
O    11.086722000    4.829624000    17.439394000
O    13.732210000    8.595389000    16.806997000
O    11.726971000    7.131767000    11.328486000
O    8.235099000    9.292518000    11.099373000
O    5.972078000    10.289625000    13.097285000
O    5.964549000    10.500430000    17.193866000
O    8.887834000    12.390667000    14.876779000
O    8.442283000    10.575380000    19.260239000
O    11.546307000    7.732496000    19.510118000
O    12.081747000    11.178351000    12.753833000
O    12.212216000    11.850875000    17.049016000
N    8.276985000    4.712649000    15.807575000
C    8.195922000    3.575132000    15.915722000
C    8.104636000    2.137948000    16.037395000
H    8.695895000    1.664288000    15.255314000
H    7.067915000    1.821554000    15.936206000
H    8.480556000    1.820560000    17.008196000
*
```

==
INPUT FILE for **$Fe_6C(CO)_{17}$**
==
```
! opt freq  r2scan-3c
%PAL nprocs 16 end
%output
print[p_mos] 1
end #output
%cpcm
epsilon 38.80
refrac 1.3441
end
* xyz 0   1
Fe     -2.142181000     0.552705000     5.161195000
Fe      0.034375000     1.994739000     4.235398000
Fe     -2.116629000     3.270343000     5.148803000
O      -4.208224000    -1.484157000     4.694192000
O      -1.841451000     5.912429000     3.872329000
O       1.275947000     4.602141000     4.751502000
O      -3.198894000     1.470713000     7.747910000
O       2.138545000     1.373024000     2.269340000
O      -4.445047000     4.610207000     6.303067000
O       1.526789000     0.860381000     6.502415000
O      -0.526033000    -1.543308000     6.455947000
O      -0.572685000     4.197941000     7.462133000
C      -1.841451000     4.752708000     3.872329000
C      -1.841451000     1.901800000     3.872329000
C      -3.562399000     4.065299000     5.851863000
C      -1.112393000    -0.715192000     5.949446000
C       0.892180000     1.235901000     5.636562000
C      -2.746684000     1.250241000     6.723912000
C       0.765543000     3.593882000     4.577093000
C      -1.141244000     3.856478000     6.541138000
C       1.316213000     1.587223000     3.023514000
C      -3.406217000    -0.679342000     4.860547000
Fe     -1.540721000     0.552705000     2.583463000
Fe     -3.717277000     1.994739000     3.509259000
Fe     -1.566273000     3.270343000     2.595854000
O       0.525323000    -1.484157000     3.050466000
O      -4.958848000     4.602141000     2.993155000
O      -0.484008000     1.470713000    -0.003253000
O      -5.821447000     1.373024000     5.475318000
O       0.762146000     4.610207000     1.441591000
O      -5.209691000     0.860381000     1.242243000
O      -3.156869000    -1.543308000     1.288711000
O      -3.110216000     4.197941000     0.282525000
C      -0.120502000     4.065299000     1.892794000
C      -2.570509000    -0.715192000     1.795212000
C      -4.575082000     1.235901000     2.108096000
C      -0.936218000     1.250241000     1.020746000
C      -4.448445000     3.593882000     3.167565000
C      -2.541657000     3.856478000     1.203520000
C      -4.999114000     1.587223000     4.721143000
C      -0.276685000    -0.679342000     2.884111000
*
```

REFERENCES

(1) Sheldrick, G. M. *SADABS-2008/1-Bruker AXS Area Detector Scaling and Absorption Correction*; Bruker AXS: Madison, WI, 2008.

(2) Sheldrick, G. M. Crystal Structure Refinement with SCHELXL. *Acta Crystallogr., Sect. C: Struct. Chem.* **2015**, *71*, 3-8.
